# Which Social, Economic, and Health Sector Strategies Will Deliver the Greatest Impacts for Youth Mental Health and Suicide Prevention? Protocol for an Advanced, Systems Modelling Approach

**DOI:** 10.3389/fpsyt.2021.759343

**Published:** 2021-10-13

**Authors:** Jo-An Occhipinti, Adam Skinner, Louise Freebairn, Yun Ju Christine Song, Nicholas Ho, Kenny Lawson, Grace Yeeun Lee, Ian B. Hickie

**Affiliations:** ^1^Brain and Mind Centre, Faculty of Medicine and Health, University of Sydney, Sydney, NSW, Australia; ^2^Computer Simulation & Advanced Research Technologies (CSART), Sydney, NSW, Australia

**Keywords:** suicide prevention, strategic planning, decision analysis, systems modelling, simulation, mental health

## Abstract

**Background:** Current global challenges are generating extensive social disruption and uncertainty that have the potential to undermine the mental health, wellbeing, and futures of young people. The scale and complexity of challenges call for engagement with systems science-based decision analytic tools that can capture the dynamics and interrelationships between physical, social, economic, and health systems, and support effective national and regional responses. At the outset of the pandemic mental health-related systems models were developed for the Australian context, however, the extent to which findings are generalisable across diverse regions remains unknown. This study aims to explore the context dependency of systems modelling insights.

**Methods:** This study will employ a comparative case study design, applying participatory system dynamics modelling across eight diverse regions of Australia to answer three primary research questions: (i) Will current regional differences in key youth mental health outcomes be exacerbated in forward projections due to the social and economic impacts of COVID-19?; (ii) What combination of social policies and health system strengthening initiatives will deliver the greatest impacts within each region?; (iii) To what extent are optimal strategic responses consistent across the diverse regions? We provide a detailed technical blueprint as a potential springboard for more timely construction and deployment of systems models in international contexts to facilitate a broader examination of the question of generalisability and inform investments in the mental health and wellbeing of young people in the post COVID-19 recovery.

**Discussion:** Computer simulation is known as the third pillar of science (after theory and experiment). Simulation allows researchers and decision makers to move beyond what can be manipulated within the scale, time, and ethical limits of the experimental approach. Such learning when achieved collectively, has the potential to enhance regional self-determination, help move beyond incremental adjustments to the status quo, and catalyze transformational change. This research seeks to advance efforts to establish regional decision support infrastructure and empower communities to effectively respond. In addition, this research seeks to move towards an understanding of the extent to which systems modelling insights may be relevant to the global mental health response by encouraging researchers to use, challenge, and advance the existing work for scientific and societal progress.

## Background

For decades, mental and substance use disorders have been major contributors to disability in young people globally (1–3). Recent years have seen intensifying political polarisation and conflicts, population displacements, extreme weather events and disasters around the world; and now a pandemic has precipitated public health, social and economic crises, undermining confidence in access to, and protection of, the basic human rights to health, housing, education, livelihoods, and futures. These global adversities are being woven into the collective consciousness of young people worldwide, even in places where severe adversity has not been a recent feature of everyday life. Such adversities can take a largely unseen toll during the transition from adolescence to early adulthood, a critical phase of significant biological, cognitive, and social change and the peak period of risk for onset of common mental disorders (4, 5).

Economic recessions represent a particular and significant risk to mental health (6). As demonstrated most markedly during the 2008–2009 global financial crisis, such events have corrosive impacts on the determinants of mental health. For example, almost one-third of young people in Europe remained at risk of poverty, social exclusion, long-term unemployment, underemployment, and in casualised and precarious employment 8 years after the crisis (7). Individual and aggregate studies highlight a strong association between youth unemployment and youth suicidal behaviour (5, 8). Unemployment is thought to increase suicide risk through financial stress and social dislocation (disruption of routines, workplace relationships, and private economy). Analyses at the population level also capture the detrimental impact of unemployment on those who remain employed, but fear losing their jobs (9).

The negative psychological effects of economic recession also impact young people through families. Parental job loss, job instability, low wages, poor work quality, and residential moves can lead to diminished parental emotional investments, increased parental stress and lower parenting quality, marital tension, and increases in child abuse and neglect (10–13). Such adverse early life experiences can become encoded within individual (e.g., brain and cognitive substrates), interpersonal or broader social systems. The behavioural and health consequences of such impacts (e.g., pervasive helplessness or hopelessness, risk-taking behaviours, alcohol and other substance misuse, increased onset of common anxiety and depressive disorders, antisocial behaviour, suicidal thoughts and behaviour, as well as physical ill health) are then likely to be played out over a much longer period (4, 5, 14–22). The advent of the deepest global recession since the Second World War and the compounding effect of social isolation resulting from “lockdowns” or fear of contagion during the pandemic will contribute to an increased risk of adverse mental health outcomes (23, 24).

Many countries and international agencies now recognise the serious, widespread, and uneven impacts COVID-19 and recession are likely to have on population mental health outcomes, mental health services, and suicide risk, particularly for young people, with early signs of the looming threat emerging (24, 25). Uncertainty regarding how the COVID-19 pandemic will evolve, with associated lockdowns, physical distancing, and quarantine measures is driving uncertainty about the extent and duration of the resulting global economic downturn. In addition, it is unknown the extent to which policies aimed at hastening economic recovery will further exacerbate adverse conditions for young people, as seen in the Great Recession, including an increase in precarious employment, a decline in occupational choices, a decline in employment quality and quantity, and greater exposure to future economic shocks across their lifetimes (7). Such exposures have the potential to increase the risk or severity of psychological distress and mental health problems among previously healthy people and especially among those with pre-existing conditions (26). Prior to the pandemic, suicide was the second leading cause of mortality among people aged 15–29 years globally, accounting for *c*. 8% of deaths in this age group every year (27). Attention is turning to appropriate economic, social, and population mental health responses to mitigate these potential adverse impacts, with the World Bank Group committing up to USD $160 billion as a primer to help countries rebuild with stronger, more equitable, and more resilient economic and social systems (28). In addition, individual governments around the world are committing trillions to social and economic aid packages (29). However, a key challenge remains; namely, how best to allocate funding and resources across economic, social, and health systems to deliver the greatest national benefit, and to safeguard the mental health and wellbeing of young people.

To understand how best to respond in complex and uncertain times, decision makers require credible projections of population mental health trajectories and the ability to explore the likely impacts of alternative policies and strategies on those trajectories. Historically, the absence of investment in advanced systems science-based decision support capability in mental health (and public health more broadly with the exception of infectious disease control) has given rise to the popularity of the “comprehensive approach;” an approach that advocates for investment across a broad range of programs, services, and initiatives aimed at addressing the many risk factors contributing to mental disorder and suicidal behaviour, and an approach that assumes more must be better (30). For example, the Australian Government, in their May 2021 budget, committed the largest single mental health and suicide prevention investment in its history, promising $2.3 billion in a whole-of-government and whole-of-community approach to deliver preventive, timely, and effective care (31). This investment is spread across 37 programs, services, and initiatives highlighting the significant challenges associated with investing for impact. While investing in a broad range of initiatives seems like a good idea and gives the impression of a commitment to action, insufficient investment in most or all of those initiatives will likely fail to deliver real impact no matter how large the budget envelope. As an analogy, providing suboptimal doses of chemotherapy to a broad range of people with cancer will not be effective in increasing the recovery rate no matter the budget and resources committed to its implementation. By contrast, systems modelling and simulation can help decision makers identify the few key areas among the overwhelming number of possibilities, where resources can be strategically focused to address a complex issue, and understand the optimal scale, timing, and intensity of investments and actions needed to deliver impact, before implementing them in the real world (30, 32). Systems modelling and simulation can bring advanced forecasting and decision support capability to the challenging and intersecting areas of social, health, and economic policy.

At the outset of the pandemic several systems models were developed for the Australian context capturing the interacting social, economic, and health system drivers of mental health outcomes and suicidal behaviour at a regional, state, and national level (33–35). These models enable decision makers to better understand the likely trajectories of the prevalence of psychological distress, health service engagements and waiting times, mental health-related emergency department (ED) presentations, self-harm hospitalizations, and suicide deaths over the next 10 years. The models have been validated against historic time series data across a range of indicators and provide age- and gender-specific projections of population mental health outcomes. These systems models provided decision analytic capability by simulating prospective impacts of the range of social protection measures and health system strengthening initiatives considered by the Australian Government, informing the national discourse regarding the trade-offs and implications of decision options. However, the extent to which findings regarding best strategic responses are generalisable across diverse regions across Australia and internationally remains unknown due to regional variations in risk-modifying factors, including alcohol consumption, population density, unemployment, poverty and deprivation, interpersonal violence, and differences in service structure and capacity (36–41). This study aims to explore the context dependency of systems modelling insights across diverse regions.

## Methods

### Study Aims, and Research Questions

This study is embedded in a broader program of participatory action research that aims to empower communities to address the mental health needs of young people (42), and evaluate the feasibility, value, impact, and sustainability of building regional capacity in the use of more advanced systems strengthening tools and technologies. A detailed protocol for the evaluation process will be provided elsewhere. This study aims to answer three primary research questions: (i) Will current differences in the prevalence of psychological distress, mental health-related ED presentations, self-harm hospitalizations, and suicide deaths in youth populations across metropolitan, outer urban, regional, and rural and remote areas of Australia be exacerbated in forward projections due to the social and economic impacts of COVID-19? (ii) What combination of social policies and health system strengthening initiatives will deliver the greatest impacts to reduce these mental health and suicide outcomes? (iii) To what extent are optimal strategic responses consistent across diverse contexts? The focus of the current study is an exploration of the generalisability of systems modelling insights across diverse regions of Australia. However, we provide herein a detailed technical blueprint as a potential springboard for more timely construction and deployment of systems models in diverse international contexts, with varying experiences in efforts to control COVID-19 transmission. We encourage the research community to leverage this existing work to undertake a broader examination of the question of generalisability and support the translation of systems modelling insights to global research and policy communities to inform how best to foster the mental health and wellbeing of young people in the post COVID-19 recovery.

### Context and Study Design

In 2015, as part of major reforms to the mental health system, the Australian Government established 31 Primary Health Networks (PHNs) across Australia to decentralise decision making and the implementation of mental health and suicide prevention programs and services to the regional level (43). PHNs are independent not-for-profit primary health care organisations that support the primary care system (including GPs, nurses and allied health practitioners) to improve patient care as well as improve coordination between different parts of the health system, such as between hospitals and community-based mental health care providers (30). The role of PHNs is to commission, rather than provide programs and services, but they work closely with providers to monitor and evaluate performance, implement change and improve the coordination of care (44). This study uses a comparative case study design. Eight sites, primarily defined by PHN boundaries, have been selected (two metropolitan, two outer urban, two regional, and two rural/remote sites) to capture variation in socioeconomic conditions, population density, demographic profile, mental health risk profile, and mental health service infrastructure and access. Our transparent, inclusive, and collaborative approach to the development of systems models (35, 45, 46) will be implemented at each of the eight sites.

### Overall Approach

Of the systems modelling methods, system dynamics (SD) is well-suited to capturing the complex interrelationships and dynamics between and within health, social, and economic systems and by doing so provide a means of understanding and forecasting potential non-linear system behaviour (32, 47). SD models draw on a broad range of aggregate datasets, research evidence, and expert and local knowledge to posit, test, and validate a causal structure that underlies observed data patterns for a broad range of population mental health outcomes (48). From this robust basis, SD models are able to estimate future population mental health trajectories, simulate alternative strategies for the allocation of resources, and identify which policies, reforms, investments and actions are required across which sectors, at what scale, and when, to deliver the best outcomes for youth mental health (49). Interactive interfaces allow direct end-user engagement with SD models, facilitating scenario testing, the testing of alternative assumptions, and transparent strategy dialogues with community and system stakeholders to facilitate consensus building for collaborative action. For these reasons, SD modelling was deemed the most appropriate method to answer the research questions and achieve the broader program objectives of regional empowerment in the use of systems modelling to inform decision making and collective, coordinated action.

A system dynamics model will be developed in partnership with each site over a 6-month period using a participatory model building approach that will include representatives from health service and social policy agencies, youth focussed non-government organisations, primary, secondary, and tertiary education, primary care providers, emergency services, research institutions, community groups, and importantly, young people with lived experience of mental health issues as well as their supportive others (such as a parent). The process will employ a systems perspective to examine the primary local drivers of youth mental health and suicide challenges in each region. Input from stakeholders will be provided through a series of workshops, meetings, priority setting surveys, and system mapping activities (35). A detailed protocol for the participatory model building process will be provided elsewhere. Model structure, parameter estimates, and other numerical inputs will be informed (where possible) by published research and available regional, state, and national data, or will be estimated via constrained optimisation. Model construction and analysis will be performed using Stella Architect ver. 1.9.4 (www.iseesystems.com). All models will be validated by (i) testing whether the outputs of the model can replicate historic data across a range of key indicators (such as time series of psychological distress, psychiatric hospitalisations, ED presentations, youth and total population self-harm hospitalisations, and suicide deaths); and (ii) ensuring face validity of the model structure and performance among stakeholders working in or interacting with different parts of the system.

### Model Structure and Outputs

**Figure 1:**
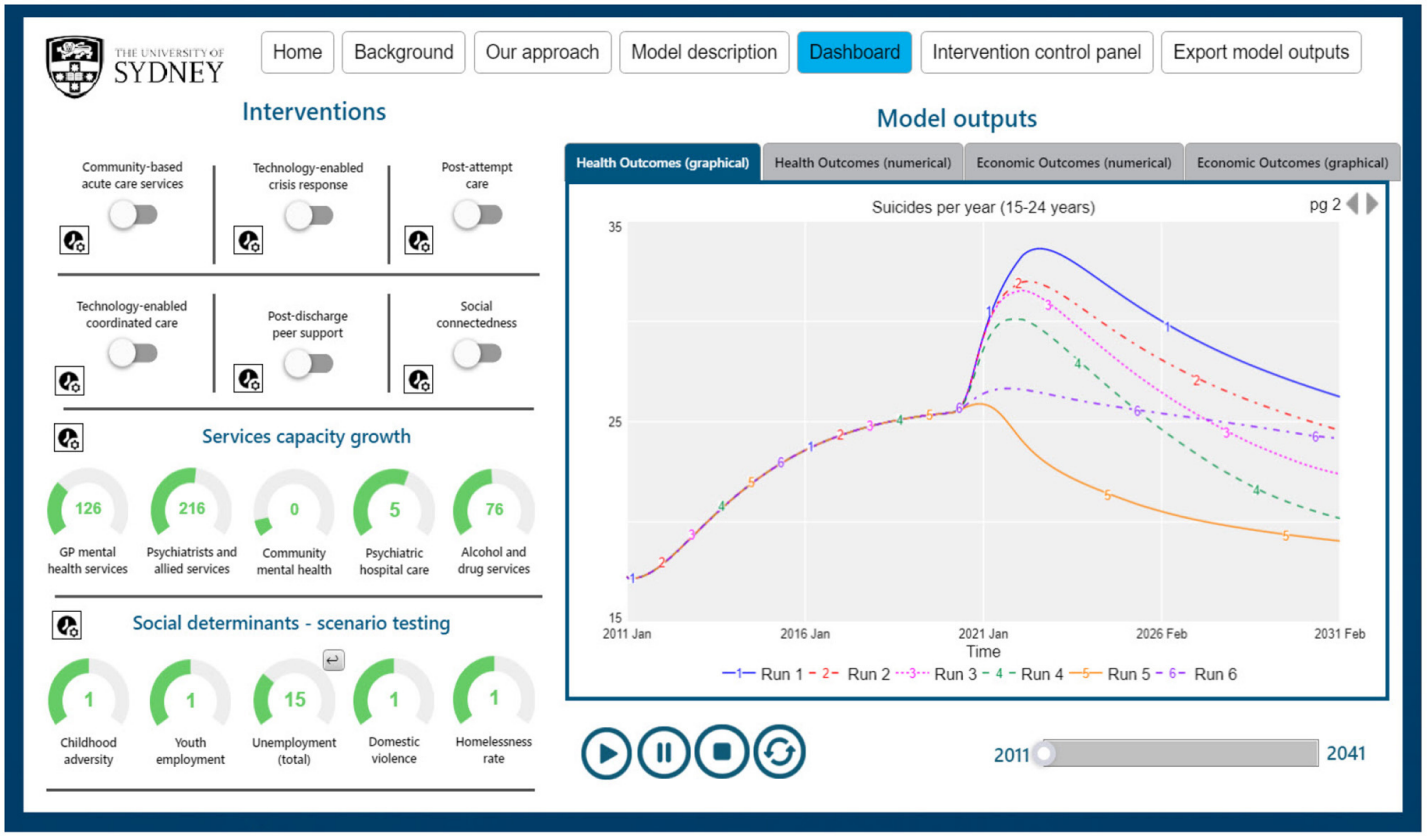
Interactive user interface to facilitate scenario testing and inform decision making.

Model building will draw on foundational work undertaken over the past 5 years applying systems modelling to mental health service planning and suicide prevention (35, 50–53). Based on this existing body of work, the current protocol (along with Additional File 1) provides a comprehensive and detailed operational roadmap for researchers and decision makers to leverage when developing systems models in other contexts. This paper provides example model structure and details model logic, assumptions, parameterisation, calibration, and sensitivity analysis based on the best research evidence available to date. Much of the model structure outlined in Additional File 1 has been implemented and validated at national, state, and regional levels in Australia and is provided in modular form (model components) allowing systems models to be constructed and customised for each of the eight sites of the current study as well as for international applications based on:

- the characteristics and drivers of youth mental health in the context in which the model is being applied. For example, the structure and dynamics of the mental health services component may require modification for each context to ensure it is locally valid. Further, some model components may not be major drivers of youth mental health outcomes in some contexts and hence may be omitted. Additionally, further modules may be required that to date have not been relevant to applications in the Australian context. For example, a component that captures state stability, outbreaks of civil war and the impact on psychological distress may be relevant in other international contexts,
- the key questions the systems modelling may seek to answer. For example, if deemed important by participating stakeholders, and if data availability allows, models may be stratified by different sub-populations (e.g., by Indigenous status, by socioeconomic status, by health sub-catchments, or by remoteness). Such stratifications can capture variation in the risk profiles of subpopulations and allow forecasting of differential impacts of policies, programs, and initiatives on those sub-populations, providing an estimate of changing relative and absolute inequality over time,
- the interventions the models seek to prospectively evaluate. For example, Australian applications of systems modelling have included a range of social, economic, and mental health programs, services and initiatives that represented stakeholder priorities identified by the participatory model building process and usually reflected contemporary national and regional discourse. However, additional policies and initiatives may be of interest in other regional, national, and international applications. The selection of interventions also influences the extent to which components of the model require elaboration. Once developed, models can be extended to incorporate additional interventions over time, based on changes in stakeholder priorities, or on promising interventions identified by new research, and
- preferences for the design of interactive model interfaces. Figure 1 provides an example model interface that can be easily customised to reflect the preferred language, aesthetic characteristics, and graphical vs. numerical display of results of simulation runs in ways that assist in communicating key insights of systems modelling to diverse audiences.

While much of the structure and interactions between model components described below have been informed by best current research evidence and expert knowledge, customisation of models though a participatory modelling approach will draw on the deep tacit knowledge and diverse perspectives of those operating in and interacting with the system being modelled, which is vital to ensuring that the final decision support tool is valid, robust, and fit for purpose.

**Figure 2:**
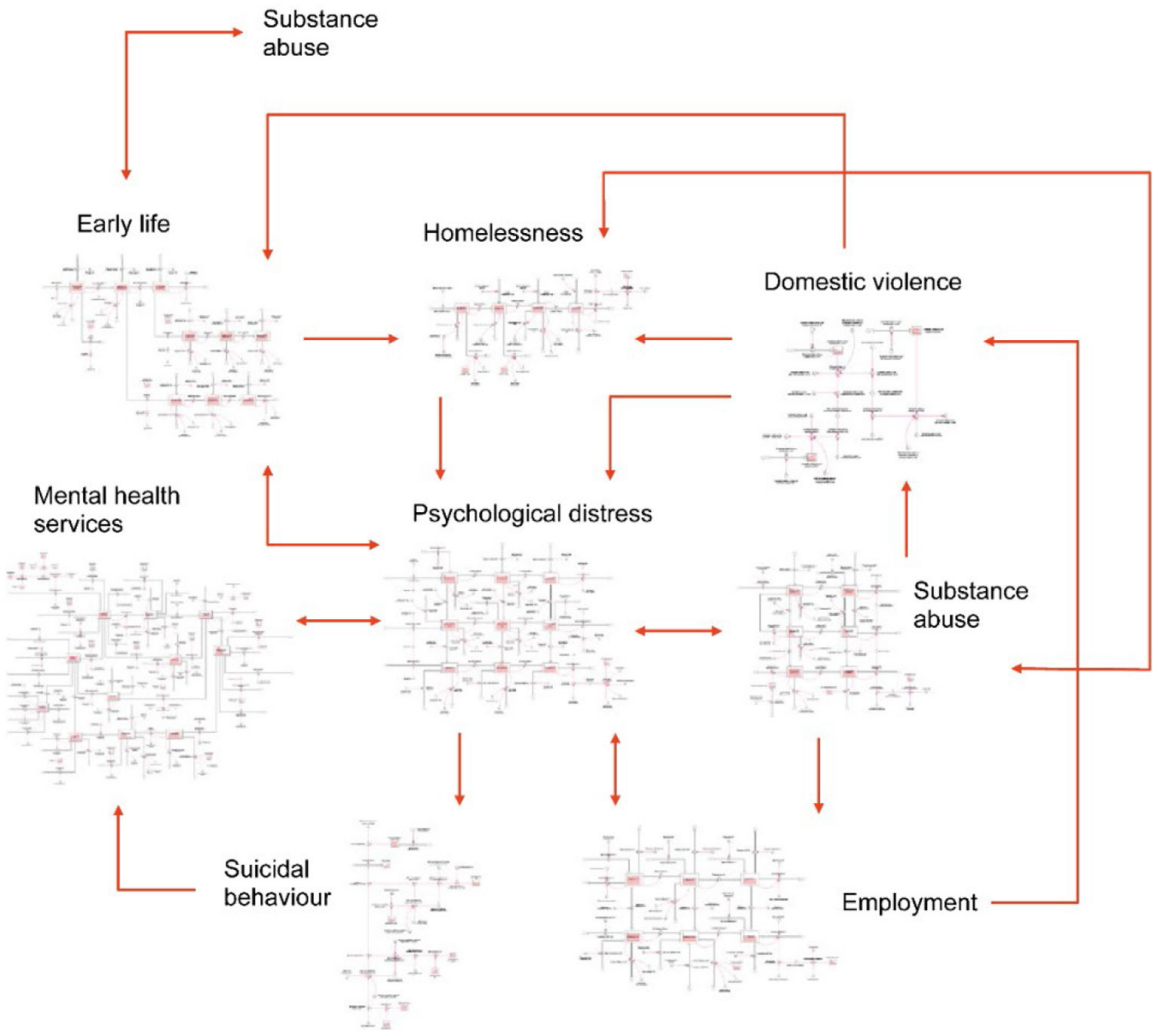
A high-level overview of the causal structure and pathways of one possible model of youth mental health and suicidal behaviour with arrows denoting unidirectional or bidirectional relationships between each component [reprinted with permission (35)].

Figure 2 provides a high-level overview of the causal structure and pathways of previous systems modelling work (33–35, 54) that will be customised for each of the eight sites in the current study. The core structure of each model will include: (1) a **population** component, capturing changes over time in the size and composition of the population resulting from births, migration, ageing, and mortality; (2) a **psychological distress** component that models flows of people between states of low psychological distress (Kessler 10 [K10] score 10–15), moderate psychological distress (K10 score 16–21) and high to very high psychological distress (K10 score 22–50); (3) a **developmental vulnerability** component that captures exposure to childhood adversity and its effect on the risk of developing mental disorder in adolescence and adulthood; (4) an **education** sector that captures participation in education and vocational training; (5) an **employment** sector that captures workforce participation, unemployment, and underemployment; (6) a **mental health services** component that models the movement of psychologically distressed, care seeking people through one of several possible service pathways involving (potentially) general practitioners, psychiatrists and allied mental health professionals (including psychologists and mental health nurses), ED and psychiatric inpatient care, community- and hospital-based outpatient care, and online services; (7) a **suicidal behaviour** component that captures self-harm hospitalisations (used as a proxy for suicide attempts where such data is not captured) and suicide deaths; and (8) a **COVID-19** component that captures the impact of the pandemic and recession on social connectedness, unemployment, and psychological distress from 1 March 2020. Depending on contextual relevance, components capturing **additional key social determinants of mental health** will be integrated, including homelessness, substance abuse, domestic violence, youth detention, and their influence on levels of psychological distress.

While youth (defined as 15–24 years of age) are the primary age group of interest, the models will be stratified by a broader range of age groups (i.e., 0–14, 15–24, 25–44, 45–64, 65+ years) to allow comparative analyses with other age-cohorts. Including the broader range of age groups also allows an exploration of the synergies that can be derived from interventions targeted at multiple points across the mental health service system (beyond youth services) to ensure continuity of care as young people age out of the 15–24-year age category (a current gap and challenge of mental health care in Australia). Models can be stratified for other sociodemographic characteristics e.g., by socioeconomic strata or by Indigenous status, depending on the stakeholder priorities for each site.

The structure and assumptions relating to each component and their interrelationships are detailed in Additional File 1, Sections 1 & 2. The model will capture changes over time (dynamics) within each component and interdependencies between the components of the model, including feedback loops. For example, as the prevalence of psychological distress increases, more people seek care, which stretches service capacity, resulting in increased waiting times, which then increases service disengagement, which in turn increases the duration of psychological distress and the risk of suicide. Similarly, an increase in the unemployment rate, acts directly to increase the incidence of high to very high psychological distress, and domestic violence, which have flow-on effects on rates of substance misuse, adverse early life exposures and homelessness, all of which further increase rates of psychological distress, which in turn influence employment participation and productivity. Such interrelationships and dynamics make it difficult to anticipate (without tools such as systems models) the likely impacts across the system of intervening on one or multiple components of the system.

Primary model outputs will include total (cumulative) numbers of key population mental health indicators including mental-health related ED presentations, psychiatric hospitalisations, self-harm hospitalisations (indicative of suicide attempts) and suicide deaths for the youth population (15–24 years) and total population. The model will also provide estimates of the prevalence of low, moderate, and high to very high psychological distress by age categories (15–24, 25–64, 65 years and above), the prevalence of young people not in employment, education, or training (NEET), and a range of measures of mental health service usage (e.g., mental health-related general practise consultations, psychiatric and allied mental health service consultations, services waiting times, numbers of psychologically distressed consumers that have disengaged from services). Model outputs will typically be calculated every 0.4375 days (i.e., the numerical integration time step, *dt*, one sixteenth of a week) over a period of 30 years, starting from 1 January 2011, permitting comparisons of model outputs with historic data from 2011 to 2020 and forecasts of the impacts of intervention scenarios simulated from the time of implementation (2022) to the start of 2041. This longer-term forecast horizon will importantly encourage transition to a long-term strategic outlook in assessing the value of investment decisions rather than the current short-term perspective that induces more reactive decision making.

The capability of the system dynamics models to enable economic evaluation of intervention combinations will be critical to building regional capability to make compelling investment cases for youth mental health system strengthening and for broader social, education and economic initiatives that will promote life-long mental health and wellbeing. In addition, it will allow analyses of the feedbacks between the economic, social, and mental health sectors, the impacts of broader policy decisions on youth mental health outcomes, and an estimate of the true scale of the cost to regional economies of not mobilising adequate responses to mitigate the impact of the pandemic and recession on young people. Costs of programs, services, policies, initiatives, and outcomes will be integrated into the dynamic model as it is being developed with stakeholders. This enables a highly flexible and pragmatic approach to economic analyses that can take a public finance perspective, a health system perspective, and/or a broader societal perspective, as well as explore the implications of considering alternative time horizons. Local stakeholders will be empowered to use the results of these economic analyses to make compelling moral, social and economic arguments for adequate investments in strengthening regional youth mental health and social systems. A more detailed protocol for the economic evaluation will be provided elsewhere.

### Key Data Requirements for Parameterising and Calibrating the Models

**Figure 3:**
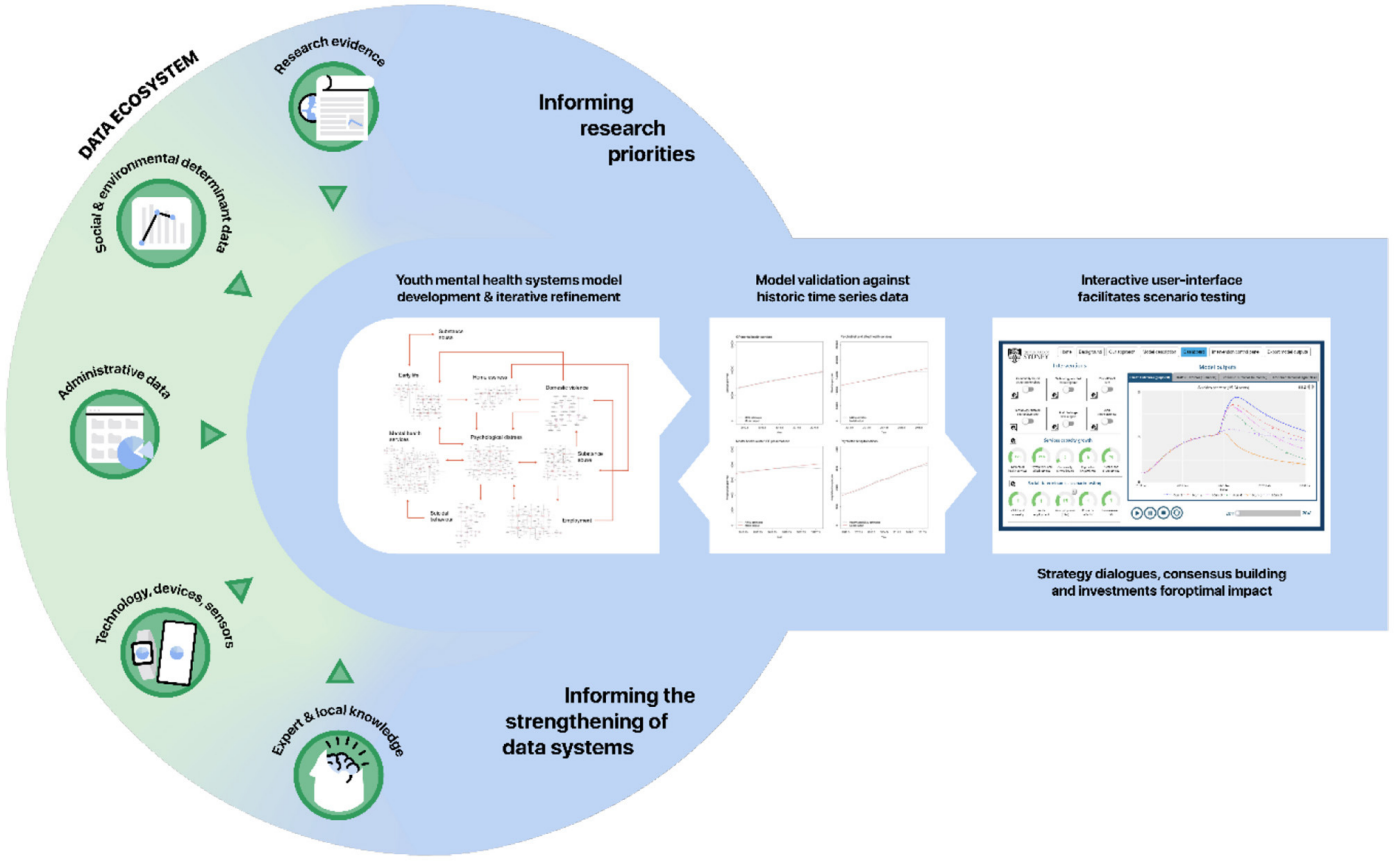
Embedding systems modelling in a continuous system strengthening, monitoring, and evaluation cycle to improve youth mental health.

Unlike early systems modelling of COVID-19 transmission where very little was known about the virus and data was limited, systems modelling in the field of mental health and suicide prevention benefits from decades of quality research, primary and secondary datasets, and multidisciplinary expert and local knowledge. It is acknowledged that the quality and availability of data will vary across regions and across international contexts with data gaps and potential measurement bias present in secondary data sources that will be used to parameterize the models. These challenges should not be considered a deterrent for engaging in systems modelling as several commonly used strategies can be employed to address them. These strategies include the triangulation of multiple data sources, parameter estimation via constrained optimisation, and local stakeholder verification to identify plausible estimates. Systems modelling should be undertaken as a continuous process of hypothesis development, testing and refinement, and embedding these models within a regional data ecosystem supported by ongoing monitoring and evaluation allows them to be updated and refined over time, securing systems models as long-term decision support assets (Figure 3) (55).

A commonly levelled (but misguided) criticism that may prevent systems modelling from being more routinely adopted to inform decision making is that the presence of uncertainty around key model input parameters reduces the value of the model and the credibility of findings. However, a recent systems modelling study exploring the effectiveness of intervention combinations across a wide range of possible estimates of the scale and duration of the adverse COVID-19 effect on psychological distress, found that for this application the best performing suite of interventions for reducing suicide deaths was consistent across the alternative COVID-19 mental health trajectories; highlighting that it is possible for systems modelling to support robust decision making even in the presence of uncertainty (56). In addition, sensitivity analysis can reveal which uncertain model parameters make a significant difference to projected trajectories of key population mental health outcomes, and by doing so elucidate priorities for data collection and further research to advance scientific and public health understanding (57).

Data requirements will depend on the scope of the systems model and outputs of interest which should be determined by national (or local) priorities, stakeholder input, and the research question. Based on previous work, core data requirements for model calibration may include but are not limited to, population estimates and demographic statistics, broad prevalence assessments of mental health symptoms and impairments (e.g., using Kessler 10 scale), labour force statistics, education completion data and estimates of NEET, mental health services data, and suicide and intentional self-harm data (49). A more detailed list of datasets to be used in the current study is presented in Box 1 and provides a guide for applications in other contexts. Model parameterisation additionally draws on research evidence (including systematic reviews, randomised controlled trials, and cohort studies; see Additional File 1), and expert consensus.

Box 1 **Data to be used in the current study.**

Data will be sourced in consultation with stakeholders for each site to ensure that the most relevant and up-to-date information is used. The data sets listed below are for NSW based sites and are included to provide illustrative examples. Equivalent data sources for other sites will be accessed by requesting data from health authorities or from publicly facing health information e.g., www.abs.gov.au; www.aihw.gov.au; https://www.health.qld.gov.au/research-reports/reports/public-health/cho-report/current/data or https://www.health.act.gov.au/about-our-health-system/data-and-publications/healthstats. Where data is unavailable for the region being modelled it will be derived from state- and national-level datasets

1. Population
  *a. Population estimates*. Estimated resident population, disaggregated by age group and sex. Data for the New South Wales models, including population projections (to 2,036) are available from HealthStats NSW (http://www.healthstats.nsw.gov.au/). Historical population data and population projections are also available from the Australian Bureau of Statistics (ABS) (historical estimates are available at: https://www.abs.gov.au/statistics/people/population/national-state-and-territory-population/; projections are available at: https://www.abs.gov.au/statistics/people/population/population-projections-australia/).
  *b. Births per year*. Numbers of births per year. Data for the New South Wales (NSW) models will be derived from HealthStats NSW (http://www.healthstats.nsw.gov.au/). National data are also available from the ABS (https://www.abs.gov.au/statistics/people/population/births-australia/).
  *c. Age-specific mortality*. Numbers of deaths per year (all causes), disaggregated by age group. Mortality rate estimates for the NSW models will be derived from HealthStats NSW (http://www.healthstats.nsw.gov.au/) and are also available at the ABS (https://www.abs.gov.au/statistics/people/population/deaths-australia/).
  *d. Migration*. Numbers of people immigrating and emigrating per year. Data on overseas and interstate arrivals and departures are available from the Australian Bureau of Statistics (https://www.abs.gov.au/statistics/people/population/migration-australia/). The ABS also publishes internal regional migration estimates (https://www.abs.gov.au/statistics/people/population/regional-population/).

2. Psychological distress
  *a. Psychological distress*. Age-specific estimates of the prevalence of moderate to very high psychological distress (Kessler 10 scores 16–50) are available from HealthStats NSW (http://www.healthstats.nsw.gov.au/, NSW models) and the Australian Bureau of Statistics' National Health Survey (https://www.abs.gov.au/statistics/health/health-conditions-and-risks/national-health-survey-first-results, national model). Data for Western Australian models are available by request to the WA Department of Health.

3. Labour force
  *a. Labour force status*. Numbers of working-age people (15 years and above) employed, unemployed, and not in the labour force (NILF), disaggregated by age group. Data will be derived from the Australian Bureau of Statistics (https://www.abs.gov.au/statistics/labour/employment-and-unemployment/labour-force-australia/).
  *b. Underemployment*. Number of working-age people (15 years and above) underemployed, disaggregated by age group. Data are available from the Australian Bureau of Statistics (https://www.abs.gov.au/statistics/labour/employment-and-unemployment/labour-force-australia/).
  *c. Changes in labour force status*. Data on numbers of people aged 15 years and above changing labour force status per month (i.e., net monthly flows between labour force states) are available from the ABS (https://www.abs.gov.au/statistics/labour/employment-and-unemployment/labour-force-australia/).

4. Education
  *a. Persons with a non-school qualification*. Numbers of people with a non-school qualification (at certificate III level or above), disaggregated by age group. Data are available from the Australian Bureau of Statistics (https://www.abs.gov.au/statistics/people/education/education-and-work-australia).
  *b. Current post-secondary study*. Numbers of people studying for a non-school qualification (certificate III level or above), disaggregated by age group. Data are available from the Australian Bureau of Statistics (https://www.abs.gov.au/statistics/people/education/education-and-work-australia).
  *c. Post-secondary study completion*. Numbers of students completing post-secondary study (certificate III level or above) per year, disaggregated by age group. Data are available from the Australian Bureau of Statistics (https://www.abs.gov.au/statistics/people/education/education-and-work-australia).
  *d. Young people not fully engaged in employment or education*. Data on numbers of young people (15–24 years) not fully engaged in employment or education are available from the Australian Bureau of Statistics (https://www.abs.gov.au/statistics/people/education/education-and-work-australia).

5. Developmental vulnerability
  *a. Developmental vulnerability*. Data on the proportions of children entering school (aged *c*. 5 years) who are developmentally vulnerable (on 2 or more domains) are from the Australian Early Child Development Census (https://www.aedc.gov.au/data/data-explorer).

6. Substance use disorders
  *a. Prevalence of substance use disorders*. Age-specific prevalence estimates for NSW models will be derived from NSW Population Health Survey data on adult lifetime risky drinking (obtained from HealthStats NSW: http://www.healthstats.nsw.gov.au/) and data on high risk drinking from the National Health Survey (https://www.abs.gov.au/statistics/health/health-conditions-and-risks/national-health-survey-first-results).
  *b. Alcohol and other drug treatment services*. Numbers of closed treatment episodes per year, disaggregated by age group. Data are available from the Australian Institute of Health and Welfare (https://www.aihw.gov.au/reports/alcohol-other-drug-treatment-services/aodts-phn/data).

7. Mental health services
  *a. Medicare-subsidised mental health services*. Numbers of mental health-related general practitioner services, psychiatrist services, and psychologist and other allied health services provided per year, disaggregated by age group. Data are available from the Australian Government Department of Health (https://www1.health.gov.au/internet/main/publishing.nsf/Content/PHN-Mental_Health_Data, PHN level) and the Australian Institute of Health and Welfare (https://www.aihw.gov.au/reports/mental-health-services/mental-health-services-in-australia/data, national and state-level).
  *b. Community mental health care (CMHC) services*. Numbers of state funded CMHC services provided per year, disaggregated by age group. National and regional level data are available from the Australian Institute of Health and Welfare (https://www.aihw.gov.au/reports/mental-health-services/mental-health-services-in-australia/data).
  *c. Mental health-related emergency department presentations*. Numbers of mental health-related emergency department presentations per year, disaggregated by age group. National and regional level data are available from the Australian Institute of Health and Welfare (https://www.aihw.gov.au/reports/mental-health-services/mental-health-services-in-australia/data).
  *d. Admitted patient care*. Numbers of public hospital admissions for mental disorders (total, including hospitalisations not involving specialised psychiatric care) and numbers of hospitalisations for mental disorders involving specialised psychiatric care per year, disaggregated by age group. Data are derived from HealthStats NSW (http://www.healthstats.nsw.gov.au/, NSW models only) and the Australian Institute of Health and Welfare (https://www.aihw.gov.au/reports/mental-health-services/mental-health-services-in-australia/data, national and regional models).

8. Suicidal behaviour
  *a. Self-harm hospitalisations*. Numbers of hospitalisations for intentional self-harm per year (as a proxy for suicide attempts), disaggregated by age group. Data for the NSW models are available from HealthStats NSW (http://www.healthstats.nsw.gov.au/). National and state-level data are available from the Australian Institute of Health and Welfare (https://www.aihw.gov.au/suicide-self-harm-monitoring/data/data-downloads).
  *b. Suicides*. Numbers of suicides per year, disaggregated by age group. Data for the NSW models are available from HealthStats NSW (http://www.healthstats.nsw.gov.au/). National-level data are available from the ABS (https://www.abs.gov.au/statistics/health/causes-death/causes-death-australia).

Parameter values that cannot be derived directly from available data or published research will be estimated via constrained optimisation, using historical time series data on a wide range of mental health and social outcomes, including psychological distress prevalence, self-harm hospitalisation and suicide rates, rates of mental health services usage (general practise consultations, specialised psychiatric services, ED and hospital inpatient care, community-based mental health services), and unemployment and labour force participation rates. Powell's method (58) will be employed to obtain the set of (optimal) parameter values minimising the sum of the mean absolute percent error calculated for each time series separately (i.e., the mean of the absolute differences between the observed time series values and the corresponding model outputs, where each difference is expressed as a percentage of the observed value). Powell's method (using the BOBYQA algorithm) is well-established and performs well for optimisation problems involving a larger number of parameters (30+) where the performance of other methods drops significantly (59).

**Figure 4:**
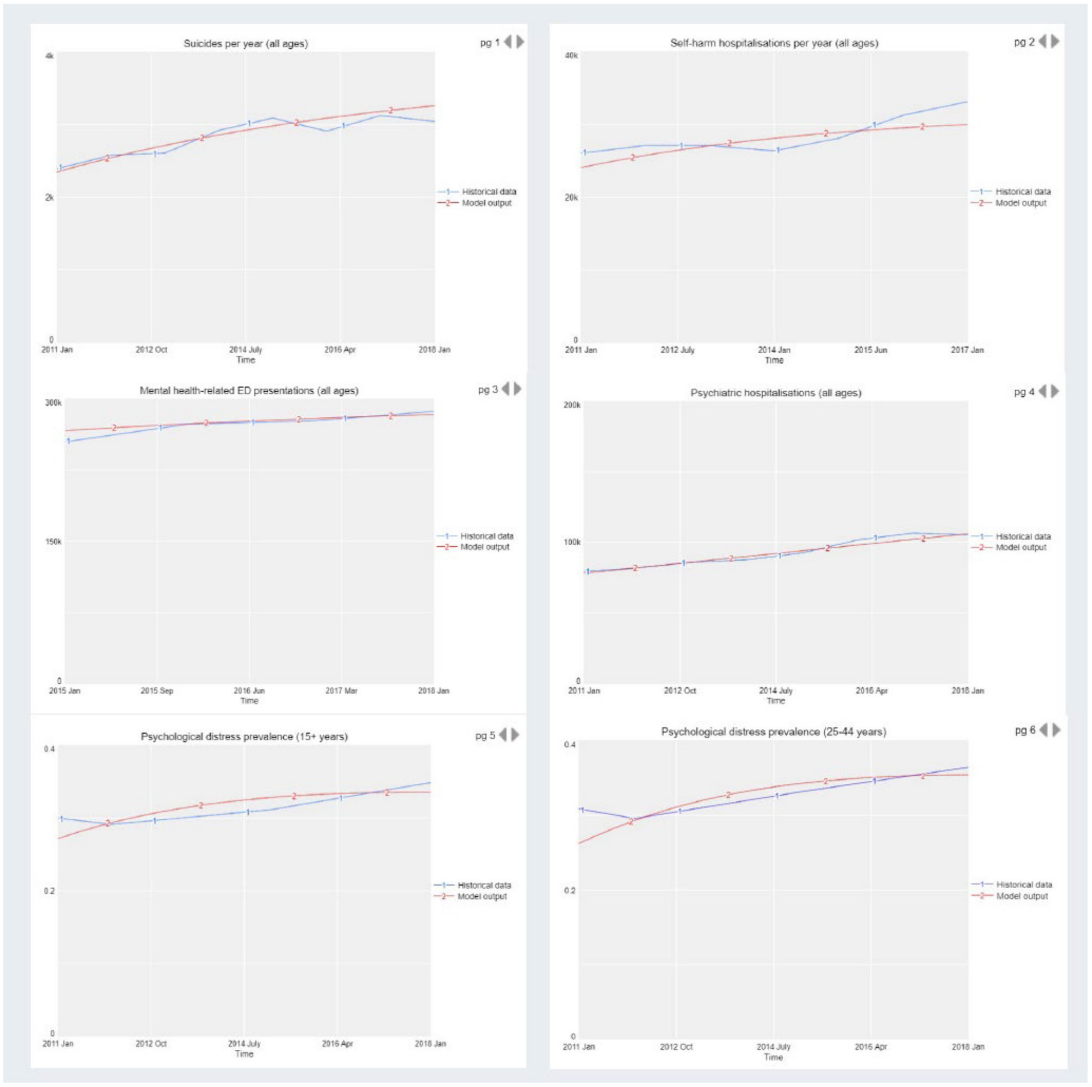
Model validation by comparison of model output with historic time series data across a range of outcome indicators.

Systems models represent a causal hypothesis of the structure and behaviour of a given system, and hence need to be tested and validated. Validation is achieved by comparing model output with historic time series data across a range of outcome indicators. Figure 4 provides an example from previous work (33) where the blue lines represent real world time series data from 2011 to 2017/18 and the red lines are the outputs of the model. When systems models broadly reproduce real world data patterns from the past, it affords confidence in the validity of the underlying causal mechanism driving forward projections. In addition, good systems modelling practise recommends ensuring face validity of the structure, performance, and key insights of the model among diverse system actors through the participatory model building process (60).

### Policy Testing and Sensitivity Analyses

We will model the potential impacts on key youth mental health outcomes of a set of interventions identified by the key stakeholder group at each site. A range of programs, services, initiatives, and scenarios previously modelled are provided as examples in Additional File 1, Section 3. Intervention scenarios will be compared against a baseline (business as usual), in which existing policies and programs remain in place until the end of the simulation. Sensitivity analyses will be performed to assess the impact of uncertainty in estimates of the direct effects of each intervention, and forecasted growth in services capacity (i.e., GP mental health services, psychiatrists and allied services, community mental health services, psychiatric hospital care, and alcohol and drug services) on the simulation results. We will typically use Latin hypercube sampling to draw 100 sets of values for the selected model parameters from a uniform joint distribution spanning a broad sample space of ± 20% of the default values.

To answer research question (i), differences in projected (cumulative) numbers of key mental health outcomes such as self-harm hospitalisations and suicide deaths between a non-COVID-19 baseline (i.e., had the pandemic not occurred) and COVID-19 baseline scenario will be calculated for each set of parameter values, summarised using simple descriptive statistics, and compared across the eight sites to determine whether pre-pandemic regional variations in youth metal health outcomes are likely to be exacerbated as a result of COVID-19. To answer research question (ii), at each site, differences in projected (cumulative) numbers of key mental health outcomes such as self-harm hospitalisations and suicide deaths between the baseline and intervention scenarios will be calculated for each set of parameter values and summarised using simple descriptive statistics. Additional key interventions will be integrated into the models at some sites to ensure all eight models have a common set of interventions to address research question (iii). The extent to which the best performing intervention combinations are consistent across diverse contexts will be assessed by undertaking a series of single-objective optimisations identifying best-performing combinations of youth mental health and suicide prevention intervention combinations for each of the eight sites. This will determine the extent to which intervention strategies to improve youth mental health and prevent suicide are generalisable across diverse regions of Australia. Similar international analyses may reveal a set of strategies that offer promise in the global youth mental health response. All analyses will be conducted over the simulation period 2022–2041. In addition, further testing of each model will deliver a series of qualitative insights which may include, for example, why particular evidenced based interventions fail to deliver impact in different contexts [or result in unintended consequences (52)], and the identification of leverage points in the system where targeted intervention may deliver greater than anticipated effects.

## Discussion

Current global challenges including climate change, civil strife, a pandemic, and the deepest global recession since the Second World War are generating extensive social disruption and uncertainty that have the potential to undermine the mental health, wellbeing, and futures of young people. The scale and complexity of the challenges requires new thinking and analytic tools that can capture the dynamics and interrelationships of physical, social, economic, and health systems, and support national and regional decisions to deliver more strategic, proactive, and effective responses (49, 61). Systems modelling has long been successfully applied in many fields of science and in business, and was used to inform effective, proactive responses to the COVID-19 pandemic in many countries (62–65). This research seeks to establish regional decision support infrastructure to empower communities to effectively allocate limited resources in ways that will improve youth mental health, and to make compelling investment cases to funders and policy makers to attract the broader social and economic supports needed for young people to flourish. Specifically, this study will leverage a transparent, inclusive, systems modelling approach (informed by best available research evidence, a range of national, state, and regional datasets, as well as expert and local knowledge) not only to inform effective responses to the potential adverse impacts of COVID-19 on youth mental health in the regions in which it is applied, but to move towards an understanding of the extent to which insights may be relevant to the global response. While the focus of the current study is on youth mental health, the model's inclusion of mental health outcomes across all ages allows extended analysis of youth specific vs. universal investments in mental health in each region. This will allow regional decision makers and community stakeholders to consider the philosophical implications of focussing investments in youth mental health.

Computer simulation is more than a tool; it is known as the third pillar of science (after theory and experiment) (66). Simulation allows researchers and decision makers to move beyond what can be manipulated within the scale, time, and ethical limits of the experimental approach (61). Systems modelling and simulation provides immediate outcome feedback for a range of decision options under different conditions, allows the testing of extremes and alternative assumptions, and often elicits in a short period the learning of a lifetime of implementation, evaluation, experimentation, and experience (61). Such learning when achieved collectively, has the potential to enhance regional self-determination, help us move beyond incremental adjustments to the status quo, and catalyse collaborative, cooperative, and effective transformational change. By making available a systems modelling blueprint based on years of applied research in mental health service planning and suicide prevention, we provide not only a springboard for more timely construction and deployment of systems models to inform responses to the mental health sequelae of the COVID-19 pandemic, but encourage researchers to use, challenge, and advance the existing work for scientific and societal progress.

## Ethics Statement

This study has been approved by the Human Research Ethics Committee of the Sydney Local Health District (Protocol No X21-0151 & 2021/ETH00553).

## Author Contributions

J-AO and AS: manuscript concept and drafting. All authors conducted critical revision of the manuscript and provided important intellectual content. All authors read and approved the final manuscript.

## Funding

This research is being conducted under the Brain and Mind Centres' ‘Right care, first time, where you live' Program, enabled by a AUD12.8 million partnership with BHP Foundation. The program will develop decision support infrastructure to guide investments and actions to foster the mental health and wellbeing of young people in their communities.

## Conflict of Interest

J-AO is both Head of Systems Modelling, Simulation & Data Science at the University of Sydney's Brain and Mind Centre and Managing Director of Computer Simulation & Advanced Research Technologies (CSART). IH is the Co-Director, Health and Policy at the Brain and Mind Centre (BMC) University of Sydney. The BMC operates an early-intervention youth services at Camperdown under contract to headspace. He is the Chief Scientific Advisor to, and a 5% equity shareholder in, InnoWell Pty Ltd. InnoWell was formed by the University of Sydney (45% equity) and PwC (Australia; 45% equity) to deliver the $30 M Australian Government-funded Project Synergy (2017–20; a three-year program for the transformation of mental health services) and to lead transformation of mental health services internationally through the use of innovative technologies. LF is Senior Research Fellow with the Brain and Mind Centre, University of Sydney; Director of Knowledge Translation and Health Outcomes, Epidemiology Section, ACT Health; & Director of Policy Applications & Translational Science of CSART. The remaining authors declare that this work was conducted in the absence of any commercial or financial relationships that could be construed as a potential conflict of interest.

## Publisher's Note

All claims expressed in this article are solely those of the authors and do not necessarily represent those of their affiliated organizations, or those of the publisher, the editors and the reviewers. Any product that may be evaluated in this article, or claim that may be made by its manufacturer, is not guaranteed or endorsed by the publisher.

## Supplementary Material

Additional File 1 Detailed model structure and assumptions underpinning a system dynamics model of the social and system determinants of mental health and suicidal behaviour to be implemented and adapted across diverse contexts.
