# Supplementary material for: Which Social, Economic, and Health Sector Strategies Will Deliver the Greatest Impacts for Youth Mental Health and Suicide Prevention? Protocol for an Advanced, Systems Modelling Approach
Source: Front Psychiatry. 2021 Oct 13;12:759343. doi: 10.3389/fpsyt.2021.759343 (PMC8548722; doi:10.3389/fpsyt.2021.759343)
Supplement: Additional File 1 — Detailed model structure and assumptions underpinning a system dynamics model of the social and system determinants of mental health and suicidal behaviour to be implemented and adapted across diverse contexts. [file Data_Sheet_1.pdf]

Additional file 1

Section 1. Detailed modules to support the development and adaption of a system dynamics model for suicide prevention and mental health services planning.

Much of the model structure outlined below has been implemented and validated at national, state, and regional levels in Australia and is provided in modular form (model components, or sectors) allowing systems models to be constructed and customized for other national and international applications. To demonstrate the validity of the structure of each component in the contexts in which these model components have been applied, example comparisons are presented between real world data and outputs generated by the model. This does not, however, negate the potential customisation needed for applications in diverse contexts.

1.1. Population sector

Figure S1 shows the structure of the population sector, which models changes in the size and composition of the Australian population resulting from births, migration, aging, and mortality. The total national population is represented as 5 stocks (i.e., state variables), corresponding to numbers of people aged 0−14 years, 15−24 years, 25−44 years, 45−64 years, and 65 years and above. Population size increases via births (which flow into the stock of 0−14-year-olds) and immigration and decreases through emigration and mortality. Aging is modelled as a first-order delay, in which people flow out of each stock (except the stock of people aged ≥ 65 years) at a rate n/d, where n is the number of people in the stock at any particular time point and the delay time d is the mean number of years a person spends in the stock. Births and deaths occur at rates bP and $\theta_i m P_i$, respectively, where P is the total population, θ_i and P_i are, respectively, the mortality hazard ratio and population for age group i, and the per capita birth rate b and per capita mortality rate for the total population m decline at constant fractional rates per year. Net migration for age group i is equal to $I_i - e_i P_i$, where I_i is age-specific immigration per year and e_i is the age-specific per capita emigration rate per year. Population estimates derived from the system dynamics model are presented together with estimates from the Australian Bureau of Statistics (2019) in figure S2.

1.2. Psychological distress sector

The psychological distress sector captures transitions between states of low psychological distress (Kessler 10 [K10] scores 10−15) and moderate to very high psychological distress (K10 scores ≥ 16) in each age group (figure S3). Numbers of people currently experiencing moderate to very high levels of psychological distress are modelled as stocks with inflows corresponding to psychological distress incidence and outflows corresponding to recovery. Psychological distress incidence is equal to hsL, where s is the reference (or base) per capita rate of distress onset per year, h is the product of the effects of developmental vulnerability during childhood (Green et al., 2019), unemployment or (for people aged 15−24 years) non-participation in education or employment (Australian Bureau of Statistics, 2012a), underemployment (Dooley et al., 2000), and other modelled risk factors on psychological distress onset, and L is the number of people experiencing low levels of psychological distress. Moderately to highly distressed people in each age group recover at a yearly rate $rH + T$, where r is the per capita spontaneous recovery rate per year, H is the number of people currently experiencing moderate to very high psychological distress, and T is the number of people moving from a state of moderate to very high psychological distress to a state of low psychological distress per year due to effective mental health treatment (see section 1.8 below). Aging of people experiencing moderate to very high levels of distress is modelled using the same approach described for the population sector above (i.e., as a first-order delay; see section 1.1).

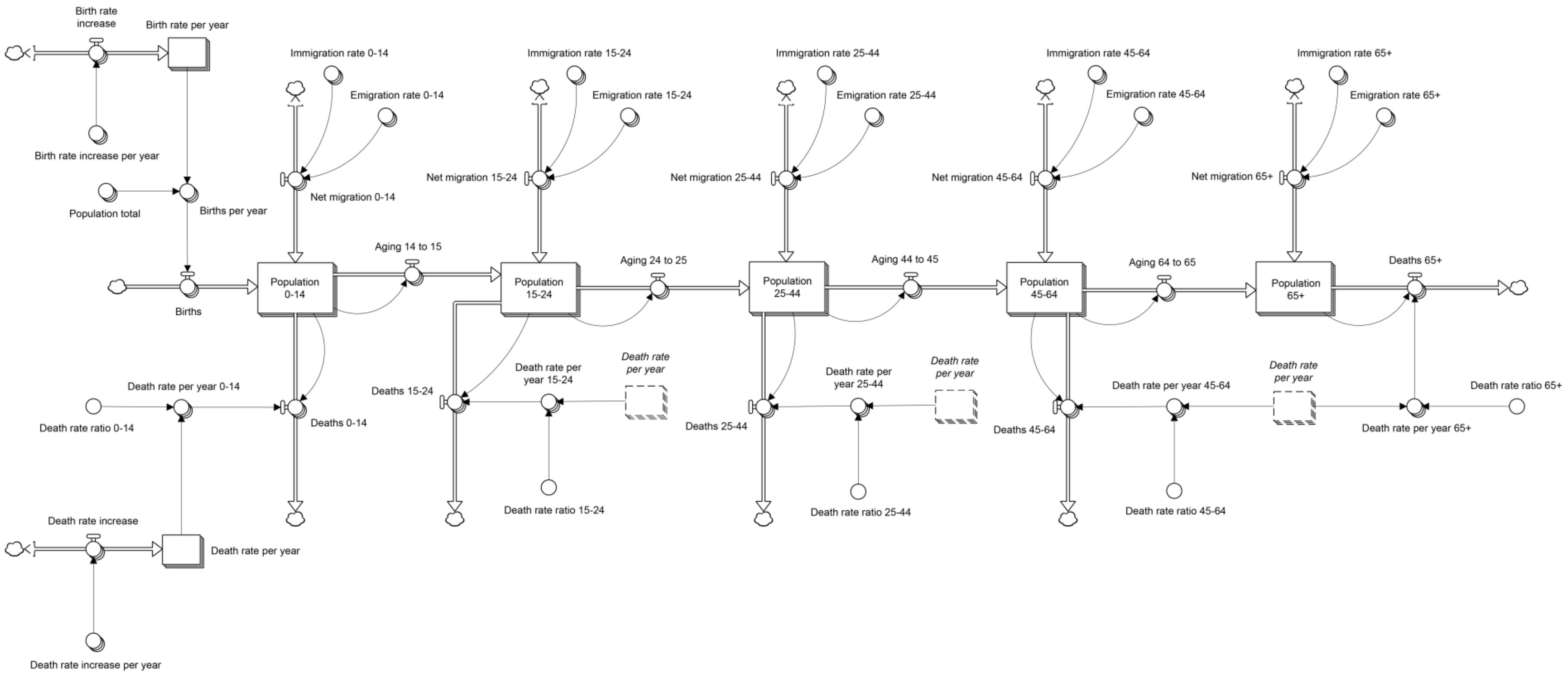

Figure S1. Structure of the population sector.

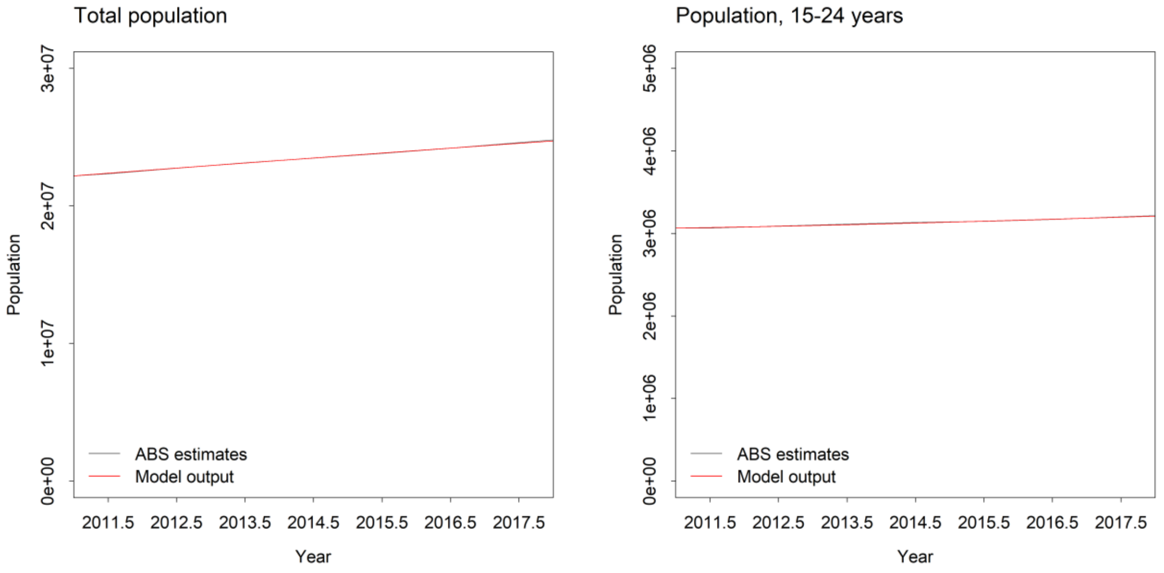

Figure S2. Population estimates (all ages and 15–24-year-olds) derived from the system dynamics model and from the Australian Bureau of Statistics (ABS; 2019a).

Numbers of people with moderate to very high psychological distress increase (or decrease) via net migration at age-specific rates $p_i I_i - q_i e_i P_i$, where p_i and q_i are the age-specific proportions of people with moderate to very high psychological distress among overseas arrivals and Australian residents, respectively, I_i is total age-specific immigration per year, e_i is the age-specific per capita emigration rate per year, and P_i is the number of people in age group i in the Australian population. Age-specific per capita mortality among people with moderate to very high psychological distress is assumed to be 1.37 times that for people with low psychological distress (Russ et al., 2012). Moderate to very high psychological distress prevalence estimates derived from the simulation model and from the National Health Survey (Australian Bureau of Statistics, 2018b) are presented in figure S4.

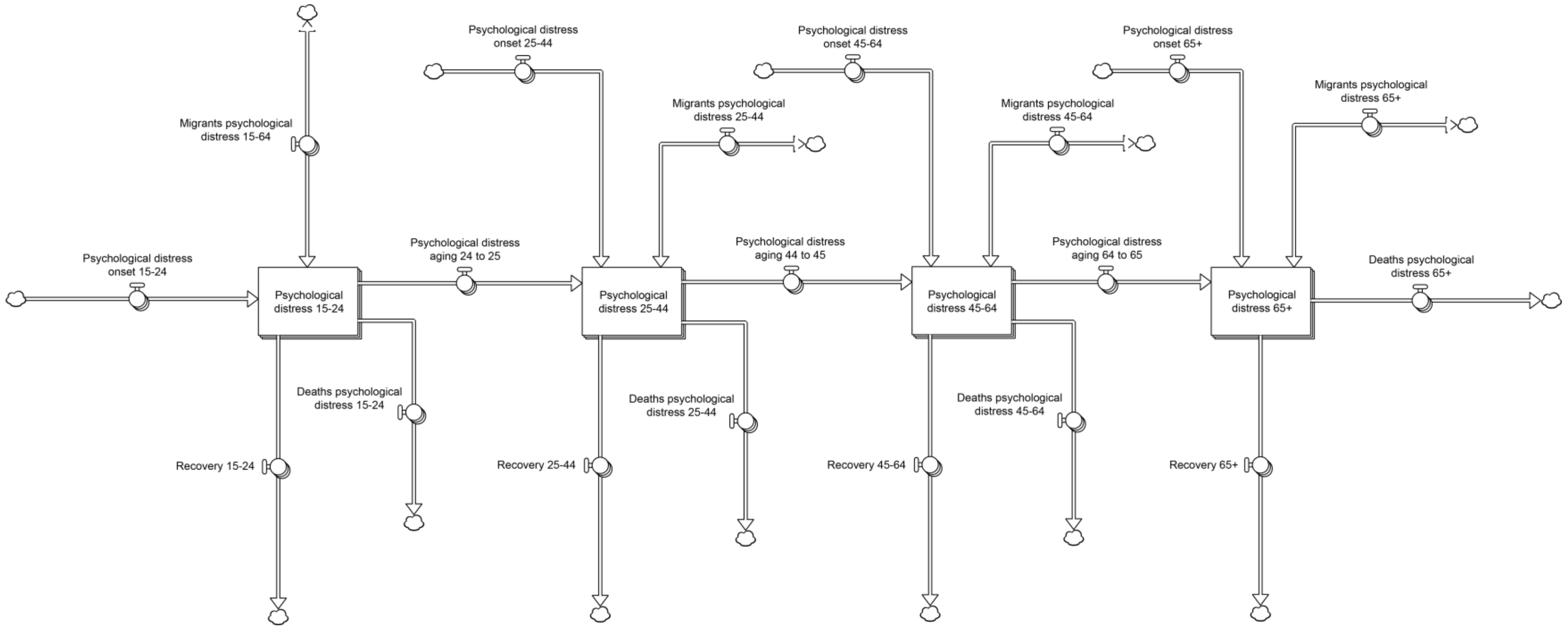

Figure S3. Stock and flow structure of the psychological distress sector.

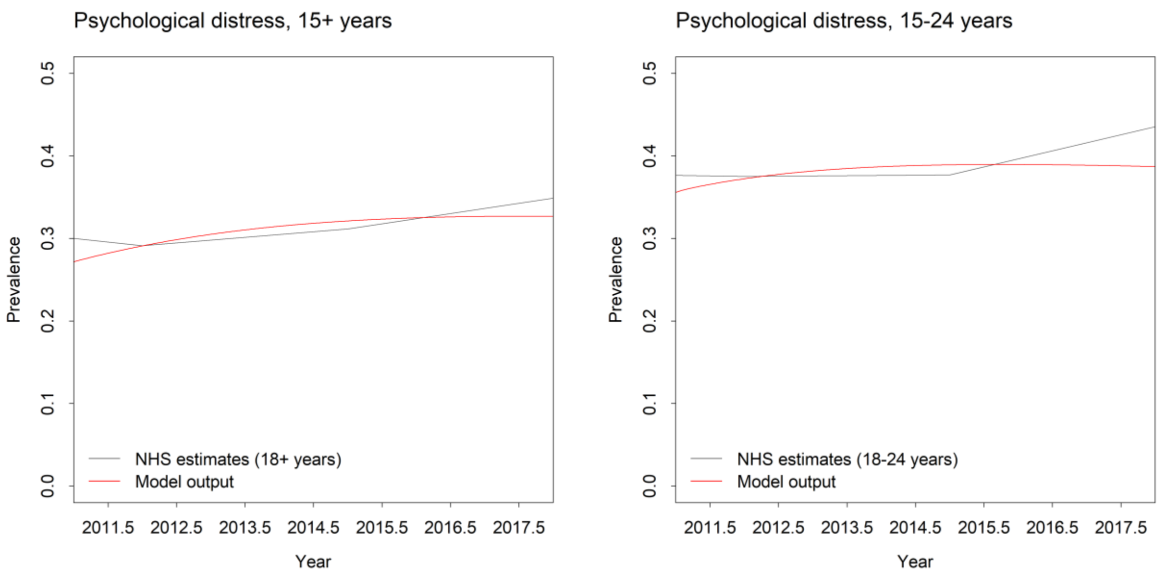

Figure S4. Moderate to very high psychological distress prevalence estimates derived from the dynamic model and from the National Health Survey (NHS; Australian Bureau of Statistics, 2018b).

1.3. Developmental vulnerability sector

The developmental vulnerability sector models exposure to adversity during childhood and its effect on the risk of developing mental disorders in adolescence and adulthood. The number of developmentally vulnerable children aged 0−14 years is modelled as a stock that increases as children at low risk of psychopathology transition to a state of higher risk (see figure S5); we assume that the onset of psychopathological vulnerability depends on cumulative exposure to adverse experiences (e.g., parental psychological distress, physical and sexual abuse, domestic violence, poverty) and is irreversible. The incidence of significant psychopathological vulnerability is equal to hsR, where s is the reference per capita rate at which children at low risk of psychopathology transition to a state of higher risk per year, h is the product of the effects of parental psychological distress and other modelled risk factors on the risk of developing mental disorders in later life (Dean et al., 2018), and R is the number of low-risk 0−14-year-olds in the population. The per capita rate s was set so that the prevalence of psychopathological vulnerability (i.e., for 0−14-year-olds) aligned with estimates of the proportion of developmentally vulnerable children derived from the Australian Early Child Development Census (https://www.aedc.gov.au; figure S6).

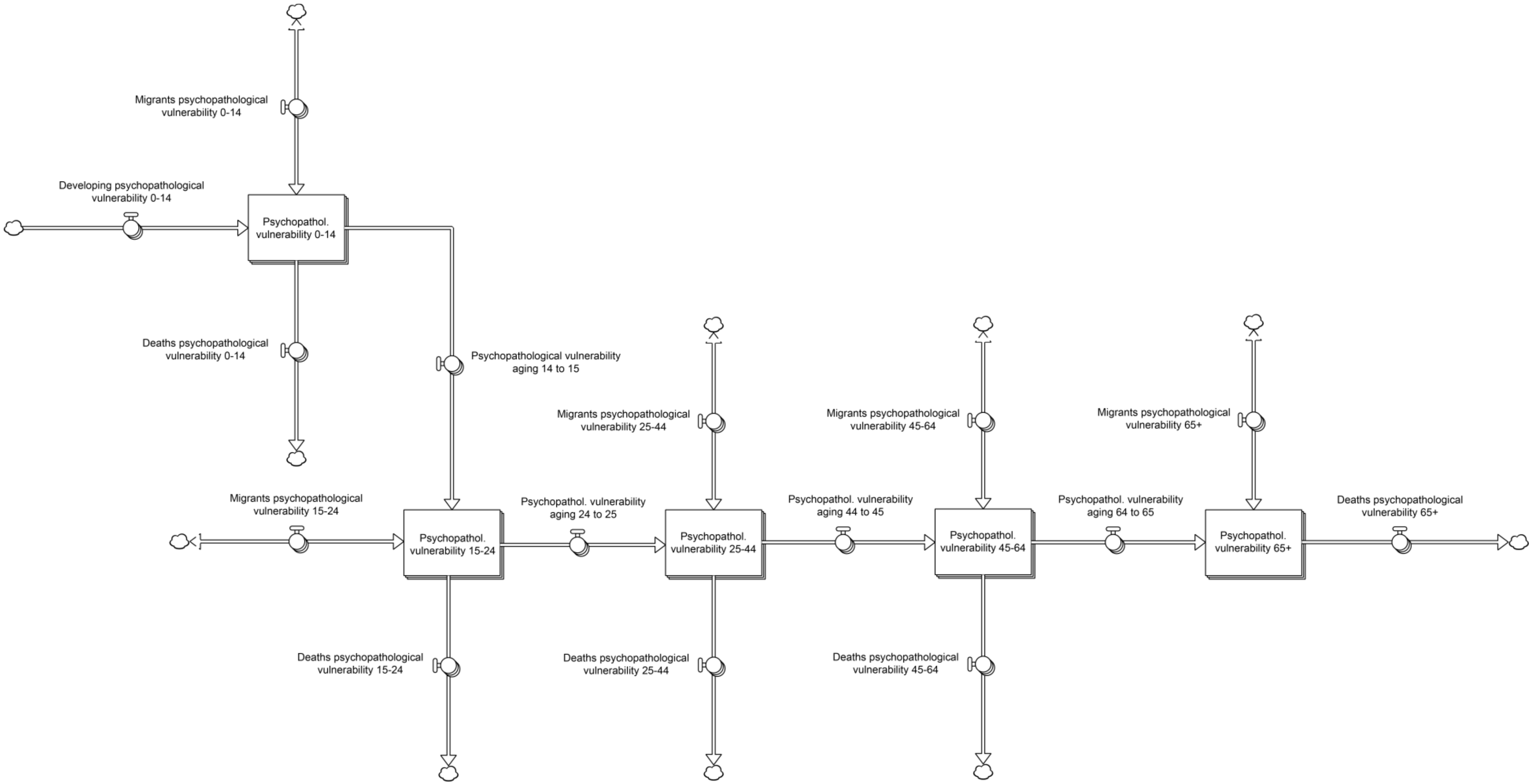

Figure S5. Stock and flow structure of the developmental vulnerability sector.

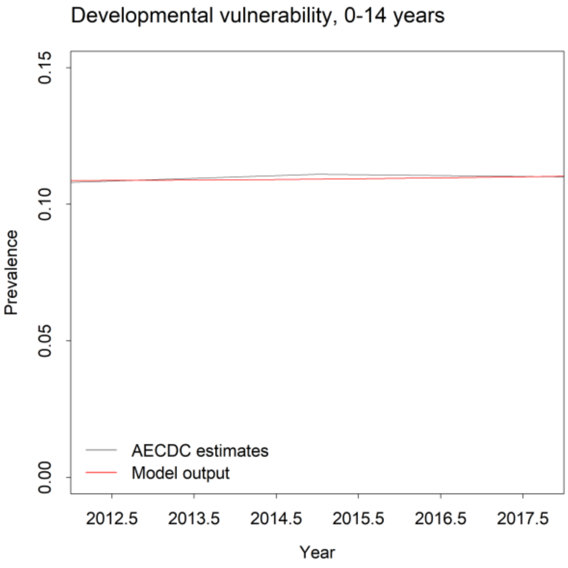

Figure S6. Psychopathological vulnerability prevalence estimates derived from the system dynamics model and from the Australian Early Child Development Census (AECDC; https://www.aedc.gov.au).

As developmentally vulnerable children turn 15, they flow into an aging chain that provides a means of tracking numbers of people aged 15−24 years, 25−44 years, 45−64 years, and 65 years and above at higher risk of developing mental disorders due to adverse exposures in childhood. A person's level of risk (either high or low) at the time they reach 15 years of age is assumed to remain unchanged (people are removed from the aging chain only through mortality and emigration; see figure S5) and affects the probability that they will experience moderate to very high psychological distress throughout their life (see section 1.2). Age-specific per capita mortality rates for people with a high risk of developing mental disorders are assumed to be 1.37 times those for people with a low level of risk (Russ et al., 2012). Net migration adds to (or subtracts from) the numbers of people at increased risk of moderate to very high psychological distress at age-specific rates $p_i I_i - q_i e_i P_i$, where p_i and q_i are the age-specific proportions of people with a higher risk of psychopathology among overseas arrivals and Australian residents, respectively, I_i is total age-specific immigration per year, e_i is the age-specific per capita emigration rate per year, and P_i is the number of people in age group i in the Australian population.

1.4. Education and training sector

Figure S7 shows the structure of the education and training sector, which captures post-secondary education and vocational training enrolment and completion rates (certificate III level and above; Australian Bureau of Statistics, 2020) among people aged 15−64 years. Numbers of people currently studying for a post-secondary

qualification are modelled as stocks with inflows corresponding to enrolment and outflows corresponding to completion and discontinuation (i.e., dropping out of study prior to completion). Age-specific enrolment rates are calculated as gkN, where k is the reference per capita enrolment rate per year, g is the effect of psychological distress on entry into post-secondary study (Lee et al., 2009), and N is the number of people not currently studying. Completion and discontinuation rates are equal to cS and hdS, respectively, where c is the per capita completion rate per year, d is the base per capita discontinuation rate per year, h is the effect of psychological distress on the discontinuation rate (Lee et al., 2009), and S is the number of people currently studying for a post-secondary qualification. People undertaking post-secondary study are assumed to experience the same per capita mortality as people not currently studying (i.e., the per capita mortality rates for the total population are applied to the stocks of people enrolled in post-secondary education and vocational training).

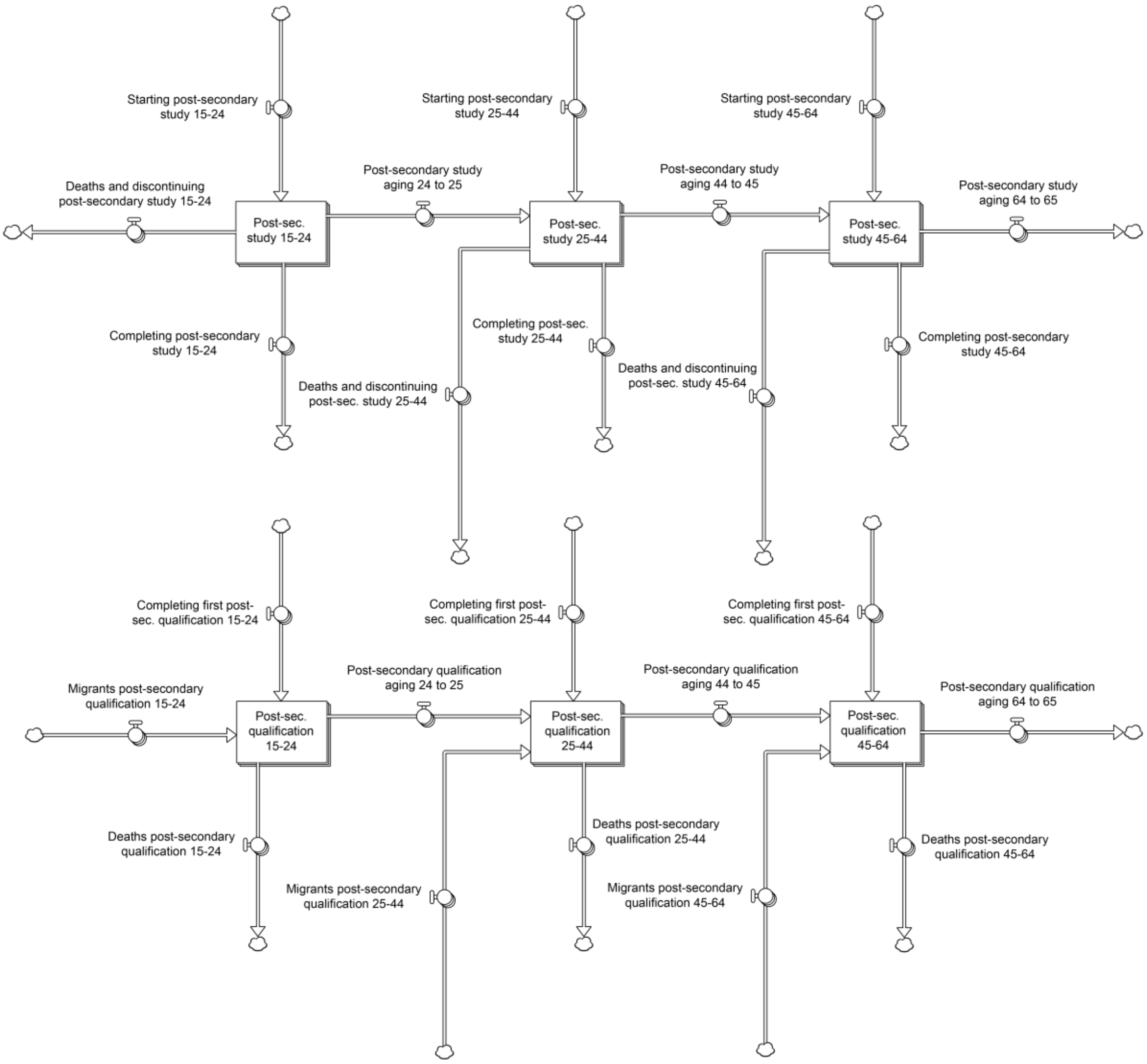

Figure S7. Stock and flow structure of the education sector.

As people complete study, a fraction f, corresponding to those without a previous post-secondary qualification, flow into stocks of people aged 15−24 years, 25−44 years, and 45−64 years with at least one qualification. Numbers of people with a post-secondary qualification increase (or decrease) due to net migration at rates equal to $p_i I_i - q_i e_i P_i$, where p_i and q_i are the age-specific proportions of people with a post-secondary qualification among overseas arrivals and Australian residents, respectively, I_i is total age-specific immigration per year, e_i is the age-specific per capita emigration rate per year, and P_i is the number of people in age group i in the Australian population. Per capita mortality rates for people with a post-secondary qualification are assumed to be 0.74 times those for people without a qualification (Backlund et al., 1999). Figure S8 presents model-based estimates of the numbers of people enrolled in post-secondary education and vocational training and the proportions of people with a post-secondary qualification, together with estimates derived from the Australian Bureau of Statistics (2020).

1.5. Employment sector

The structure of the employment sector, which models labour market transitions in the working-age population (15−64 years), is presented in figure S9. The total labour force is represented as nine stocks, corresponding to the numbers of fully employed, underemployed, and unemployed people aged 15−24 years, 25−44 years, and 45−64 years. Adolescents turning 15 are assumed to enter the population of those not in the labour force (NILF), i.e., people who are neither employed nor seeking employment (15-years-olds are required to attend school full-time in Australia, and the vast majority will not be in the labour force). People not in the labour force may decide to seek employment, at which point they enter the stocks of unemployed people, while those seeking employment (i.e., the unemployed) may leave the labour force. Age-specific net flows from the unemployed population to the population of people not in the labour force are calculated as $fsU - hrN$, where U and N are, respectively, the numbers of unemployed people and people not in the labour force, s is the reference (or base) per capita rate that unemployed people leave the labour force per year, f is the effect of the unemployment rate on labour force participation (assumed to be greater than 1, so that increases in the unemployment rate reduce participation; Mitchell et al., 2019), r is the base per capita rate that people enter the labour force per year, and h is the product of the effects of psychological distress (Frijters et al., 2014), completion of post-secondary education or vocational training (Australian Bureau of Statistics, 2020), and other modelled risk (or protective) factors on the labour force entry rate.

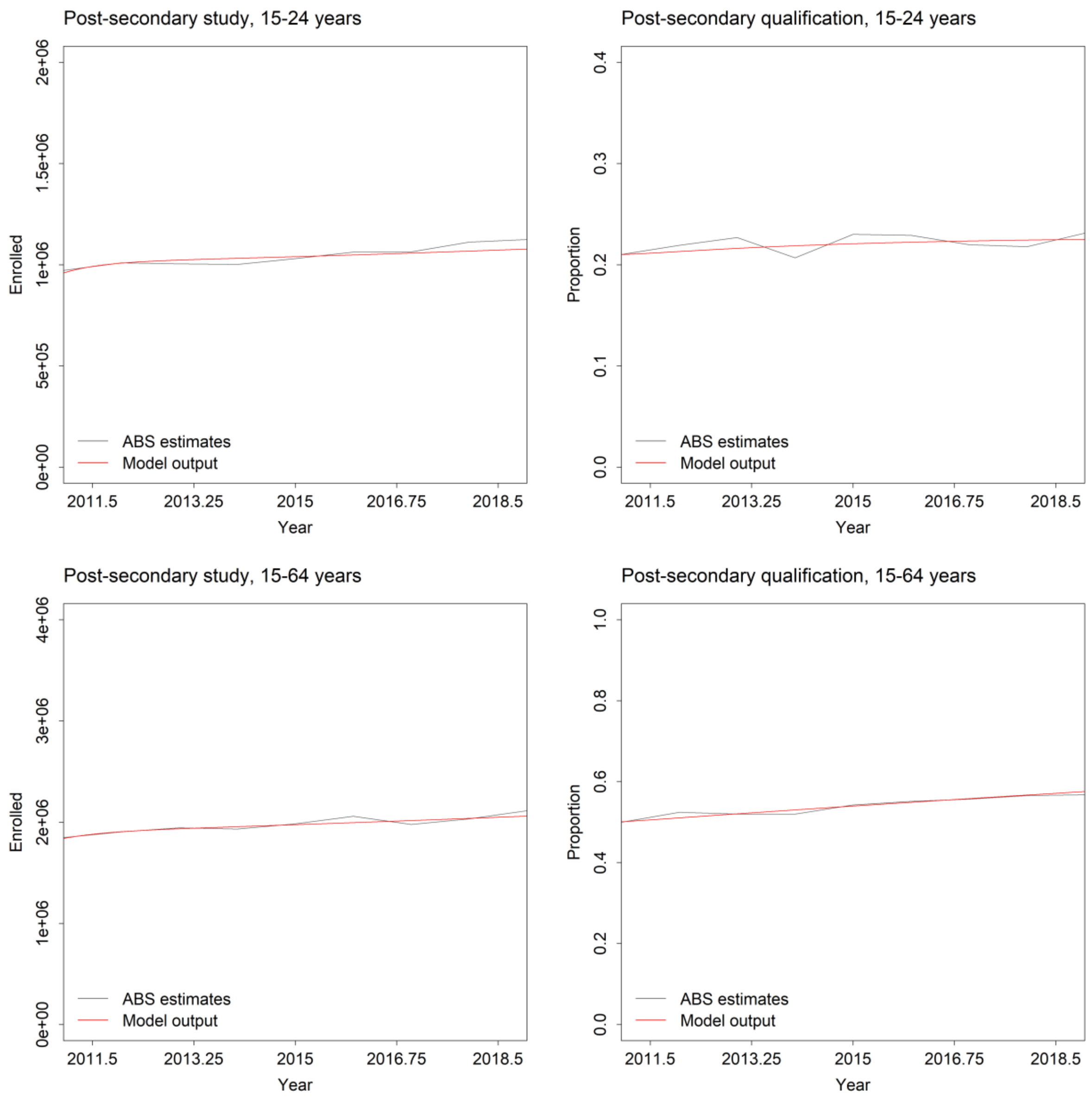

Figure S8. Estimates of numbers of people enrolled in post-secondary education and vocational training and the proportions of people with a post-secondary qualification derived from the system dynamics model and from the Australian Bureau of Statistics (ABS; 2020).

Fully employed people become unemployed at age-specific rates vE, where E is the fully employed population and v is the per capita rate of job loss per year, while unemployed people secure full employment at age-specific rates bwU, where U is the unemployed population, w is the reference (or base) per capita rate of full employment initiation per year, and b is the product of the effects of psychological distress (Frijters et al., 2014), completion of post-secondary study (Australian Bureau of Statistics, 2020), and other modelled risk (or protective) factors on the employment initiation rate; the net yearly flow from unemployment to full

employment is therefore $bwU - vE$. Net flows from unemployment to underemployment per year are specified in a similar way (the effects of distress and post-secondary study on transitions to full employment and underemployment are assumed to be the same). Underemployed people (i.e., those in employment working fewer hours per week than they would like) become fully employed at age-specific rates ckM, where M is the underemployed population, k is the base per capita rate that underemployed people secure full employment per year, and c is the effect of completing post-secondary education or vocational training on the underemployed to fully employed transition rate (see Wilkins, 2004, 2006). People in full employment become underemployed at age-specific rates zE, where E is the fully employed population and z is the per capita rate at which people transition from full employment to underemployment per year, so that the net flow from full employment to underemployment is $zE - ckM$. Fully employed and underemployed people leave the labour force (e.g., due to retirement, disability, parenting responsibilities) at constant per capita rates per year (separate age-specific rates are specified for people in full employment and those who are underemployed).

Net migration increases (or reduces) the fully employed population at age-specific rates $p_i I_i - q_i e_i P_i$, where p_i and q_i are the age-specific proportions of people in full employment among overseas arrivals and Australian residents, respectively, I_i is total age-specific immigration per year, e_i is the age-specific per capita emigration rate per year, and P_i is the number of people in age group i in the Australian population. The effects of migration on the unemployed and underemployed populations are modelled in the same way. Age-specific per capita mortality rates for people seeking employment are assumed to be 1.22 times those for people who are fully employed, underemployed, or not in the labour force (Sorlie and Rogot, 1990). Figure S10 presents participation and unemployment rate estimates derived from the system dynamics model and from labour force data published by the Australian Bureau of Statistics (2021).

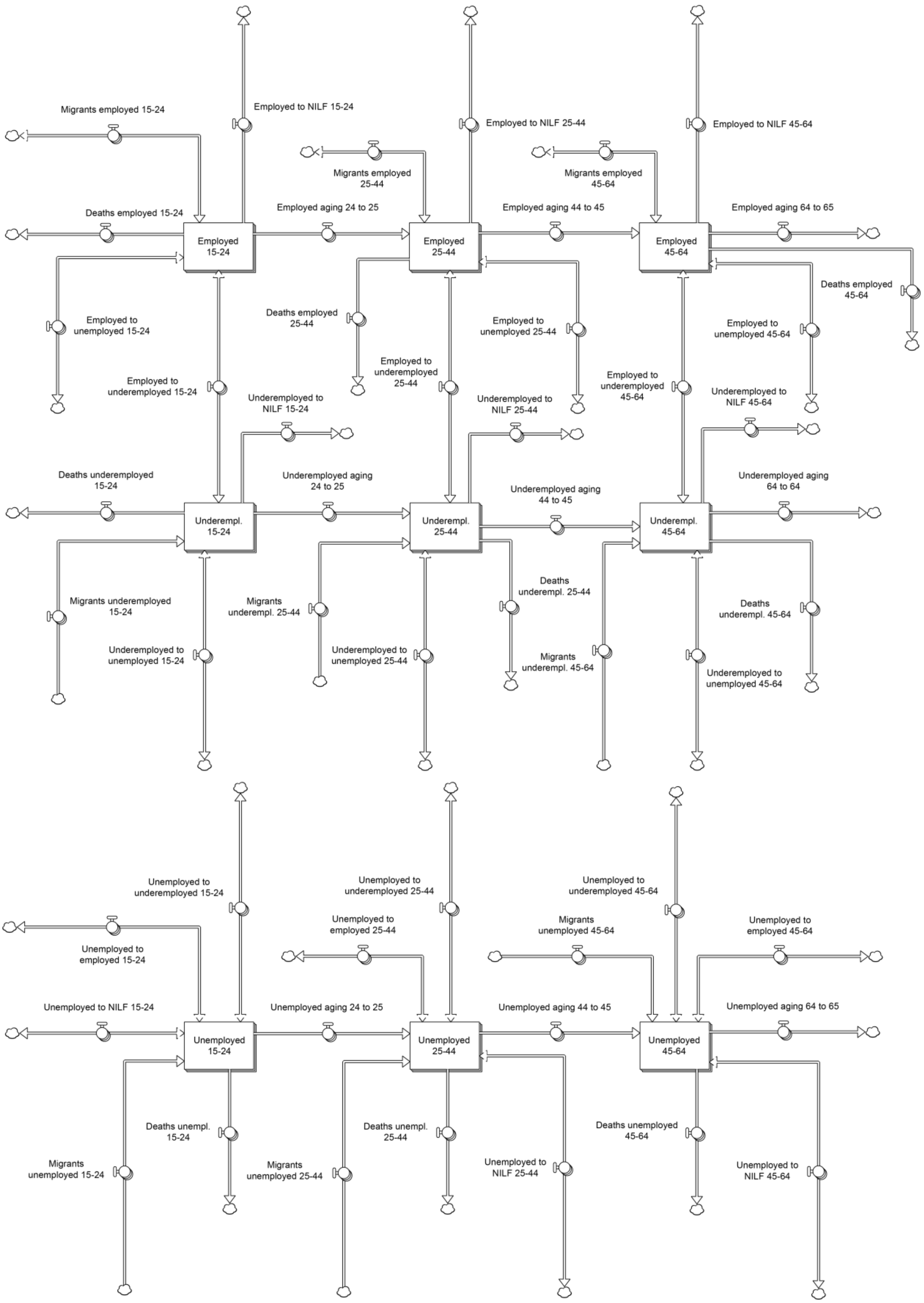

Figure S9. Stock and flow structure of the employment sector.

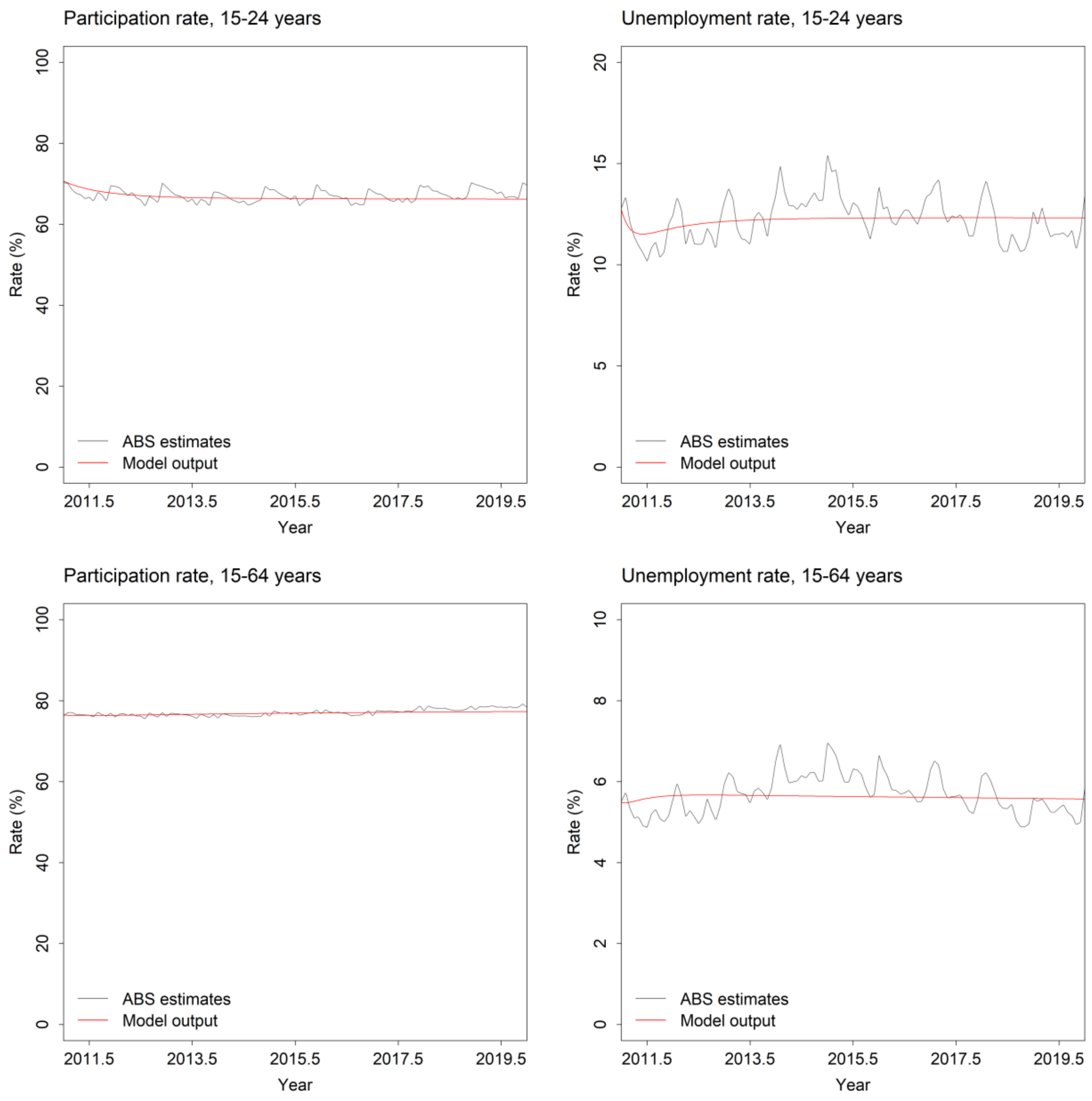

Figure S10. Participation and unemployment rate estimates derived from the system dynamics model and from Australian Bureau of Statistics (ABS; 2021) labour force data.

1.6. Substance abuse sector

The substance abuse sector captures the incidence of substance abuse disorders and the flow of patients through alcohol and other drug (AOD) treatment services (see figure S11). People aged 15−24 years and 25 years or more with chronic substance abuse disorders are divided among stocks corresponding to those not engaged with AOD treatment services, patients waiting for treatment services, and patients receiving treatment. New substance abuse disorder cases and people relapsing after treatment are added to the stocks of those not engaged with treatment services at age-specific rates $hsG + (1 - r)T$, where G is the number of people without a

substance abuse disorder, s is the base per capita rate at which people develop substance use disorders per year, h is the product of the effects of psychological distress (Marmorstein et al., 2010), adverse experiences in childhood (Crum et al., 2008), and homelessness (Johnson et al., 1997) on substance abuse disorder incidence, T is the number of people completing treatment per year, and r is the proportion of people recovering after treatment (24.1%; Miller et al., 2001). People with substance use disorders not engaged with treatment services flow into the stocks of those waiting for services at age-specific per capita rates that remain constant over the simulation period. Rates of treatment initiation depend on services capacity (i.e., the number of closed treatment episodes that can be provided per year), which is equal to $C_0 + \beta t$, where C_0 is services capacity at the start of the simulation period (i.e., 1 January 2011), β is the increase in capacity per year (estimated from services usage data for 2013−14 to 2016−17; Australian Institute of Health and Welfare, 2018), and t is time in years since the simulation start date. Patients waiting for treatment are assumed to disengage from services at a constant per capita rate estimated from data in Stevens et al. (2008).

Rates of treatment completion are equal to M/d, where M is the number of patients receiving treatment at any particular time point and d is the mean duration of care (19 days, or 0.052 years; Australian Institute of Health and Welfare, 2018). A constant proportion of patients completing treatment recover (24.1%; Miller et al., 2001), returning to the general population (i.e., people without a substance abuse disorder), while the remaining patients flow directly into the stocks of those with substance use disorders who are not currently engaged with AOD treatment services (see above). Net migration adds to (or subtracts from) the stocks of people with substance use disorders who are not engaged with services at age-specific rates $p_i I_i - e_i S_i$, where p_i is the age-specific prevalence of substance abuse disorders in the population, I_i is total age-specific immigration per year, e_i is the age-specific per capita emigration rate per year, and S_i is the number of residents in age group i who have a substance abuse disorder and are not engaged with treatment services. Age-specific per capita mortality rates for people with substance abuse disorders are assumed to be 1.95 times those for the general population (Roerecke and Rehm, 2013). Figure S12 presents model-based estimates of the prevalence of substance abuse disorders and treatment services usage rates together with estimates derived from the NSW Population Health Survey (see http://www.healthstats.nsw.gov.au/), the National Health Survey (Australian Bureau of Statistics, 2015), and services usage data published by the Australian Institute of Health and Welfare (2018a).

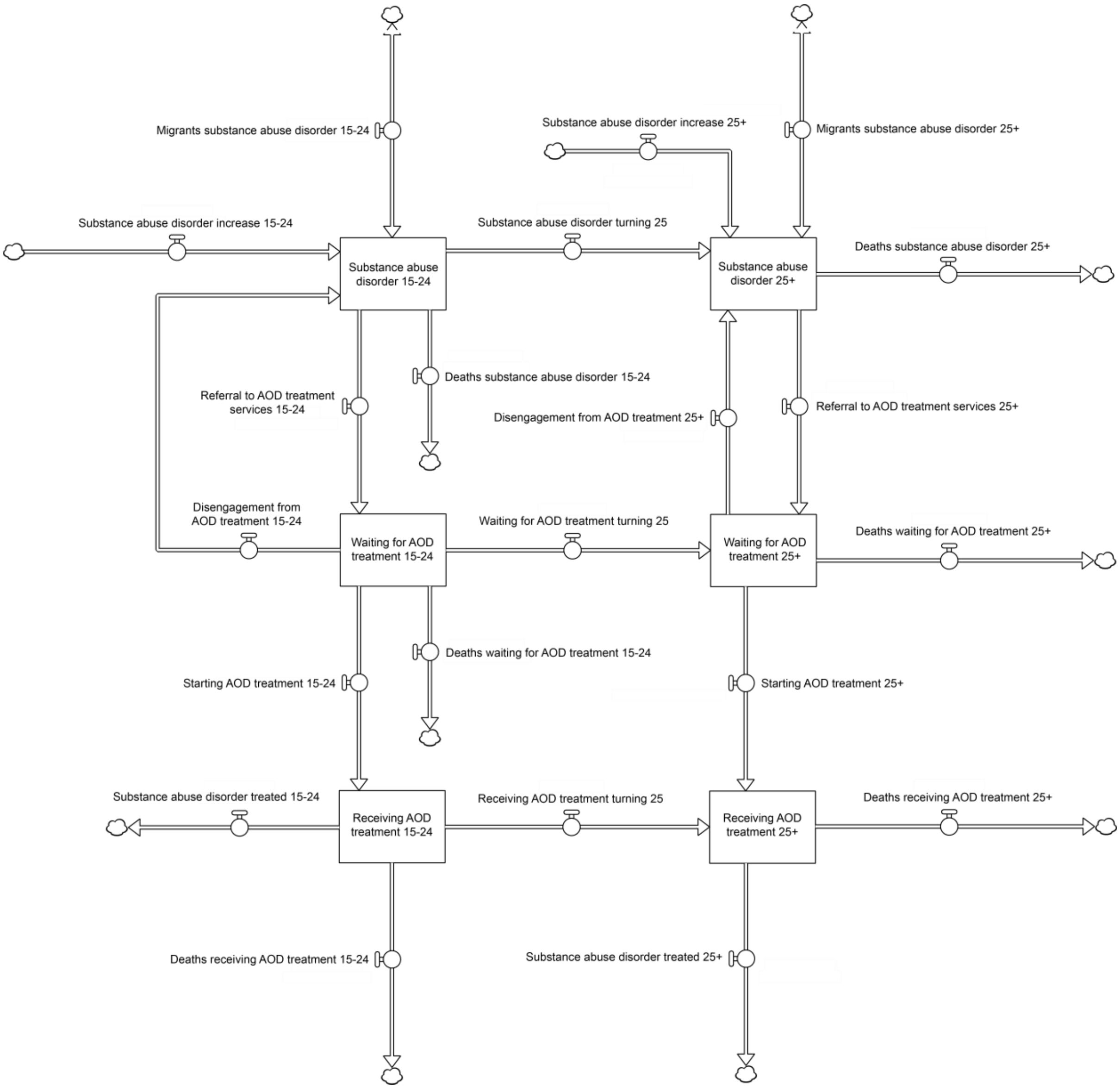

Figure S11. Stock and flow structure of the substance abuse disorder sector.

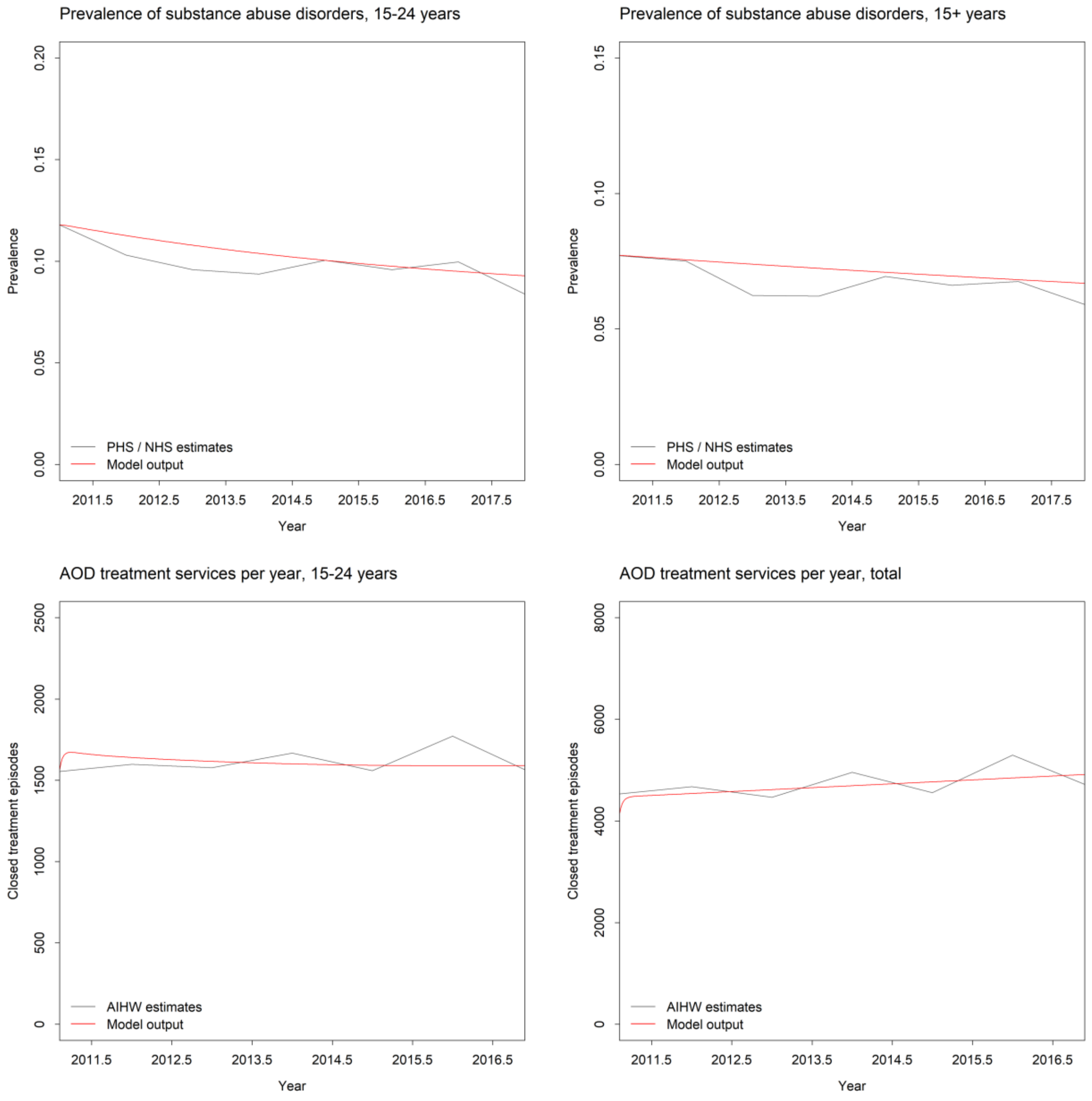

Figure S12. Estimates of the prevalence of substance abuse disorders and treatment services usage rates derived from the system dynamics model and from the NSW Population Health Survey (PHS), the National Health Survey (NHS), and services usage data published by the Australian Institute of Health and Welfare (AIHW).

1.7. Domestic violence and homelessness sectors

The structure of the domestic violence sector is shown in figure S13. Age-specific base rates of intimate partner violence are modelled as stocks that increase (65+ years) or decrease (15−24 years and 25−64 years) at a constant rate per year. The incidence of intimate partner violence in age group i is equal to $f_i v_i$, where v_i is the base number of incidents per year and f_i is the product of the effects of partner substance abuse and partner unemployment on the domestic violence rate (Kyriacou et al., 1999). Rates of increase (or decrease) in the base

rates, v_i, were set so that the incidence of intimate partner violence in each age group aligned with estimates derived from Australian Bureau of Statistics data on domestic violence-related offences (Australian Bureau of Statistics, 2019b; see figure S14).

Figure S15 presents the structure of the homelessness sector, which models age-specific transitions into and out of homelessness. The homeless population of the catchment area is represented as four stocks, corresponding to people aged 0−14 years, 15−24 years, 25−64 years, and 65 years and above satisfying the statistical definition of homelessness developed by the Australian Bureau of Statistics (2012b). People flow into these stocks at age-specific rates equal to $r_i h_i N_i + q s_i P_i$, where P_i and N_i are, respectively, the total number of people in age group i and the number of people in age group i with secure housing, h_i is the reference (i.e., base) per capita rate at which people in age group i with secure housing become homeless per year (for reasons other than domestic violence), r_i is the product of the age-specific effects of childhood adversity (Herman et al., 1997) and substance use disorders (Shelton et al., 2009) on the risk of homelessness, s_i is the age-specific domestic violence rate, and q is the probability of becoming homeless after a domestic violence incident (0.023; Australian Bureau of Statistics, 2017). Homelessness declines as people secure housing at age-specific rates H_i/d, where H_i is the homeless population for age group i and d is the mean duration of homelessness (20.4 weeks, or 0.392 years; Australian Bureau of Statistics, 2016). Per capita mortality rates for homeless people are assumed to be 1.60 times those for people with secure housing (Morrison, 2009). Estimates of the homeless population derived from the model are presented alongside Australian Bureau of Statistics (2018a) estimates in figure S14.

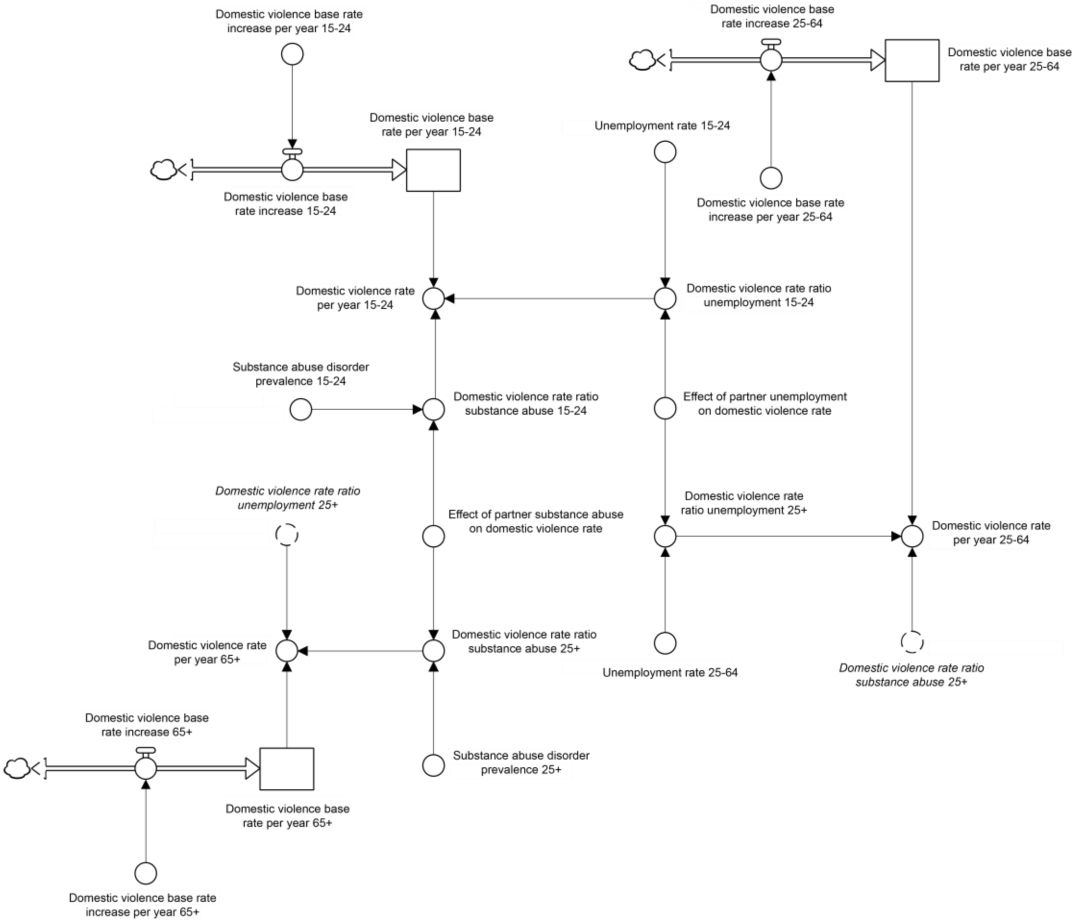

Figure S13. Structure of the domestic violence sector.

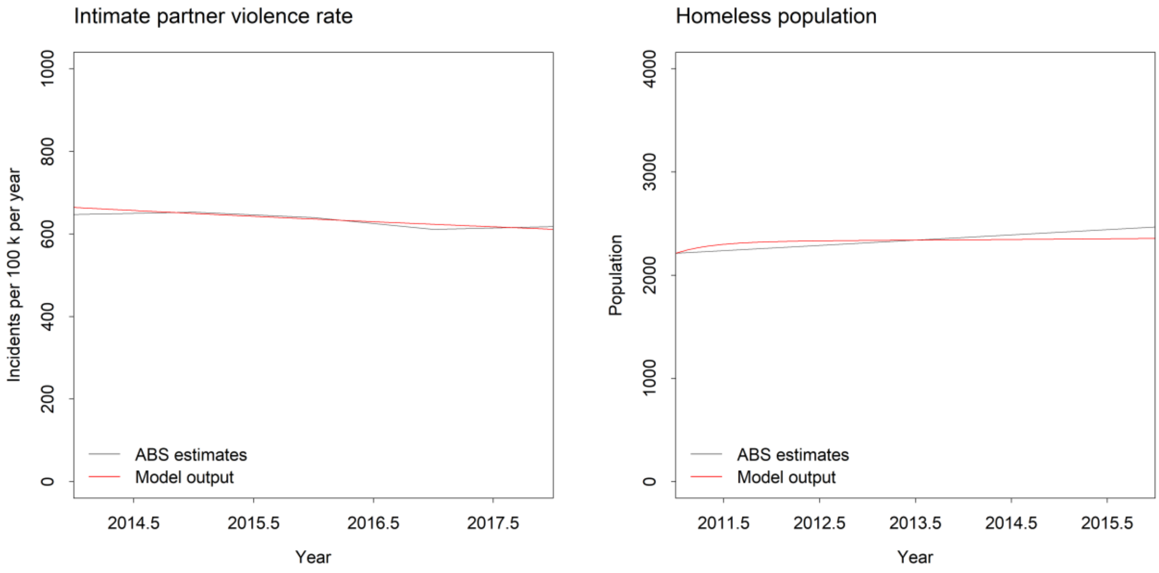

Figure S14. Estimates of the incidence of intimate partner violence and the homeless population derived from the system dynamics model and from Australian Bureau of Statistics (ABS) data.

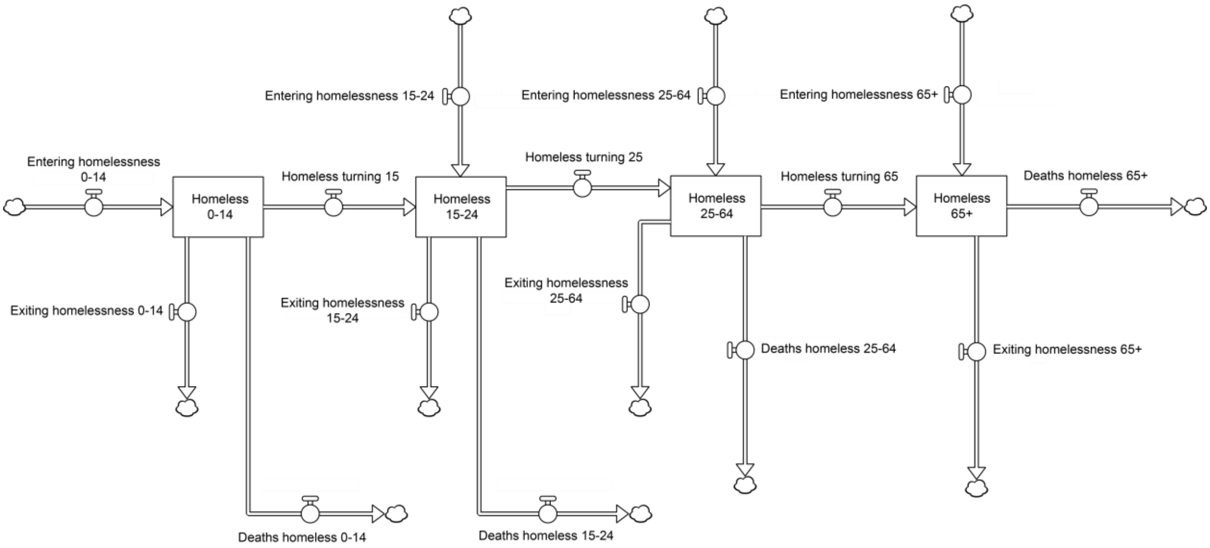

Figure S15. Stock and flow structure of the homelessness sector.

1.8. Mental health services sector

Figure S16 shows a high-level map of the mental health services sector, which models the movement of patients through the mental health care system. People experiencing low or moderate to very high psychological distress engage with mental health services in one of two ways; they may perceive a need for mental health care and seek help (e.g., from a general practitioner or online services), or they may present to an emergency department (e.g., for self-harm) without having previously perceived a need for treatment. After engaging with mental health services, patients may recover following treatment, returning to the general population of people with low psychological distress and no perceived need for care, be treated but not recover, or disengage due to excessive waiting times (a result of insufficient services capacity) or because they are dissatisfied with the care they receive. Patients who are treated but do not recover return to perceiving a need for services and will eventually seek help again if they do not recover spontaneously; thus, people entering the mental health care system continue receiving treatment (modelled as individual service contacts; see below) until they recover, disengage, or die (mortality is captured in the model, but is not shown in figure S16). The principal components of the mental health services sector are described in detail below.

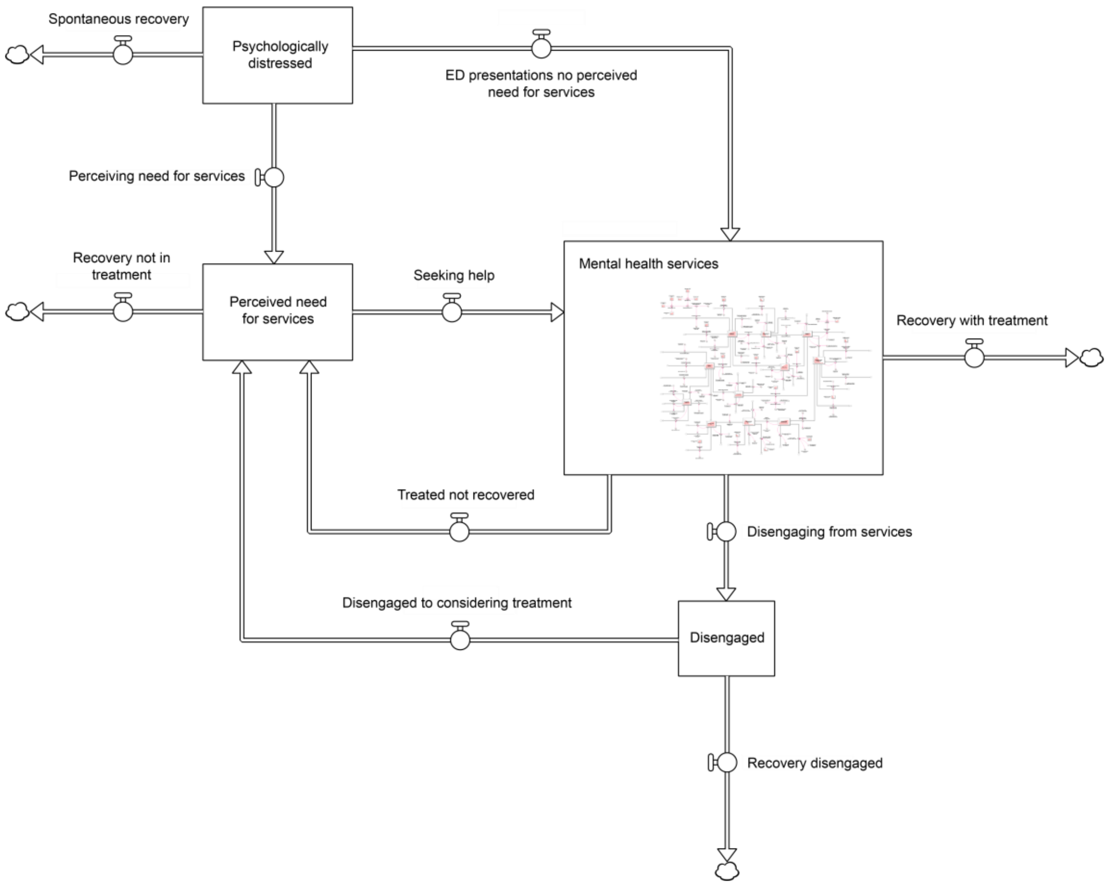

Figure S16. High-level map of the mental health services sector.

Help seeking — People experiencing low or moderate to very high psychological distress who are not currently considering engaging with mental health services perceive a need for care at rates equal to $p_i D_i$, where D_i is the number of people with distress level i not currently considering treatment and p_i is the per capita rate that people with distress level i perceive a need for care per year. The per capita rates p_i are assumed to increase at a constant rate per year due to increasing public awareness of high-prevalence mental disorders and available treatment options. After perceiving a need for treatment, people engage with mental health services at per capita rates that depend on their age and state of psychological distress (Australian Bureau of Statistics, 2012a). Recently treated patients who have not recovered or disengaged from services return to perceiving a need for care and may attend subsequent (planned or unplanned) appointments with a GP or community-based psychiatric services (i.e., psychiatrists and allied health services, hospital outpatient services), be admitted to a general or private hospital, commence online treatment, or present to an emergency department (e.g., for suicidal ideation); the arrayed stock of people perceiving a need for services (see figure S17) therefore contains a mix of prospective patients and patients already engaged with the mental health services system. These prospective and current patients may age, recover spontaneously, or transition from a state of low (mild)

psychological distress to a state of moderate to very high distress, and are assumed to experience the same per capita mortality rates as similar-aged people with the same level of distress who are not considering treatment.

GP and online services — People with a perceived need for mental health services seek help from a GP at age-specific rates $\theta_j s_i P_{ij}$, where P_{ij} is the number of people in age group i with distress level j perceiving a need for care (those in the arrayed stock labelled 'Perceived need for services' in figure S17), s_i is the age-specific per capita rate at which people with low distress and a perceived need for care seek help from a GP, and the rate ratio θ_j is equal to 1 for people with low distress and less than 1 for people experiencing moderate to very high distress (i.e., people with moderate to very high psychological distress are assumed to seek help at a lower per capita rate than those with low distress, given a perceived need for care; see Australian Bureau of Statistics, 2012a). The per capita rates s_i are assumed to increase at a constant rate per year, increasing help seeking rates among people who perceive a need for services. Prior to receiving care, patients seeking help from a GP or referred to GP services after completing hospital inpatient care wait for a varying period of time that depends on services capacity and the total number of patients waiting for care. GP services capacity, i.e., the number of mental health-related GP consultations that can be provided per year, is assumed to increase at a constant rate per year, estimated from Medicare Benefits Schedule (MBS) claims data for the period 2011−2019 (see figure S18).

Mental health-related GP consultations are represented as a stock (arrayed by age group and distress level) with outflows corresponding to treatment completion, referral to other services (including psychiatrist and allied health services, community mental health care services, and online services), and disengagement resulting from dissatisfaction with the care provided. Age-specific recovery rates among patients completing treatment are equal to $v\gamma_j r T_{ij}$, where T_{ij} is the number of patients in age group i with distress level j completing treatment per year, v is the proportion of patients completing treatment who receive psychological therapy, r is the proportion of patients with low (or mild) distress who recover after receiving psychological therapy, and the recovery rate ratio γ_j is equal to 1 for mildly distressed patients and less than 1 for patients experiencing moderate to very high psychological distress (i.e., psychological therapy provided by a GP is assumed to be less effective for moderately to highly distressed patients than for mildly distressed patients; see Cuijpers et al., 2009). Patients completing treatment who do not recover return to the stocks of people who perceive a need for care.

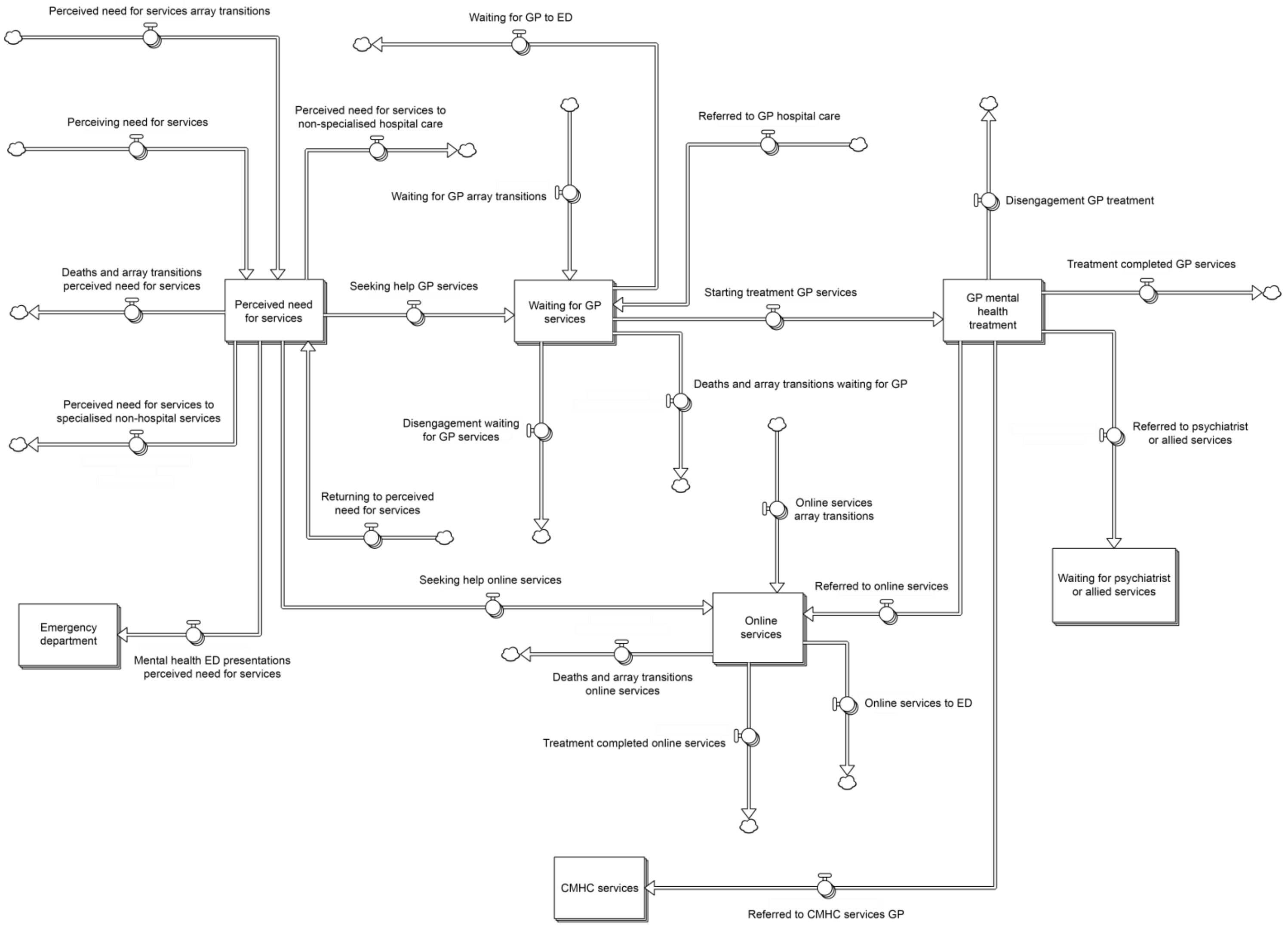

Figure S17. Stock and flow structure of the help-seeking, general practitioner (GP) services, and online services components of the mental health services sector.

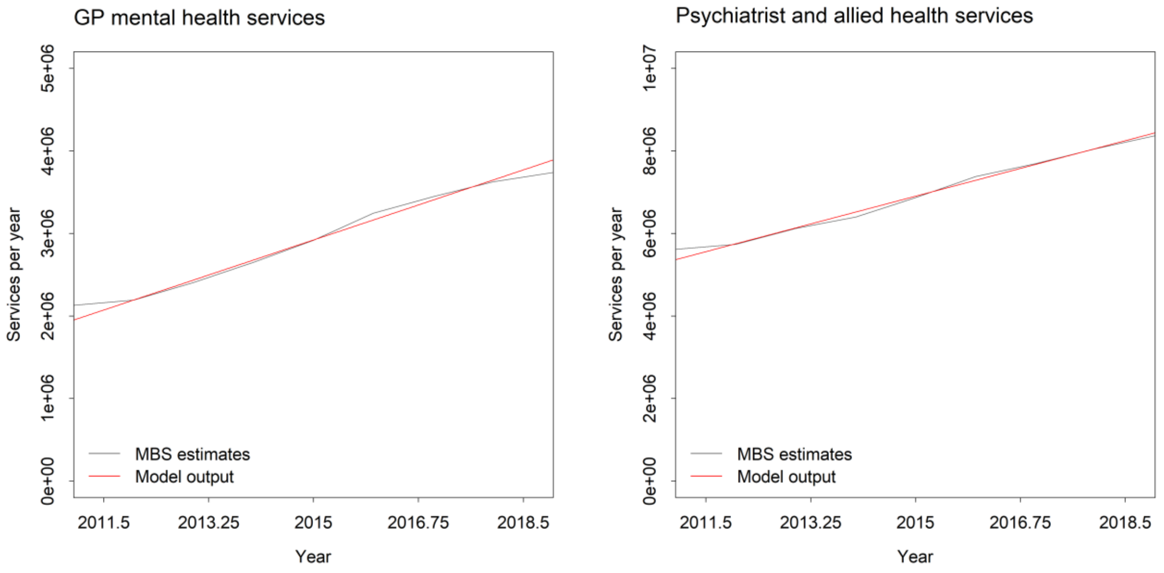

Figure S18. General practitioner (GP) and psychiatrist and allied health services usage rates derived from the system dynamics model and from Medicare Benefits Schedule (MBS) data (available at: https://www.aihw.gov.au/reports/mental-health-services/mental-health-services-in-australia/data).

Age-specific treatment commencement rates for online (self-help) services are equal to $h_j P_{ij} + uG_{ij}$, where P_{ij} is the number of people in age group i with psychological distress level j who perceive a need for services (i.e., those in the arrayed stock labelled 'Perceived need for services' in figure S17), h_j is the per capita rate that people with distress level j and a perceived need for care access online services per year, G_{ij} is the GP services provision rate (i.e., the number of patients in age group i with psychological distress level j attending a GP consultation per year), and u is the fraction of patients visiting a GP for a mental health issue referred to online services. Prior to completing treatment, people accessing online services may recover spontaneously, present to an emergency department, or die (see figure S17); treatment completion rates are equal to O_{ij}/d, where O_{ij} is the number of people in age group i with distress level j engaged in online treatment and d is the mean duration of online treatment programs (assumed to be 6 weeks, or 0.115 years; Christensen et al., 2004). People completing online treatment programs recover at rates equal to $\eta_j z\, O_{ij}/d$, where z is the fraction of people with low (or mild) psychological distress completing treatment who recover and the rate ratio η_j is equal to 1 for mildly distressed people and significantly less than 1 for people with moderate to very high distress (i.e., online services are assumed to be more effective for people with a low level of distress than for people with moderate to very high levels of distress).

Psychiatrist and allied health services — Figure S19 presents the structure of the psychiatrist and allied health services component of the mental health services sector. Prior to receiving treatment, patients referred to a psychiatrist or allied health services by a GP or after completing hospital inpatient care wait for a period of time that depends on services capacity and the total number of patients waiting for care. The arrayed stock of people waiting for treatment also contains patients currently engaged with specialised services who have planned (follow-up) appointments (these patients and patients referred to services after receiving hospital care enter via the flow labelled 'Additional psychiatrist or allied health services'; see figure S19). Services capacity, i.e., the number of psychiatrist and allied health services that can be provided per year, increases at a constant rate per year, estimated from MBS claims data for the period 2011−2019 (see figure S18). Patients receiving treatment are referred to psychiatric hospital services, disengage from the mental health services system due to dissatisfaction with the care received, recover, or return to perceiving a need for care (these patients flow back into the arrayed stock labelled 'Perceived need for services'; see figure S17). Recovery rates are equal to rT_{ij}, where T_{ij} is the number of patients in age group i with psychological distress level j who complete treatment per year and r is the fraction of patients recovering after receiving specialised psychiatric care. Psychiatrist and allied health services usage rates derived from the model and from MBS claims data are presented in figure S18.

Hospital services — Mental health-related public hospital services captured in the model include psychiatric and non-specialised inpatient care, outpatient care delivered by community mental health care (CMHC) teams, and emergency department attendances (figure S20). Emergency department (ED) presentation rates are equal to $\theta_j e_i C_{ij} + \theta_j f_i N_{ij}$, where C_{ij} is the number of people in age group i with psychological distress level j perceiving a need for care (including all patients waiting for care or engaged in online treatment and those who have disengaged from services; see figures S17, S19, S20, S22, S23), N_{ij} is the number of people in age group i with distress level j who do not perceive a need for services, e_i and f_i are age-specific per capita ED presentation rates for mildly distressed people with and without a perceived need for care, respectively, and the rate ratio θ_j is equal to 1 for people with low (mild) psychological distress and more than 1 for people experiencing moderate to very high distress (i.e., people in a state of moderate to very high distress are assumed to present to EDs at a higher per capita rate than those with a low level of distress). People presenting to an ED may be admitted to a psychiatric or general hospital ward, referred to CMHC services, or discharged to the community. A fraction of patients perceiving a need for care who are discharged to the community disengage from services due to dissatisfaction with the care provided.

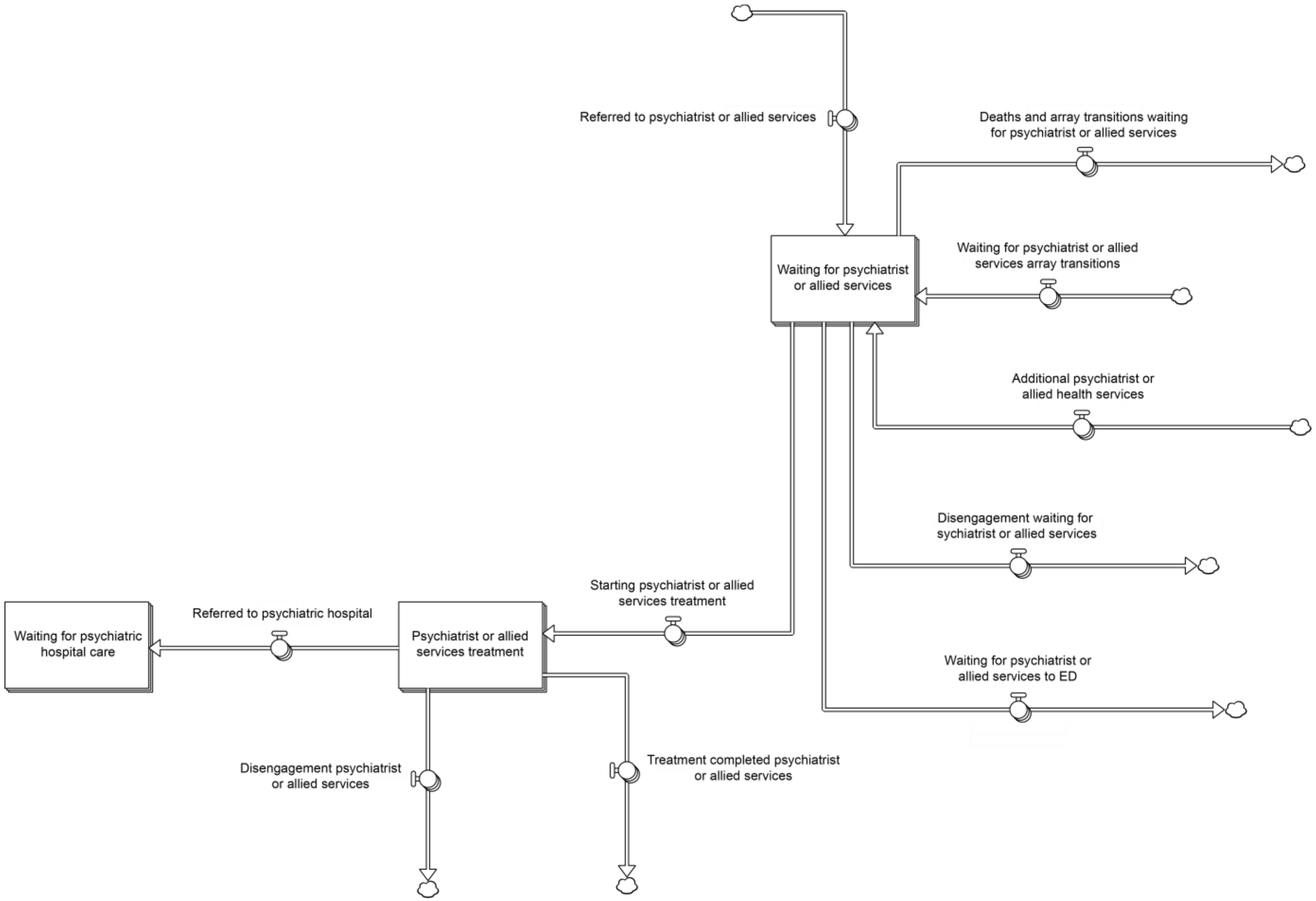

Figure S19. Stock and flow structure of the psychiatrist and allied health services component of the mental health services sector.

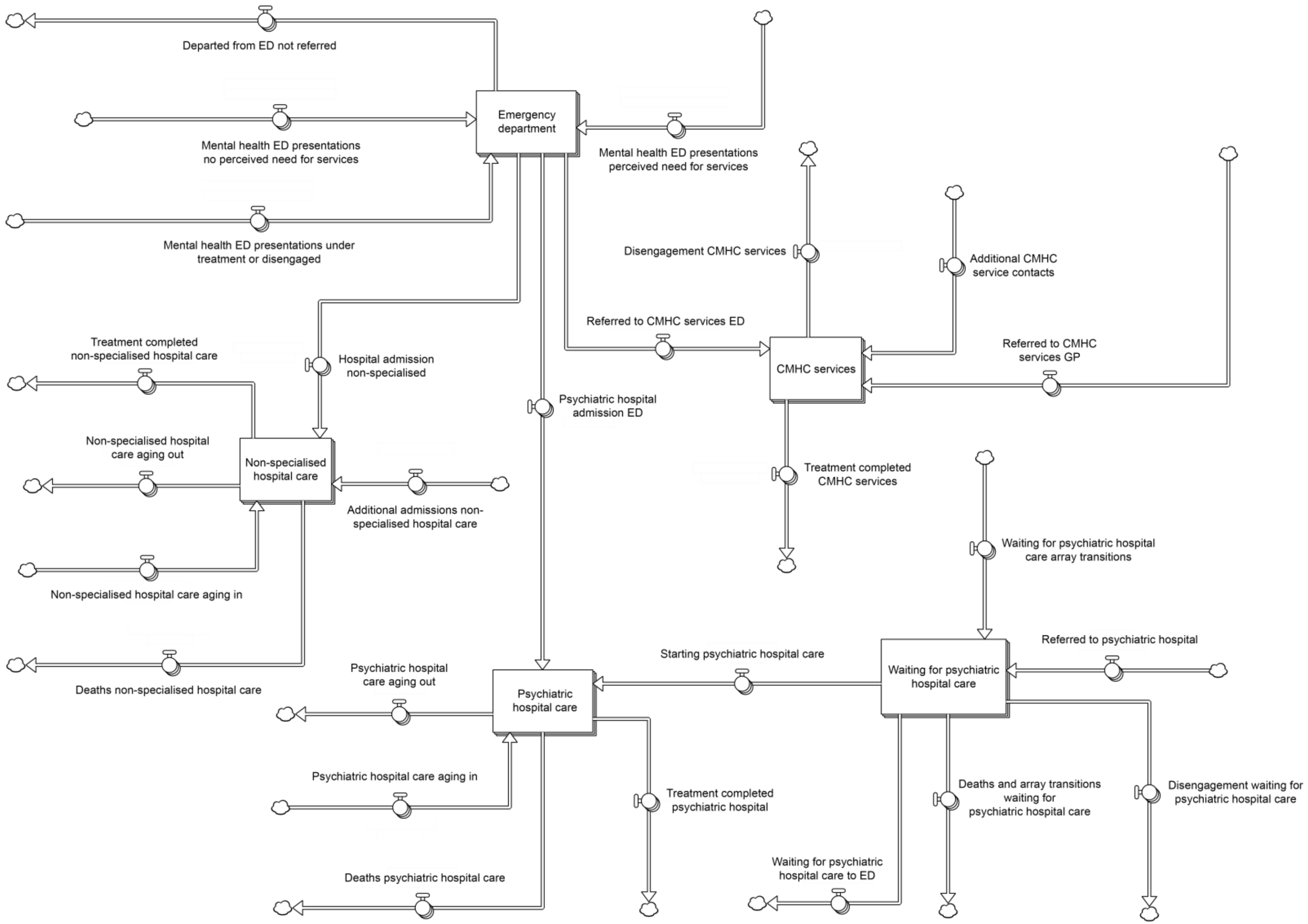

Figure S20. Stock and flow structure of the hospital services component of the mental health services sector.

Psychiatric and general hospital admission rates are constrained by hospital capacity (i.e., the total numbers of patients that can be admitted annually), which is assumed to increase linearly over the simulation period; capacity increase rates per year were estimated from mental health-related hospital separations data published by the Australian Institute of Health and Welfare (2019; see figure S21). Psychiatrists refer patients to psychiatric hospital care at rates $q_i M_{ij}$, where M_{ij} is the number of patients in age group i with distress level j receiving psychiatrist and allied health services per year and q_i is the age-specific proportion of patients receiving psychiatrist and allied health services referred to psychiatric hospital care. Prior to being admitted, patients referred to a psychiatric hospital wait for a period of time that depends on hospital capacity, the total number of referred patients waiting for care, and the rate at which patients are being admitted via EDs (since available capacity declines as the ED-related admission rate increases). People with a perceived need for services are admitted for general (non-specialised) hospital care without presenting to an ED (e.g., for allied health interventions) at constant per capita rates that depend on age and distress level (the flow labelled 'Additional admissions non-specialised hospital care' in figure S20). A proportion of patients discharged from psychiatric or general hospital care disengage from services due to dissatisfaction with the care provided; patients who do not disengage from care are referred to a GP, psychiatrist and allied health services, or CMHC services for follow-up care.

Age-specific community mental health care (CMHC, or hospital outpatient) services provision rates are equal to $\eta_j h_i G_{ij} + pE_{ij} + S_{ij} + A_{ij} + k_j u_i P_{ij}$, where G_{ij} is the number of patients in age group i with distress level j visiting a GP per year, h_i is the proportion of mildly distressed patients in age group i visiting a GP who are referred to CMHC services, E_{ij} is the number of people presenting to an ED per year not admitted to hospital, p is the proportion of patients discharged from an ED referred to CMHC services, S_{ij} and A_{ij} are, respectively, the numbers of patients discharged from psychiatric and general hospital inpatient care per year referred to CMHC services, P_{ij} is the number of people with a perceived need for care (those in the arrayed stock labelled 'Perceived need for services'; figure S17), u_i is the per capita CMHC services contact rate among people with a perceived need for care (e.g., for follow-up appointments), and the rate ratios η_j and k_j are equal to 1 for patients with low psychological distress and more than 1 for patients with moderate to very high psychological distress. Patients receiving CMHC services recover, return to the stocks of people perceiving a need for services, or disengage from the mental health care system due to dissatisfaction with the care provided (figure S20). The per-service recovery rate for patients who do not disengage from treatment is calculated as rK/T,

where K is the total number of CMHC services that can be provided per year while maintaining the base (or reference) per-service recovery rate r and T is the current CMHC services provision rate; note that as the current services provision rate, T, increases relative to the reference capacity K, the per-service recovery rate, rK/T, declines (due to increased pressure on services). CMHC services capacity (i.e., K) is assumed to increase at a constant rate per year, estimated from services usage data published by the Australian Institute of Health and Welfare (2019; figure S21).

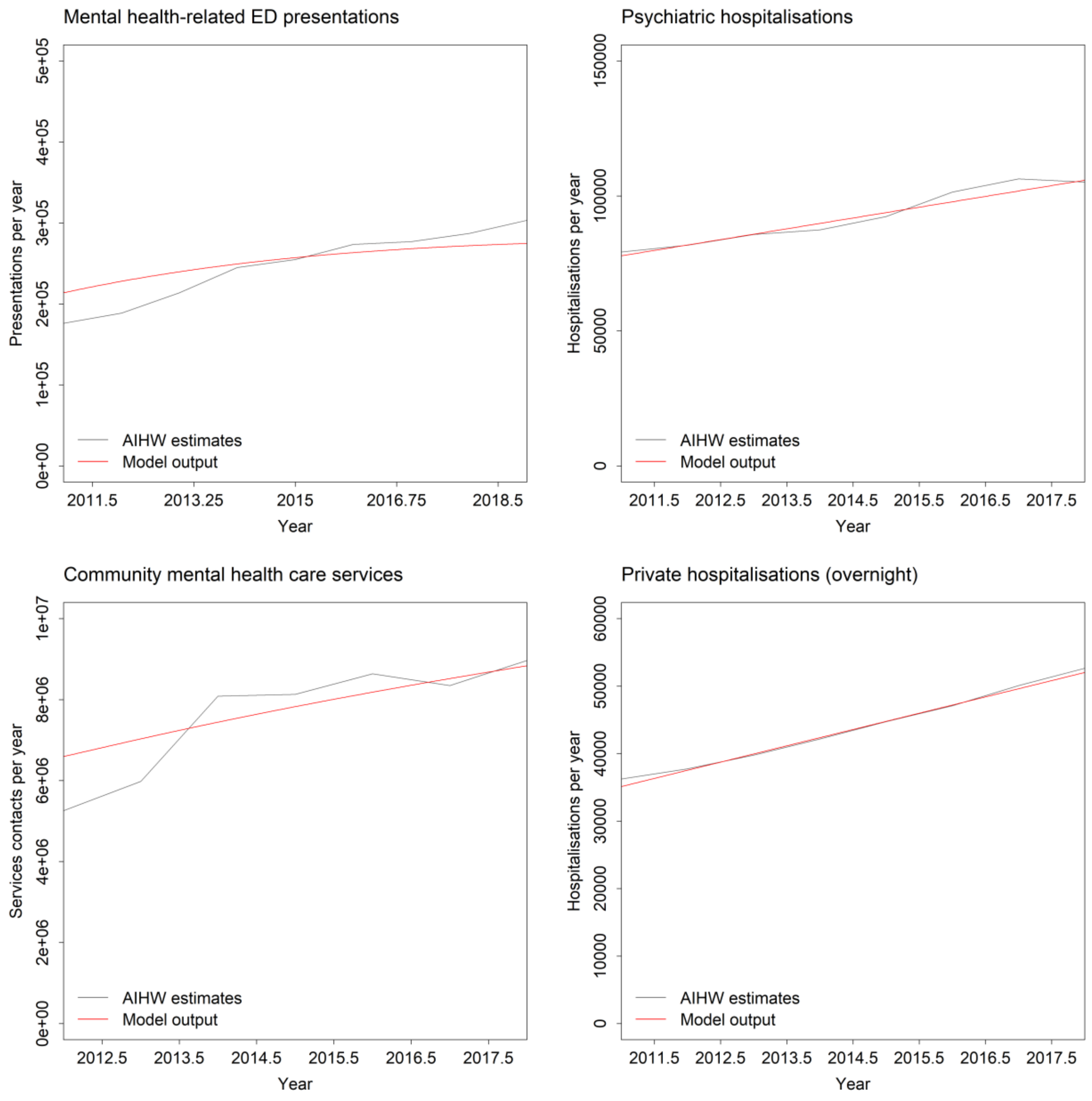

Figure S21. Hospital services usage rates derived from the system dynamics model and from data published by the Australian Institute of Health and Welfare (AIHW; available at: https://www.aihw.gov.au/reports/mental-health-services/mental-health-services-in-australia/data).

Private mental health services — The structure of the private services component of the mental health services sector, which captures overnight inpatient care provided in private hospitals and community-based private mental health services (including hospital outpatient and same-day inpatient care; see Australian Institute of Health and Welfare, 2019), is shown in figure S22. Private mental health services are assumed to operate independently of the government financed mental health services described above (there are no direct referral pathways connecting public and private services), although individual patients may access both private and public services at different times. Patients are referred to private hospitals for overnight inpatient care at age-specific rates $s_i P_i$, where P_i is the number of people in age group i perceiving a need for care (those in the arrayed stock labelled 'Perceived need for services' in figure S17) and s_i is the age-specific per capita referral rate per year. Prior to being admitted, patients referred to a private hospital wait for a period of time that depends on hospital capacity and the total number of patients waiting for treatment. Private hospital capacity, i.e., the number of patients that can be admitted annually, is assumed to increase at a constant rate per year, estimated from data published by the Australian Institute of Health and Welfare (2019; see figure S21). A proportion of patients discharged following overnight inpatient care disengage from the services system due to dissatisfaction with the care provided; patients who do not disengage are referred to community-based private mental health services for follow-up care.

Age-specific rates of referral to community-based private services are equal to $u_i P_i + (1 - d) H_i$, where P_i is the number of people in age group i perceiving a need for care (i.e., those in the arrayed stock labelled 'Perceived need for services' in figure S17), u_i is the age-specific per capita referral rate per year, H_i is the number of private hospital patients in age group i discharged from overnight inpatient care per year, and d is the proportion of discharged patients disengaging from mental health services due to dissatisfaction with the care provided. Prior to receiving treatment, patients referred to community-based private services wait for a period of time that depends on services capacity and the total number of referred patients waiting for care. Services capacity, i.e., the total number of community-based services that can be delivered per year, is assumed to increase at a constant rate per year, estimated from data published by the Australian Institute of Health and Welfare (2019). Patients receiving treatment disengage from the services system due to dissatisfaction with the care provided, recover, or return to perceiving a need for care (these patients flow back into the arrayed stock labelled 'Perceived need for services'; figure S17). Recovery rates are equal to rT_{ij}, where T_{ij} is the number of patients

in age group i with psychological distress level j completing treatment per year and r is the fraction of patients recovering after receiving specialised psychiatric care.

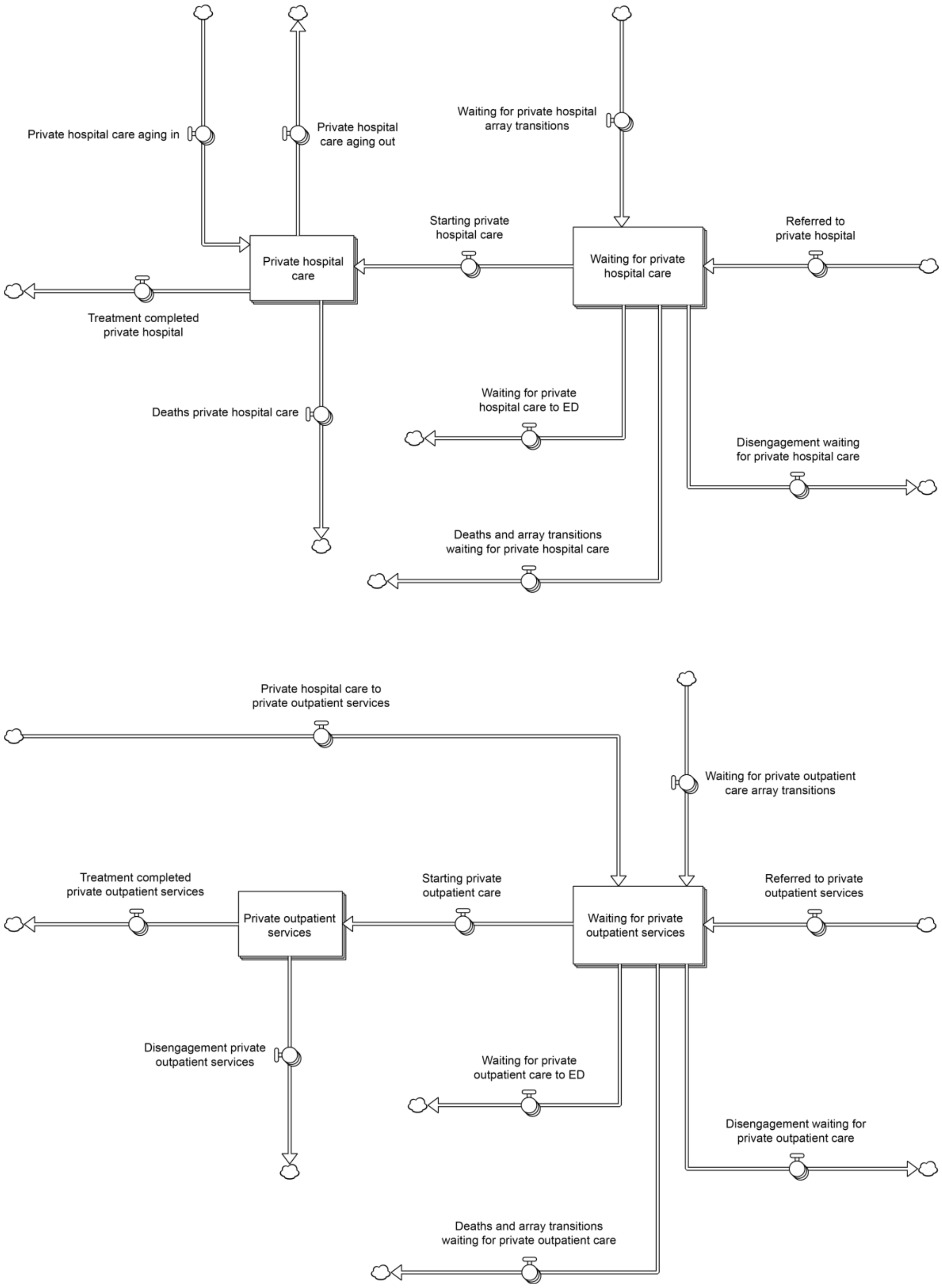

Figure S22. Stock and flow structure of the private mental health services component of the mental health services sector.

Disengagement — Patients waiting for a GP, psychiatrist and allied health services, psychiatric hospital care, or private services are assumed to disengage from the mental health care system at a constant per capita rate per year (estimated from data reported in Tyrer et al., 1995). The total disengagement rate therefore increases whenever the demand for mental health services exceeds services capacity, as the number of patients waiting for care continues to increase while patients are being referred to services at a higher rate than they can be treated. Patients receiving treatment also disengage from services due to dissatisfaction with the care provided (figures S17, S19, S20, S22). Disengagement is assumed to increase the age-specific per capita rates at which people with low psychological distress transition to a state of moderate to very high psychological distress, due to a loss of hope that effective treatment is available, or trauma associated with unsatisfactory care (see Australian Bureau of Statistics, 2012a). Patients who have disengaged from the services system return to the arrayed stock of people perceiving a need for care (i.e., they consider engaging with services again) at a constant per capita rate per year (figure S23).

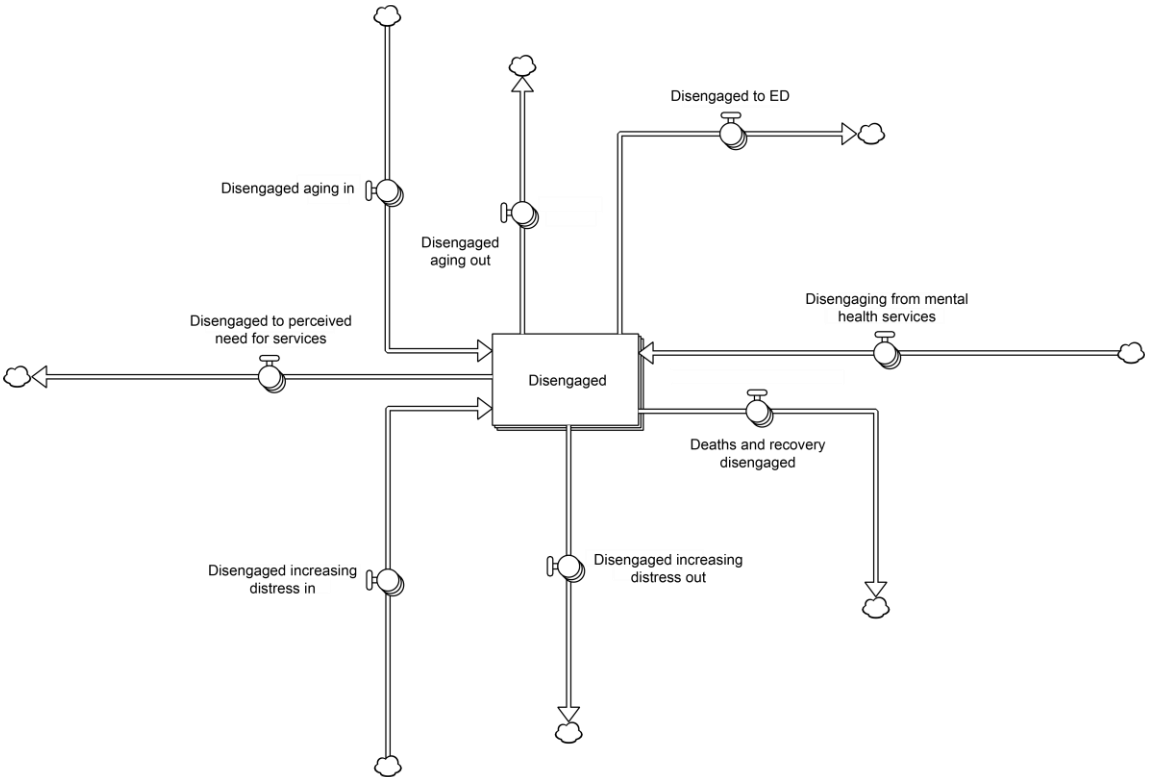

Figure S23. Stock and flow structure of the disengagement component of the mental health services sector.

1.9. Suicidal behaviour sector

Figure S24 presents the structure of the suicidal behaviour sector, which captures self-harm hospitalisations and suicide deaths (note that we equate suicide attempts with intentional self-harm hospital admissions due to data availability constraints). Age-specific suicide attempt rates are calculated as $s_i M_i + \theta s_i H_i$, where M_i and H_i are the numbers of people in age group i experiencing low (mild) psychological distress and moderate to very high psychological distress, respectively, s_i is the per capita suicide attempt rate for mildly distressed people in age group i, and the suicide attempt rate ratio θ is assumed to be substantially greater than 1 (i.e., the per capita attempt rate for people with mild distress is assumed to be lower than that for people experiencing moderate to very high distress; Chamberlain et al., 2009). The number of suicide deaths per year is calculated as λa, where a is the suicide attempt rate and λ is attempt lethality (i.e., the proportion of suicide attempts that are fatal). Intentional self-harm hospitalisation and suicide death rate estimates derived from the system dynamics model and from data published online by the Australian Institute of Health and Welfare (available at: https://www.aihw.gov.au/suicide-self-harm-monitoring/data) are presented in figure S25.

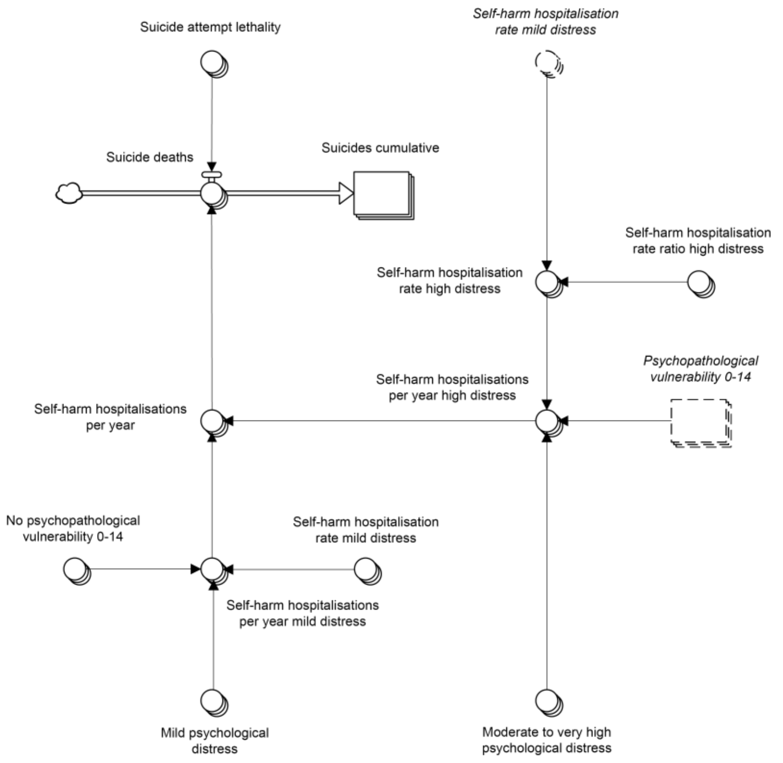

Figure S24. Structure of the suicidal behaviour sector.

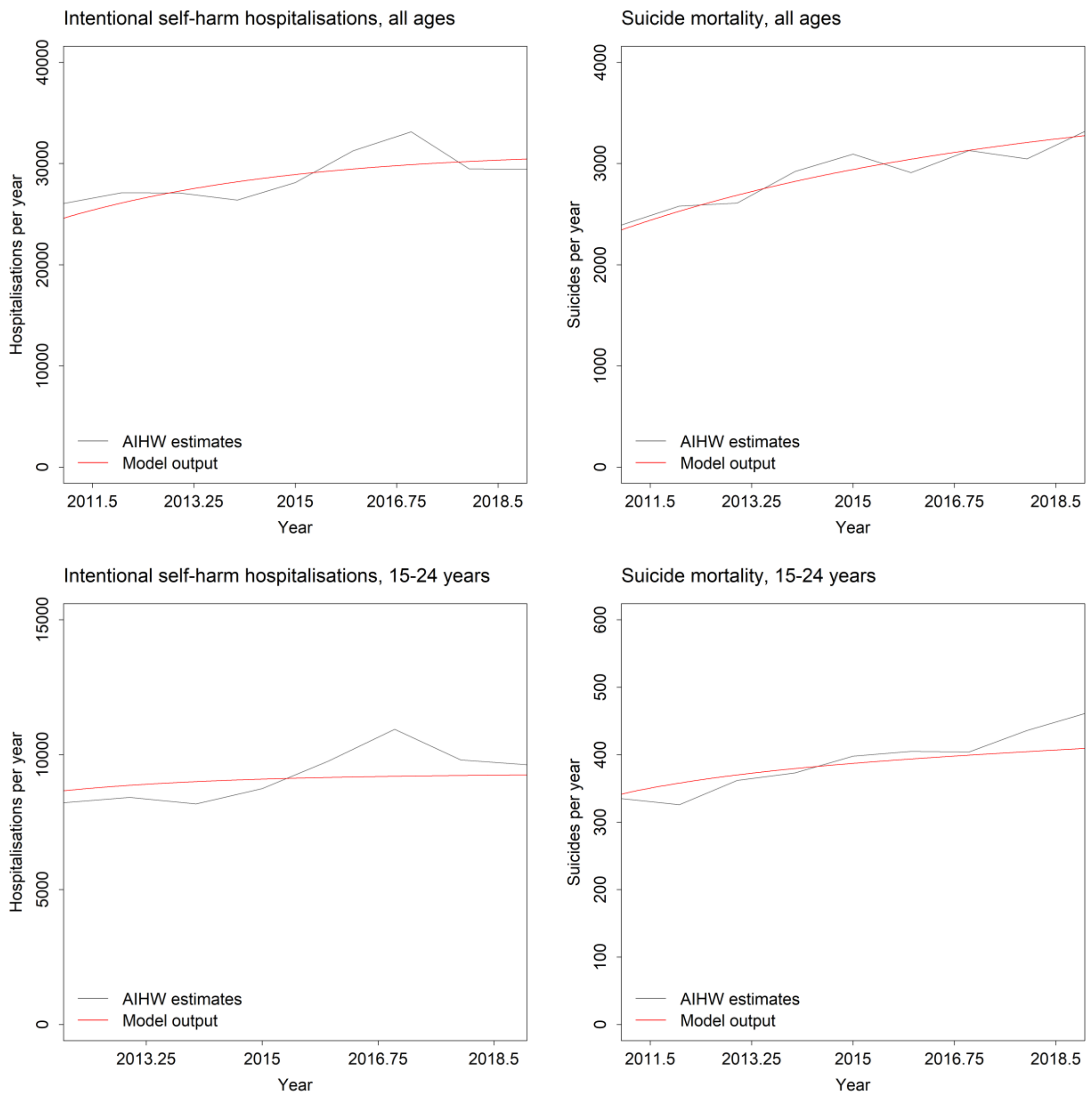

Figure S25. Intentional self-harm hospitalisation and suicide mortality rate estimates derived from the system dynamics model and from data published online by the Australian Institute of Health and Welfare (available at: https://www.aihw.gov.au/suicide-self-harm-monitoring/data)

References

Australian Bureau of Statistics, 2012a. Information paper. Use of the Kessler psychological distress scale in ABS health surveys, Australia, 2007–08. Cat. no. 4817.0.55.001. Australian Bureau of Statistics, Canberra.

Australian Bureau of Statistics, 2012b. Information paper – A statistical definition of homelessness, Australia. Cat. no. 4922.0. Australian Bureau of Statistics, Canberra.

Australian Bureau of Statistics, 2015. National Health Survey: first results, 2014-15. Cat. no. 4364.0.55.001. Australian Bureau of Statistics, Canberra.

Australian Bureau of Statistics, 2016. Mental health and experiences of homelessness, Australia, 2014. Cat. no. 4329.0.00.005. Australian Bureau of Statistics, Canberra.

Australian Bureau of Statistics, 2017. Personal safety, Australia, 2016. Cat. no. 4906.0. Australian Bureau of Statistics, Canberra.

Australian Bureau of Statistics, 2018a. Census of Population and Housing: Estimating homelessness, 2016. Cat. no. 2049.0. Australian Bureau of Statistics, Canberra.

Australian Bureau of Statistics, 2018b. National Health Survey: first results, 2017–18. Cat. no. 4364.0.55.001. Australian Bureau of Statistics, Canberra.

Australian Bureau of Statistics, 2019a. Australian demographic statistics, Jun 2019. Cat. no. 3101.0. Australian Bureau of Statistics, Canberra.

Australian Bureau of Statistics, 2019b. Recorded crime – victims, Australia, 2018. Cat. no. 4510.0. Australian Bureau of Statistics, Canberra.

Australian Bureau of Statistics, 2020. Education and work, Australia, May 2020. Cat. no. 6227.0. Australian Bureau of Statistics, Canberra.

Australian Bureau of Statistics, 2021. Labour force, Australia, Mar 2021. Cat. no. 6202.0. Australian Bureau of Statistics, Canberra.

Australian Institute of Health and Welfare, 2018. Alcohol and other drug treatment services in Australia: 2016−17. Drug treatment series no. 31. Cat. no. HSE 207. Australian Institute of Health and Welfare, Canberra.

Australian Institute of Health and Welfare, 2019. Mental health services − in brief 2019. Cat. no. HSE 228. Australian Institute of Health and Welfare, Canberra.

Backlund, E., Sorlie, P. D., Johnson, N. J., 1999. A comparison of the relationships of education and income with mortality: the national longitudinal mortality study. Soc. Sci. Med. 49, 1373−1384.

Chamberlain, P., Goldney, R., Delfabbro, P., Gill, T., Dal Grande, L., 2009. Suicidal ideation. The clinical utility of the K10. Crisis 30, 39−42.

Christensen, H., Griffiths, K. M., Jorm, A. F., 2004. Delivering interventions for depression by using the internet: randomised controlled trial. Br. Med. J. 328, 265.

Crum, R. M., Storr, C. L., Ialongo, N., Anthony, J. C., 2008. Is depressed mood in childhood associated with an increased risk for initiation of alcohol use during early adolescence? Addict. Behav. 33, 24−40.

Cuijpers, P., van Straten, A., van Schaik A., Andersson, G., 2009. Psychological treatment of depression in primary care: a meta-analysis. Br. J. Gen. Pract., doi: 10.3399/bjgp09X395139.

Dean, K., Green, M. J., Laurens, K. R., Kariuki, M., Tzoumakis, S., Sprague, T., Lenroot, R., Carr, V. J., 2018. The impact of parental mental illness across the full diagnostic spectrum on externalising and internalising vulnerabilities in young offspring. Psychol. Med. 48, 2257−2263.

Dooley, D., Prause, J., Ham-Rowbottom, K. A., 2000. Underemployment and depression: longitudinal relationships. J. Health Soc. Behav. 41, 421−436.

Frijters, P., Johnston, D. W., Shields, M. A., 2014. The effect of mental health on employment: evidence from Australian panel data. Health Econ. 23, 1058−1071.

Green, M. J., Tzoumakis, S., Laurens, K. R., Dean, K., Kariuki, M., Harris, F., Brinkman, S. A., Carr, V. J., 2019. Early developmental risk for subsequent childhood mental disorders in an Australian population cohort. Aust. N. Z. J. Psychiatry 53, 304−315.

Herman, D. B., Susser, E. S., Struening, E. L., Link, B. L., 1997. Adverse childhood experiences: are they risk factors for adult homelessness? Am. J. Public Health 87, 249−255.

Johnson, T. P., Freels, S. A., Parsons, J. A., Vangeest, J. B., 1997. Substance abuse and homelessness: social selection or social adaptation? Addiction 92, 437−445.

Kyriacou, D. N., Anglin, D., Taliaferro, E., Stone, S., Tubb, T., Linden, J. A., Muelleman, R., Barton, E., Kraus, J. F., 1999. Risk factors for injury to women from domestic violence. N. Engl. J. Med. 341, 1892−1898.

Lee, S., Tsang, A., Breslau, J., Aguilar-Gaxiola, S., Angermeyer, M., Borges, G., Bromet, E., Bruffaerts, R., de Girolamo, G., Fayyad, J., Gureje, O., Haro, J. M., Kawakami, N., Levinson, D., Oakley Browne, M. A., Ormel, J., Posada-Villa, J., Williams, D. R., Kessler, R. C., 2009. Mental disorders and termination of education in high-income and low- and middle-income countries: epidemiological study. Br. J. Psychiatry 194, 411−417.

Marmorstein, N. R., Iacono, W. G., Malone, S. M., 2010. Longitudinal associations between depression and substance dependence from adolescence through early adulthood. Drug Alcohol Depend. 107, 154−160.

Miller, W. R., Walters, S. T., Bennett, M. E., 2001. How effective in alcoholism treatment in the United States? J. Stud. Alcohol 62, 211−220.

Mitchell, W., Wray, L. R., Watts, M., 2019. Macroeconomics. Red Globe Press, London.

Morrison, D. S., 2009. Homelessness as an independent risk factor for mortality: results from a retrospective cohort study. Int. J. Epidemiol. 38, 877−883.

Rehm, J., 2013. Alcohol use disorders and mortality: a systematic review and meta-analysis. Addiction 108, 1562−1578.

Russ, T. C., Stamatakis, E., Hamer, M., Starr, J. M., Kivimäki, M., Batty, G. D., 2012. Association between psychological distress and mortality: individual participant pooled analysis of 10 prospective cohort studies. Br. Med. J. 345, e4933.

Shelton, K. H., Taylor, P. J., Bonner, A., van den Bree, M., 2009. Risk factors for homelessness: evidence from a population-based study. Psychiatr. Serv. 60, 465−472.

Sorlie, P. D., Rogot, E., 1990. Mortality by employment status in the National Longitudinal Mortality Study. Am. J. Epidemiol. 132, 983−992.

Stevens, A., Radcliffe, P., Sanders, M., Hunt, N., 2008. Early exit: estimating and explaining early exit from drug treatment. Harm Reduct. J. 5, 13.

Tyrer, P., Morgan, J., Van Horn, E., Jayakody, M., Evans, K., Brummell, R., White, T., Baldwin, D., Harrison-Read, P., Johnson, T., 1995. A randomised controlled study of close monitoring of vulnerable psychiatric patients. Lancet 345, 756−759.

Wilkins, R., 2004. The extent and consequences of underemployment in Australia. Melbourne Institute working paper no. 16/04. The University of Melbourne, Melbourne.

Wilkins, R., 2006. Personal and job characteristics associated with underemployment. Aust. J. Labour Econ. 9, 371−393.

Section 2. Modelling the impact of COVID-19

The demographic, economic, health services, and psychological impacts of the continuing COVID-19 pandemic (see, e.g., Atkinson et al., 2020; Brooks et al., 2020; Charles-Edwards et al., 2020; Moreno et al., 2020) were modelled as abrupt changes in multiple flows directly affected by infection control measures (lockdowns, social distancing, international and interstate travel restrictions), including: 1) a decrease in the number of people arriving from overseas per year; 2) increases in the per capita rates at which people transition from employment (including underemployment) to unemployment and from full employment to underemployment; 3) reductions in per capita rates of non-acute mental health services provision (including general practitioner services, psychiatrist and allied health services, public hospital outpatient services, and private mental health services); and 4) an increase in the incidence of moderate to very high psychological distress resulting from social dislocation unrelated to job loss (e.g., working from home, not participating in recreational activities, restricted social gatherings) and anxiety about potential unemployment (Dooley et al., 1988). Parameter values determining the scale and duration of these direct effects (which begin on 1 March 2020 and decay over time) were estimated via constrained optimisation, implemented in Stella Architect ver. 1.9.4, using population projections for 2025 and 2030 reported by Charles-Edwards et al. (2020) and data on labour force status, psychological distress, and Medicare-subsidised mental health services usage (general practitioner, psychiatrist, and allied health services) available from the Australian Bureau of Statistics (2021a, b) and the Australian Government Department of Health (http://medicarestatistics.humanservices.gov.au/statistics/mbs_item.jsp) (see figure S26).

National survey data for the period after mid-March 2020 (when stringent infection control measures were first implemented in Australia) indicate that after increasing significantly in the initial months of the pandemic, the prevalence of psychological distress fell relatively rapidly as public health restrictions eased and unemployment and underemployment declined towards pre-pandemic levels in late 2020 and early 2021 (Biddle et al., 2020a, b; Australian Bureau of Statistics, 2021b; Biddle and Gray, 2021). This suggests that a subset of people experiencing increased psychological distress due to COVID-19-related social and economic disruption did not develop persistent mental disorders and had the capacity to recover quickly when the direct impacts of the pandemic subsided. As a means of accommodating this distressed but resilient subpopulation, we allow the per capita spontaneous recovery rate (see section 1.2) to increase as the prevalence of psychological distress increases above that observed immediately prior to the start of the pandemic (i.e., from 1 March 2020). During

early-mid 2020, decreasing social connectedness and increasing unemployment and underemployment drive an increase in the prevalence of moderate to very high psychological distress via increased distress incidence; however, because many people becoming psychologically distressed have the capacity to recover more rapidly than those with persistent mental disorders, the per capita spontaneous recovery rate also increases. As the direct social and economic effects of the pandemic abate, the incidence of moderate to very high psychological distress declines, and the higher per capita recovery rate results in a relatively rapid decrease in distress prevalence, consistent with the empirical data (see figure S26; note that as distress prevalence declines, the per capita spontaneous recovery rate also declines as acutely distressed people without persistent disorders recover).

Among people becoming psychologically distressed as a result of the pandemic, the risk of intentional self-harm and suicide is assumed to increase gradually (rather than immediately), consistent with evidence for a significant association between unemployment duration and suicidal behaviour (Milner et al., 2012) and a delay in the impact of economic recessions on suicide mortality (Garcy and Vågerö, 2013). From 1 March 2020, intentional self-harm hospitalisation rates (per year) for people aged 15 years and above are calculated as $s_i M_i + \theta s_i (p/q) H_i + f(t) s_i (1 - p/q) H_i$, where M_i and H_i are the numbers of people in age group i experiencing low (mild) psychological distress and moderate to very high psychological distress, respectively, s_i is the per capita suicide attempt rate per year for mildly distressed people in age group i, θ is the suicide attempt rate ratio among moderately to highly distressed people who were distressed before the pandemic began or who would have become distressed if the pandemic had not occurred, q is the prevalence of moderate to very high psychological distress, p is the prevalence of moderate to very high psychological distress immediately prior to 1 March 2020 (i.e., before the implementation of infection control measures), and $f(t)$ is a graphical function specifying the suicide attempt rate ratio for people becoming psychologically distressed as a result of the pandemic at time t years since 1 March 2020 (the default function, shown in figure S27, is based on Milner et al., 2012).

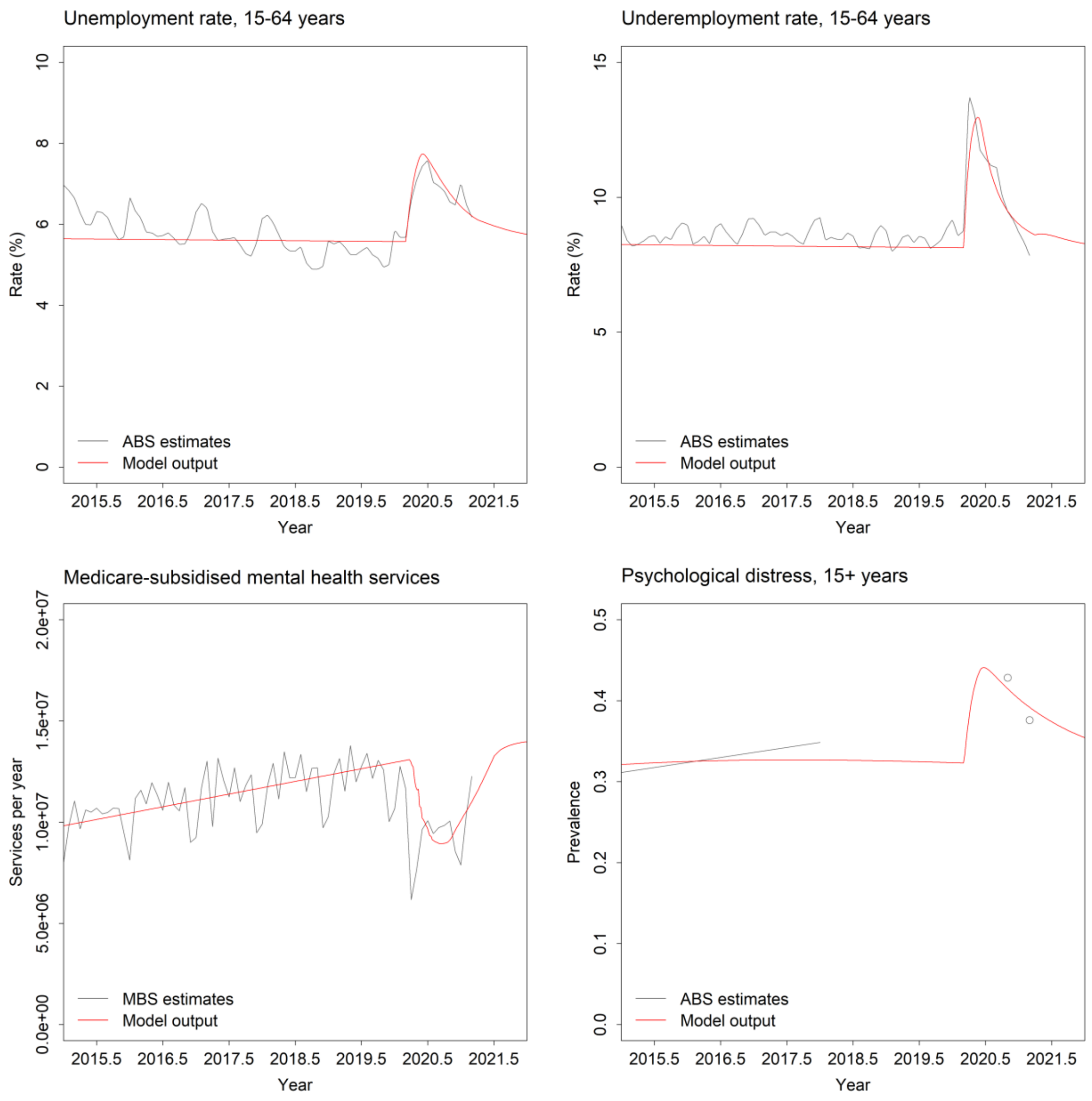

Figure S26. Modelled effects of the COVID-19 pandemic on unemployment, underemployment, mental health services usage, and psychological distress (red lines). Data published by the Australian Bureau of Statistics (ABS; 2021a, b) and the Australian Government Department of Health used to fit the model are shown in grey. (MBS = Medicare Benefits Schedule.)

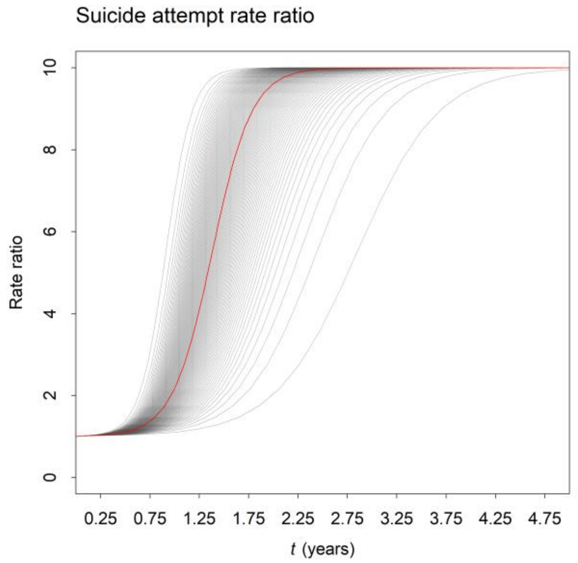

Figure S27. Graphical functions specifying the suicide attempt rate ratio for people becoming psychologically distressed as a result of the COVID-19 pandemic at time t years since 1 March 2020. The grey lines were generated for use in sensitivity analyses by multiplying the input time to the default function, shown in red, by a normal random variable with mean 1 and standard deviation 0.2, effectively compressing or stretching the function along the horizontal axis.

References

Atkinson, J., Song, Y. J. C., Merikangas, K. R., Skinner, A., Prodan, A., Iorfino, F., Freebairn, L., Rose, D., Ho, N., Crouse, J., Zipunnikov, V., Hickie, I. B., 2020. The science of complex systems is needed to ameliorate the impacts of COVID-19 on mental health. Front. Psychiatry 11, 606035.

Australian Bureau of Statistics, 2021a. Labour force, Australia, Mar 2021. Cat. no. 6202.0. Australian Bureau of Statistics, Canberra.

Australian Bureau of Statistics, 2021b. Household Impacts of COVID-19 Survey, Mar 2021. Cat. no. 4940.0. Australian Bureau of Statistics, Canberra.

Biddle, N., Edwards, B., Gray, M., Sollis, K., 2020a. Tracking outcomes during the COVID-19 pandemic (August 2020) — divergence within Australia. Centre for Social Research and Methods, Australian National University, Canberra.

Biddle, N., Gray, M., 2021. Tracking outcomes during the COVID-19 pandemic (January 2021) — cautious optimism. Centre for Social Research and Methods, Australian National University, Canberra.

Biddle, N., Edwards, B., Gray, M., Sollis, K., 2020a. Tracking outcomes during the COVID-19 pandemic (August 2020) — divergence within Australia. Centre for Social Research and Methods, Australian National University, Canberra.

Brooks, S. K., Webster, R. K., Smith, L. E., Woodland, L., Wessely, S., Greenberg, N., Rubin, G. J., 2020. The psychological impact of quarantine and how to reduce it: rapid review of the evidence. Lancet 395, 912−920.

Charles-Edwards, E., Wilson, T., Bernard, A., Wohland, P., 2020. How will COVID-19 impact Australia's future population? A scenario approach. Working paper no. 2020/03. Queensland Centre for Population Research, University of Queensland, Brisbane.

Dooley, D., Catalano, R., Rook, K. S., 1988. Personal and aggregate unemployment and psychological symptoms. J. Soc. Issues 44, 107−123.

Garcy, A. M., Vågerö, D., 2013. Unemployment and suicide during and after a deep recession: a longitudinal study of 3.4 million Swedish men and women. Am. J. Public Health 103, 1031−1038.

Milner, A., Page, A., LaMontagne, A. D., 2013. Long-term unemployment and suicide: a systematic review and meta-analysis. PLoS ONE 8, e51333.

Moreno, C., Wykes, T., Galderisi, S., Nordentoft, M., Crossley, N., Jones, N., Cannon, M., Correll, C. U., Byrne, L., Carr, S., Chen, E. Y. H., Gorwood, P., Johnson, S., Kärkkäinen, H., Krystal, J. H., Lee, J., Lieberman, J., López-Jaramillo, C., Männikkö, M., Phillips, M. R., Uchida, H., Vieta, E., Vita, A., Arango, C., 2020. How mental health care should change as a consequence of the COVID-19 pandemic. Lancet Psychiatry 7, 813−824.

Section 3. Intervention definitions and parameter assumptions

Table S1. Intervention definitions and parameter assumptions used in previous models provided as examples. Parameters determining the direct effects of each intervention can be modified via an interactive model interface, enabling users to assess the impact of parameter assumptions on model outputs.

Intervention	Description
**1. Mental health and suicide prevention interventions**	
Post-attempt assertive aftercare	Post-attempt assertive aftercare is an active outreach and enhanced contact program to reduce readmission in those presenting to services after a suicide attempt. It includes individually tailored contact, solution focused counselling, and motivations to adherence to follow-up treatments and continuity of contact.<br><br>Parameters determining the direct effects of this intervention are:<br><br>*Maximum post-attempt care rate* – the maximum proportion of patients hospitalised for a suicide attempt receiving post-attempt care. The default value (1) assumes that post-attempt care will be provided to all patients hospitalised for a suicide attempt (after an initial scale up period).<br><br>*Post-attempt care effect* – the proportion of potential repeat suicide attempts expected among patients receiving post-attempt care. The default value (0.398) implies that 39.8% of repeat attempts that would have occurred without post-attempt care actually occur when post-attempt care is provided; i.e., post-attempt care is assumed to prevent 60.2% of potential repeat suicide attempts. The default estimate is derived from Hvid et al. (2011, Nord. J. Psychiatry 65, 292-298).<br><br>*Effect duration (weeks)* – the average time in weeks after a suicide attempt that post-attempt care has an effect on the probability of a repeat attempt. The default value of 52.1 weeks implies that, on average, post-attempt care reduces the repeat self-harm rate for 1 year after an attempt. After this time, post-attempt care is assumed to have no impact on the suicide attempt rate.<br><br>*Repeat self-harm rate per year* – the probability that a person will self-harm in the year after a suicide attempt without post-attempt care. The default value (0.179) implies that 17.9% of people hospitalised for self-harm will re-attempt within 1 year (i.e., assuming they don't receive post-attempt care); this estimate is derived from Carroll et al. (2014, PLoS ONE 9, e89944).
Social connectedness programs	Community support programs and services that increase social connectedness, reducing isolation and enhancing resilience in the face of adversity.<br><br>Parameters determining the direct effects of this intervention are:<br><br>*Sense of Community Index target* – the maximum Sense of Community Index (SCI) that could be achieved with the planned social

	connectedness program(s), where the SCI ranges from 0 to 12, with 12 corresponding to the highest possible sense of community (see Chipuer and Pretty, 1999, J. Community Psychol. 27, 643-658). The default value (9.61) corresponds to an increase in the SCI (relative to the baseline value, 9.15) of 5% (Handley et al., 2012, Soc. Psychiatry Psychiatr. Epidemiol. 47, 1281-1290).<br><br>*Effect on distress* – the multiplicative effect of a 1-unit increase in the SCI on distress onset rates. The default value (0.640) is derived from Handley et al. (2012, Soc. Psychiatry Psychiatr. Epidemiol. 47, 1281-1290), and implies that a 1-unit increase in the SCI reduces the rate at which people become psychologically distressed by 36.0%.<br><br>*Effect on attempt lethality* – the multiplicative effect of a 1-unit increase in the SCI on suicide attempt lethality. The default value (0.964) assumes that an increase in the SCI from its baseline value (9.15) to 12 would reduce suicide attempt lethality by 10%.<br><br>*Social connectedness decay rate* – the rate at which the SCI decreases to its baseline value (9.15) after investment in social connectedness programs ends. The default value (0.5) implies that the increase in the SCI due to social connectedness programs (i.e., the difference between the current SCI and baseline value) decreases at a rate of 50% per year.
Community-based acute care services	Responsive clinical mental health services delivered by community mental health teams. People in suicidal crisis may call and request either a home-based visit or a centre-based visit, depending on their level of functioning and risk.<br><br>Parameters determining the direct effects of this intervention are:<br><br>*Maximum self-referral rate* – the maximum proportion of people presenting to emergency departments for suicidal ideation or behaviour who would self-refer to community-based acute care services (i.e., if these were made available). The default value (0.7) assumes that 70% of people in suicidal crisis who would normally present to an emergency department would contact community-based services instead.<br><br>*Effect on self-harm rate* – the proportion of potential re-presentations for suicidal ideation or behaviour expected among patients referred to community-based acute care services. The default value (0.398) implies that 39.8% of re-presentations that would have occurred if a person in crisis was treated in an emergency department actually occur when community-based care is provided; i.e., community-based acute care is assumed to prevent 60.2% of potential re-presentations for suicidal ideation or behaviour. The default value is derived from Hvid et al. (2011, Nord. J. Psychiatry 65, 292-298). (Note that the default value is the estimated effect of post-attempt care on the repeat self-harm rate, but that the duration of effect of community-based acute care is assumed to be much shorter than that of post-attempt care.)<br><br>*Effect duration (weeks)* – the average time in weeks after referral to services that community-based acute care has an effect on the probability of repeat episodes of suicidal ideation or behaviour (the default value is 2 weeks).<br><br>*Re-presentation rate per year* – the expected number of re-presentations for suicidal ideation or behaviour in the year after an initial suicide-related emergency department attendance. The default value (3.84) is derived from Perera et al. (2018, Med. J. Aust. 208, 348-353), and implies that in the year after presenting to an emergency department for suicidal ideation or behaviour, patients will re-present 3.8 times (on average).
Technology-enabled crisis response	Provides paramedics, police, and acute care teams with technology to enable rapid assessment of patients in the community (e.g., video link to a mental health clinician via tablet). Patients requiring immediate treatment are referred to hospital as usual; patients with less severe symptoms

	are referred to community mental health services, with follow up in 1-2 days.<br><br>Parameters determining the direct effects of this intervention are:<br><br>*Effect on self-harm rate* – the proportion of potential re-presentations for suicidal ideation or behaviour expected among patients treated by a crisis response team. The default value (0.398) implies that 39.8% of re-presentations that would have occurred if a person in crisis was treated in an emergency department actually occur when crisis response care is provided in the community; i.e., community-based crisis response care is assumed to prevent 60.2% of potential re-presentations for suicidal ideation or behaviour. The default value is derived from Hvid et al. (2011, Nord. J. Psychiatry 65, 292-298). (Note that the default value is the estimated effect of post-attempt care on the repeat self-harm rate, but that the duration of effect of crisis response care is assumed to be much shorter than that of post-attempt care.)<br><br>*Effect duration (weeks)* – the average time in weeks that technology-enabled crisis response care has an effect on the probability of repeat episodes of suicidal ideation or behaviour (the default value is 2 weeks).<br><br>*Re-presentation rate per year* – the expected number of re-presentations for suicidal ideation or behaviour in the year after an initial suicide-related emergency department attendance. The default value (3.84) is derived from Perera et al. (2018, Med. J. Aust. 208, 348-353), and implies that in the year after presenting to an emergency department for suicidal ideation or behaviour, patients will re-present 3.8 times (on average).
Technology-enabled coordinated care	Technology-enabled coordinated care involves the use of online technology to facilitate delivery of multidisciplinary team-based care, in which medical and allied health professionals consider all relevant treatment options and collaboratively develop an individual treatment and care plan for each patient. Online technology enables enhanced coordination of care and facilitates communication between medical and allied health professionals, since each health professional involved in the care of a patient has access to the same information about that patient's treatment history.<br><br>Parameters determining the direct effects of this intervention are:<br><br>*Maximum rate per service* – the maximum proportion of mental health services provided that involve technology-enabled coordinated care. This proportion will depend on the number of medical and allied mental health professionals adopting online care coordination technologies, as well as the number of patients consenting to the use of these technologies in the management of their care (i.e., take-up among service providers and patients). The default value (0.7) assumes that when fully implemented, technology-enabled coordinated care will be provided in 70% of mental health services completed.<br><br>*Effect on recovery rate* – the multiplicative effect of technology-enabled coordinated care on the per-service recovery rate (i.e., the probability that a patient's level of psychological distress will decrease after receiving treatment). The default estimate (1.177) is derived from Woltmann et al. (2012, Am. J. Psychiatry 169, 790-804), and implies that technology-enabled coordinated care increases the per-service probability of a reduction in psychological distress by 17.7%.<br><br>*Effect on referrals to specialised care* – the multiplicative effect of technology-enabled coordinated care on general practitioners' rates of referral to specialised psychiatric care (i.e., psychiatrists and allied mental health services). The default value (1.266) implies that technology-enabled coordinated care increases the per-consultation probability that a general practitioner will refer a patient with high or very high psychological distress to specialised psychiatric care by 26.6%, and is derived from Badamgarav et al. (2003, Am. J. Psychiatry 160, 2080-2090). Note that technology-enabled coordinated care is assumed to have no effect on the referral rate for patients with moderate psychological

	distress.<br><br>*Effect on disengagement* – the multiplicative effect of technology-enabled coordinated care on rates of disengagement from mental health services (including disengagement while waiting for services and disengagement resulting from dissatisfaction with services received). The default estimate (0.520) is derived from Badamgarav et al. (2003, Am. J. Psychiatry 160, 2080-2090), and implies that technology-enabled coordinated care reduces rates of disengagement by 48.0%.<br><br>*Effect on referrals to AOD services* – the multiplicative effect of technology-enabled coordinated care on the rate of referral of patients with a substance abuse disorder to alcohol and drug treatment services. The default value (1.1) assumes an increase in the rate of referral of 10%; i.e., patients with a substance abuse disorder receiving technology-enabled coordinated care are 10% more likely to be referred to alcohol and drug treatment services than patients with a substance abuse disorder receiving usual care.<br><br>*Effect on substance use relapse rate* – the multiplicative effect of coordinated treatment of co-occurring substance abuse and mental disorders on the substance use relapse rate (i.e., the probability that a patient treated for a substance use disorder will relapse when treatment is completed). The default value (0.869) is derived from Kikkert et al. (2018, J. Subst. Abuse Treat. 95, 35-42), and implies that coordinated substance abuse and psychiatric treatment reduces the rate of substance use relapse by 13.1% (i.e., compared to substance abuse treatment alone).<br><br>*Effect on employment initiation* – the multiplicative effect of technology-enabled coordinated care on the rate at which unemployed patients gain employment (through referral to employment services). The default value (1.1) assumes an increase in the employment initiation rate of 10%; i.e., unemployed patients receiving technology-enabled coordinated care are 10% more likely to gain employment than unemployed patients receiving usual care.<br><br>*Effect on exiting homelessness rate* – the multiplicative effect of technology-enabled coordinated care on the rate at which homeless patients secure housing (through referral to homelessness services). The default value (1.1) assumes a 10% increase in the rate that patients exit homelessness (equal to the inverse of the duration of homelessness); i.e., homeless patients receiving technology-enabled coordinated care are 10% more likely to secure housing than homeless patients receiving usual care.<br><br>*Effect on psychiatric services capacity* – the multiplicative effect of technology-enabled coordinated care on the total capacity of specialised psychiatric services (i.e., the maximum number of services that can be provided by psychiatrists and allied mental health providers per year). The default value (1.1) assumes an increase in services capacity of 10%.
Post-discharge peer support	Based on the Hospital to Home (H2H) program, this intervention involves peer workers (i.e., individuals with their own lived experience of mental illness and recovery) providing individualised practical and emotional support to patients discharged from psychiatric hospital care (see Scanlan et al., 2017, BMC Psychiatry 17, 307).<br><br>Parameters determining the direct effects of this intervention are:<br><br>*Maximum peer support rate* – the maximum proportion of patients receiving post-discharge peer support. The default value (1) implies that all patients discharged from psychiatric hospital care are referred to peer support services once this intervention is fully implemented.<br><br>*Effect on ED presentations* – the proportion of potential mental health-related emergency department (ED) presentations expected among patients receiving peer support. The default value (0.567) is derived from Lawn et al. (2008, J. Mental Health 17, 498-508), and implies that

	56.7% of post-discharge ED presentations that would have occurred if a patient had received usual post-discharge care actually occur when peer support is provided; i.e., peer support is assumed to prevent 43.3% of post-discharge ED presentations.<br><br>*Effect on self-harm rate* – the proportion of potential suicide attempts expected among patients receiving post-discharge peer support. The default value (0.398) implies that 39.8% of suicide attempts that would have occurred if a patient received usual post-discharge care actually occur when peer support is provided; i.e., peer support is assumed to prevent 60.2% of post-discharge suicide attempts. The default value is derived from Hvid et al. (2011, Nord. J. Psychiatry 65, 292-298). (Note that the default value is the estimated effect of post-attempt care on the repeat self-harm rate, but that the default duration of post-discharge peer support is assumed to be much shorter than that of post-attempt care.)<br><br>*Effect on disengagement* – the multiplicative effect of peer support on the rate that patients disengage from mental health services after discharge from psychiatric hospital care. The default value (0.632) is derived from Craig et al. (2004, J. Mental Health 13, 59-69), and implies that peer support reduces the post-discharge disengagement rate by 36.8%.<br><br>*Duration of support (weeks)* – the average time in weeks that patients receive peer support after discharge from psychiatric hospital care (the default value is 6 weeks).
General practitioner (GP) training	Short (1-2 days) training programs aimed at reducing suicidal ideation through referral to specialised psychiatric services. This includes people who may be thinking about suicide for the first time or have survived a previous attempt.<br><br>Parameters determining the direct effects of this intervention are:<br><br>*Maximum training rate* – the maximum proportion of mental health-related GP services provided by GPs who have attended a training program (this value increases as the number of GPs attending training programs increases). The default value (0.7) implies that at most 70% of mental health-related GP services will be provided by a GP who has attended a training program.<br><br>*GP training effect* – the multiplicative effect of GP training on the rate of referral to psychiatrist and allied mental health services. The default value (1.4375) implies that GPs who have received training are 1.44 times more likely to refer patients with high or very high levels of psychological distress (Kessler 10 scores 22 and above) than a GP who has not received training. The default estimate is derived from Pfaff et al. (2001, Med. J. Aust. 174, 222-226).
Safety planning	Safety planning aims to reduce suicidal behaviour through the provision of a specific plan for staying safe during crisis to suicidal patients presenting to an emergency department. The modelled intervention also includes up to 2 follow-up phone calls to monitor suicide risk and support treatment engagement (see Stanley et al., 2018, JAMA Psychiatry 75, 894-900).<br><br>Parameters determining the direct effects of this intervention are:<br><br>*Maximum rate per ED visit* – the maximum proportion of suicide-related emergency department presentations in which a safety plan is provided. The default value (0.7) assumes that a safety plan is provided to 70% of patients presenting to an emergency department for suicidal ideation or behaviour.<br><br>*Effect on self-harm rate* – the proportion of potential re-presentations for suicidal ideation or behaviour expected among patients provided with a safety plan. The default value (0.847) implies that 84.7% of suicide-related re-presentations that would have occurred without safety planning actually occur when a safety plan is provided; i.e., safety planning is assumed to prevent 15.3% of potential re-presentations for suicidal ideation or behaviour. The

	default value is derived from Miller et al. (2017, JAMA Psychiatry 74, 563-570).<br><br>*Effect duration (weeks)* – the average time in weeks after a suicide-related emergency department presentation that safety planning has an effect on the probability of re-presentations for suicidal ideation or behaviour (the default is 8 weeks).<br><br>*Re-presentation rate per year* – the expected number of re-presentations for suicidal ideation or behaviour in the year after an initial suicide-related emergency department attendance. The default value (3.84) is derived from Perera et al. (2018, Med. J. Aust. 208, 348-353), and implies that in the year after presenting to an emergency department for suicidal ideation or behaviour, patients will re-present 3.8 times (on average).
Community-based education programs (awareness campaigns)	Population-wide mental health education programs aimed at reducing stigma, improving recognition of suicide risk, and encouraging help-seeking. This intervention increases the per capita rates at which people perceive a need for mental health services and seek help from a general practitioner or online services.<br><br>Parameters determining the direct effects of this intervention are:<br><br>*Effect on help seeking* – the multiplicative effect of community-based education programs on the rate (per year) that a psychologically-distressed person not engaged with mental health services will perceive a need for treatment and the rate that a person perceiving a need for care will seek help from a GP or online services. The default value (1.585) is derived from Jorm et al. (2003, Psychol. Med. 33, 1071-1079), and assumes that awareness campaigns will increase the rate of help seeking for mental health problems by 58.5%.<br><br>Effect of community support programs – the multiplicative effect of a 1-unit increase in the Sense of Community Index (SCI) on the impact of mental health education programs. The default value (1.034) assumes that an increase in the SCI from its baseline value (9.15) to the highest possible value (12) would increase the effect of a mental health education program on the rate of help seeking by 10%. Note that this parameter only has an effect if mental health education programs and community support programs (which increase the SCI) are implemented together (potentially with different starting times).
Family psychoeducation and support	Provision of education and support to families and carers of patients presenting to or engaged with mental health services, with the aim of supporting family or carer involvement in the management of diagnosed mental disorders.<br><br>Parameters determining the direct effects of this intervention are:<br><br>*Maximum rate per patient* – the maximum proportion of patients with a chronic mental disorder who would consent to having their family involved in the management of their care. The default value (0.553) implies that family education and support would be provided to a maximum of 55.3% of patients with a chronic mental disorder, and is derived from Shimazu et al. (2011, Br. J Psychiatry 198, 385-390).<br><br>*Effect on recovery rate* – the multiplicative effect of family education and support on the recovery rate among patients with a chronic mental disorder treated by a GP, psychiatrist, or allied mental health professional. The default value (2.52) is derived from Shimazu et al. (2011, Br. J Psychiatry 198, 385-390), and implies that family education and support increase the per-service probability of recovery by a factor of 2.5.
Safe space services	Based on the United Kingdom's Safe Haven café model, this intervention provides an alternative point of contact with mental health services for people experiencing acute psychological distress who may otherwise present to an emergency department.<br><br>Parameters determining the direct effects of this intervention are:<br><br>*Maximum self-referral rate* – the maximum proportion of people presenting to emergency departments for suicidal ideation or behaviour who would

	self-refer to a safe space alternative (i.e., if it were made available). The default value (0.7) assumes that 70% of people in suicidal crisis who would normally present to an emergency department would present to a safe space alternative instead.<br><br>*Effect on self-harm rate* – the proportion of potential re-presentations for suicidal ideation or behaviour expected among patients referred to a safe space service. The default value (0.398) implies that 39.8% of re-presentations that would have occurred if a person in crisis was treated in an emergency department actually occur when care is provided in a safe space alternative; i.e., care in a safe space alternative is assumed to prevent 60.2% of potential re-presentations for suicidal ideation or behaviour. The default value is derived from Hvid et al. (2011, Nord. J. Psychiatry 65, 292-298). (Note that the default value is the estimated effect of post-attempt care on the repeat self-harm rate, but that the duration of effect of safe space care is assumed to be much shorter than that of post-attempt care.)<br><br>*Effect duration (weeks)* – the average time in weeks that care provided in a safe space service has an effect on the probability of repeat episodes of suicidal ideation or behaviour (the default value is 2 weeks).<br><br>*Re-presentation rate per year* – the expected number of re-presentations for suicidal ideation or behaviour in the year after an initial suicide-related emergency department attendance. The default value (3.84) is derived from Perera et al. (2018, Med. J. Aust. 208, 348-353), and implies that in the year after presenting to an emergency department for suicidal ideation or behaviour, patients will re-present 3.8 times (on average).
Services capacity growth	*GP mental health services* — multiplies the annual rate of increase in the total number of mental health-related GP consultations that can be completed per week. The default value (1) corresponds to the business as usual case, in which services capacity continues to increase at the current rate, estimated using Medicare Benefits Schedule (MBS) data for 2011-2019 assuming services were operating at (near-) maximum capacity over this period.<br><br>*Psychiatrist and allied services* — multiplies the annual rate of increase in the total number of psychiatrist and allied services that can be provided per week. The default value (1) corresponds to the business as usual case, in which services capacity continues to increase at the current rate, estimated using Medicare Benefits Schedule (MBS) data for 2011-2019 assuming services were operating at (near-) maximum capacity over this period.<br><br>*Psychiatric hospital care* — multiplies the annual rate of increase in the maximum number of public psychiatric hospital admissions per week. The default value (1) corresponds to the business as usual case, in which services capacity continues to increase at the current rate, estimated using hospital separations data published by the Australian Institute of Health and Welfare (available at: https://www.aihw.gov.au/reports-data/health-welfare-services/mental-health-services/data).<br><br>*Community mental health* — multiplies the annual increase in the total number of community mental health service contacts that can be provided per week. The default value (1) corresponds to the business as usual case, in which services capacity continues to increase at the current rate, estimated using data for the period 2011-2019 published by the Australian Institute of Health and Welfare (available at: https://www.aihw.gov.au/reports-data/health-welfare-services/mental-health-services/data).<br><br>*Private hospital care* — multiplies the annual rate of increase in the maximum number of private psychiatric hospital admissions per week. The default value (1) corresponds to the business as usual case, in which services capacity continues to increase at the current rate, estimated using hospital separations data published by the Australian Institute of Health and Welfare (available at: https://www.aihw.gov.au/reports-data/health-welfare-services/mental-health-services/data).<br><br>*Private outpatient services* — multiplies the annual increase in the total number of private outpatient services that can be provided per week. The default value (1) corresponds to the business as usual case, in which services capacity continues to increase at the current rate, estimated using data for the period 2014–2019 published by the Australian Institute of Health and Welfare (available at: https://www.aihw.gov.au/reports-data/health-welfare-services/mental-health-services/data).
2. Social determinants	

Employment programs	Programs designed to stem rapidly increasing unemployment due to the COVID-19 pandemic (e.g., the JobKeeper Payment). This intervention reduces the increase in the per capita job loss rate resulting directly from the pandemic. The per capita rate of employment initiation can also be increased (or decreased); however, the default settings assume that employment programs have no direct effect on employment initiation.<br><br>Parameters that can be modified in this intervention are:<br><br>*Starting year* — the year in which employment programs commence (the default is 2020.33, or May 2020).<br><br>*Implementation time (years)* — the time required for employment programs to be fully implemented (the default is 0.167 years, or 2 months).<br><br>*Program duration (years)* — the duration of employment programs (the default is 0.917 years, 11 months. With the starting year set at 2020.33, this will set Employment programs to end in April 2021).<br><br>*Effect on job loss* — the multiplicative effect of employment programs on the increase in the job loss rate due to the COVID-19 pandemic. The default value (0.2) assumes that employment programs will reduce the increase in the per capita job loss rate by 80%.<br><br>*Effect on employment initiation* — the multiplicative effect of employment programs on the per capita employment initiation rate. The default value (1) assumes no effect of employment programs on employment initiation. Values greater than 1 increase the rate at which people secure employment (this could be achieved through an expansion of the public service, for example).
Education programs	Programs providing support to students who have become unemployed due to the COVID-19 pandemic, enabling them to continue studying. This intervention reduces the per capita rate that students discontinue post-secondary study as a direct result of job loss. The per capita rate of enrolment in post-secondary study among people aged 15-24 years can also be increased (or decreased); however, the default settings assume that education programs do not directly affect the enrolment rate.<br><br>Parameters that can be modified in this intervention are:<br><br>*Starting year* — the year in which education programs commence (the default is 2021.5, or July 2021).<br><br>*Implementation time (years)* — the time required for education programs to be fully implemented (the default is 0.167 years, or 2 months).<br><br>*Program duration (years)* — the duration of education programs (the default is 5 years).<br><br>*Effect on discontinuation* — the multiplicative effect of education programs on the proportion of students discontinuing post-secondary study due to job loss. The default value (0.1) assumes that education programs reduce the proportion of students discontinuing study after becoming unemployed by 90%.<br><br>*Effect on enrolment* — the multiplicative effect of education programs on the per capita enrolment rate for 15-24-year-olds. The default value (1) assumes no effect of education programs on enrolment. Values greater than 1 increase the rate at which people aged 15-24 years enroll in post-secondary study (e.g., a value of 1.2 would increase the per capita enrolment rate by 20%).
Better Access	

	Reform of the existing *Better Access to Psychiatrists, Psychologists and General Practitioners through the MBS* (Better Access) initiative to provide patients with access to a greater number of specialised mental health care consultations per year. This intervention increases the flow of people with a perceived need for mental health care into psychiatrist and allied mental health services.<br><br>Parameters that can be modified in this intervention are:<br><br>*Starting year* — the year in which the reformed Better Access initiative commences (the default is 2020.75, or October 2020).<br><br>*Implementation time (years)* — the time after commencement required for the reformed Better Access initiative to be fully implemented (the default is 0.167 years, or 2 months).<br><br>*Program duration (years)* — the duration of the reformed Better Access initiative (the default is set to 100,000 years, ensuring that investment continues until the end of the simulation).<br><br>*Services per week* — the average number of specialised mental health care services provided per patient per week. The default value (1) assumes that patients attend 1 consultation per week, so that a patient attending a total of 4 consultations (for example) is assumed to do so over a period of 4 weeks.<br><br>*Additional services per patient* — the mean number of additional specialised mental health care services provided per patient per year under the reformed Better Access scheme. The default value (4) assumes that patients will attend an additional 4 consultations per year when the cap on the number of consultations per patient is increased.
Online services	Increased investment in online (self-help) services providing support to people with relatively low care needs. This intervention increases the per capita rate that people with a perceived need for mental health care access online services.<br><br>Parameters that can be modified in this intervention are:<br><br>*Starting year* — the year in which increased investment in online services commences (the default is 2021.5, or July 2021).<br><br>*Implementation time (years)* — the time required to scale up investment in online services (the default is 0.167 years, or 2 months).<br><br>*Program duration (years)* — the duration of increased investment in online services (the default is 5 years).<br><br>*Increase in services usage* — the multiplicative effect of increased investment in online services on the rate that people perceiving a need for mental health care access those services. The default value (1.2) assumes that increased investment will increase the per capita rate of access to online services by 20%.
Childcare programs	Programs designed to provide greater childcare availability and affordability. This enables people who are not in the labour force and would like a job, but are currently prevented by childcare reasons and of those people who are not in the labour force and do not want a job, but would if childcare was available to enter the labour force. This effect of this intervention is based on the Australia Bureau of Statistics' Survey of Income and Housing, Australia, 2017-18 data to calculate the labour force participation rates ratio pre and post intervention, and this effect is calculated for different sex and age groups. This effect increases the transition rate from not in the labour force into unemployment.<br><br>Parameters that can be modified in this intervention are:<br><br>*Starting year* — the year in which increased investment in childcare programs commence (the default is 2021.5, or July 2021).

	Implementation time (years) — the time required to scale up investment in childcare programs (the default is 0.167 years, or 2 months).<br><br>*Program duration (years)* — the duration of increased investment in childcare programs (the default is 5 years).
Welfare programs	Programs designed to provide payments to those in unemployment or underemployment who are in financial distress due to the COVID-19 pandemic (e.g., the JobSeeker Payment). By reducing financial distress, this intervention reduces the prevalence of psychological distress in this population.<br><br>Parameters that can be modified in this intervention are:<br><br>*Starting year* — the year in which increased investment in welfare programs commences (the default is 2020.33, or May 2020).<br><br>*Implementation time (years)* — the time required to scale up investment in welfare programs (the default is 0.167 years, or 2 months).<br><br>*Program duration (years)* — the duration of increased investment in welfare programs (the default is 1 years).<br><br>*Effect on psychological distress* — the multiplicative effect of financial security (defined as an ability to pay utility bills on time) on the per capita rate of moderate-very high psychological distress onset. The default value (0.377) is derived from Kiely et al. (2015, Soc. Psychiatry Psychiatr. Epidemiol. 50, 909-91), and implies that an increase in welfare payments sufficient to provide financial security to someone experiencing financial hardship would reduce their risk of moderate-very high psychological distress by 62.3%.<br><br>*Proportional increase in welfare payment* — the multiplicative increase in mean household income from Government pensions and allowances resulting from an increase in welfare payments. The default value (1.176) is derived from ABS data on household financial resources indicating that the addition of the Coronavirus supplement to the JobSeeker Payment in April 2020 increased mean household income from Government pensions and allowances by 17.6% (see Australian Bureau of Statistics, 2020, Household financial resources, June 2020. Australian Bureau of Statistics, Canberra).
Female jobs creation programs	Programs designed to increase the jobs available to and hence increase the per-capita employment rates of females. This intervention increases the rate at which females transition from unemployment to underemployment and from unemployment to employment by a multiplier. This multiplier can be modified across different age groups for females.<br><br>Parameters that can be modified in this intervention are:<br><br>*Starting year* — the year in which increased investment in female jobs creation programs commences (the default is 2021.5, or July 2021).<br><br>*Female 15-24* — increases the monthly transition rates from unemployment to employment and from unemployment to underemployment for females aged 15-24 years old by a multiplier. The default value (1) corresponds to the business as usual case, in which the transition rates follow the trends from 2010-2020 data published by the Australia Bureau of Statistics' catalogue Labour Force, Australia (https://www.abs.gov.au/statistics/labour/employment-and-unemployment/labour-force-australia).<br><br>*Female 25-44* — increases the monthly transition rates from unemployment to employment and from unemployment to underemployment for females aged 25-44 years old by a multiplier. The default value (1) corresponds to the business as usual case, in which the transition rates follow the trends from 2010-2020 data published by the Australia Bureau of Statistics' catalogue Labour Force, Australia (https://www.abs.gov.au/statistics/labour/employment-and-unemployment/labour-force-australia).

	Female 45-64 — increases the monthly transition rates from unemployment to employment and from unemployment to underemployment for females aged 45-64 years old by a multiplier. The default value (1) corresponds to the business as usual case, in which the transition rates follow the trends from 2010-2020 data published by the Australia Bureau of Statistics' catalogue Labour Force, Australia (https://www.abs.gov.au/statistics/labour/employment-and-unemployment/labour-force-australia).
Reducing childhood adversity (scenario testing)	Multiplies the rates at which children (aged 0–14 years) at low and moderate risk of developing a mental disorder transition to moderate and high levels of risk. The default value (1) corresponds to the baseline case, in which no new policy interventions act to change these rates directly (i.e., business as usual). Values lower than 1 reduce the rates that children transition to higher levels of risk (this could be achieved with the introduction of new early life prevention programs, for example).
Reducing domestic violence (scenario testing)	Multiplies domestic violence rates (incidents reported per year) among people aged 15 years and above. The default value (1) corresponds to the baseline case, in which no new policy interventions act to change these rates directly (i.e., business as usual). Values less than 1 reduce rates of domestic violence (this may be achieved with the introduction of new prevention programs, for example).
Reducing homelessness (scenario testing)	Multiplies the age-specific rates at which people in secure housing enter homelessness. The default value (1) corresponds to the baseline case, in which no new policy interventions act to change these rates directly (i.e., business as usual). Values less than 1 reduce rates of homelessness (this could be achieved with the introduction of new housing programs, for example).
